# Eligibility for early rhythm control in patients with atrial fibrillation in the UK Biobank

**DOI:** 10.1136/heartjnl-2022-321196

**Published:** 2022-07-14

**Authors:** Shinwan Kany, Victor Roth Cardoso, Laura Bravo, John A Williams, Renate Schnabel, Larissa Fabritz, Georgios V Gkoutos, Paulus Kirchhof

**Affiliations:** 1 Department of Cardiology, University Medical Center Hamburg-Eppendorf, Hamburg, Germany; 2 Cardiovascular Disease Initiative, The Broad Institute of MIT and Harvard, Cambridge, Massachusetts, USA; 3 Partner site Hamburg/Kiel/Lübeck, German Center for Cardiovascular Sciences (DZHK), Hamburg, Germany; 4 Institute of Cardiovascular Sciences, University of Birmingham, Birmingham, UK; 5 Institute of Translational Medicine, University Hospitals Birmingham NHS Foundation Trust, Birmingham, UK; 6 Midlands Site, Health Data Research UK, Birmingham, UK; 7 Institute of Cancer and Genomic Sciences, University of Birmingham, Birmingham, UK

**Keywords:** Atrial Fibrillation, Stroke, Catheter Ablation

## Abstract

**Objective:**

The Early Treatment of Atrial Fibrillation for Stroke Prevention (EAST-AFNET4) trial showed a clinical benefit of early rhythm-control therapy in patients with recently diagnosed atrial fibrillation (AF). The generalisability of the results in the general population is not known.

**Methods:**

Participants in the population-based UK Biobank were assessed for eligibility based on the EAST-AFNET4 inclusion/exclusion criteria. Treatment of all eligible participants was classified as early rhythm-control (antiarrhythmic drug therapy or AF ablation) or usual care. To assess treatment effects, primary care data and Hospital Episode Statistics were merged with UK Biobank data.

Efficacy and safety outcomes were compared between groups in the entire cohort and in a propensity-matched data set.

**Results:**

AF was present in 35 526/502 493 (7.1%) participants, including 8340 (988 with AF <1 year) with AF at enrolment and 27 186 with incident AF during follow-up. Most participants (22 003/27 186; 80.9%) with incident AF were eligible for early rhythm-control.

Eligible participants were older (70 years vs 63 years) and more likely to be female (42% vs 21%) compared with ineligible patients. Of 9004 participants with full primary care data, 874 (9.02%) received early rhythm-control. Safety outcomes were not different between patients receiving early rhythm-control and controls. The primary outcome of EAST-AFNET 4, a composite of cardiovascular death, stroke/transient ischaemic attack and hospitalisation for heart failure or acute coronary syndrome occurred less often in participants receiving early rhythm-control compared with controls in the entire cohort (HR 0.82, 95% CI 0.71 to 0.94, p=0.005). In the propensity-score matched analysis, early rhythm-control did not significantly decrease of the primary outcome compared with usual care (HR 0.87, 95% CI 0.72 to 1.04, p=0.124).

**Conclusion:**

Around 80% of participants diagnosed with AF in the UK population are eligible for early rhythm-control. Early rhythm-control therapy was safe in routine care.

**WHAT IS ALREADY KNOWN ON THIS TOPIC**

- The randomised controlled Early Treatment of Atrial Fibrillation for Stroke Prevention (EAST-AFNET4) trial showed that early rhythm-control in recently diagnosed atrial fibrillation (AF) leads to a reduced composite outcome of death from cardiovascular causes, stroke or hospitalisation with worsening of heart failure or acute coronary syndrome.

**WHAT THIS STUDY ADDS**

- The results of the randomised controlled EAST-AFNET4 trial are applicable to 80.9% of patients developing AF in the general population. Early rhythm control as practised in the UK Biobank population appeared safe. Early rhythm control was not associated with fewer outcome events in a propensity-score matched analysis (HR 0.87, 95% CI 0.72 to 1.04, p=0.124) likely due to smaller absolute effect estimator and low power.

**HOW THIS STUDY MIGHT AFFECT RESEARCH, PRACTICE OR POLICY**

- Consideration of early rhythm control should be part of the management in all patients with recently diagnosed AF.

## Introduction

Atrial fibrillation (AF) is the most common arrhythmia, affecting more than 33 million people in 2010.^1^ Disease prevalence is anticipated to rise in the next decades due to the increased ageing of the population and better screening methods. Oral anticoagulation (OAC) and therapy of concomitant cardiovascular conditions prevent most ischaemic strokes in patients with AF.^2^ However, even on optimal current therapy, many patients with AF experience cardiovascular death, including sudden death, stroke or worsening of heart failure.^3^

Due to earlier trials showing no effect of rhythm control therapy compared with rate control on cardiovascular outcomes,^4^ combined with safety concerns and a higher hospitalisation rate on antiarrhythmic drug therapy (AAD), most patients with newly diagnosed AF are initially managed without rhythm control therapy.^5^ The ‘Early Treatment of Atrial Fibrillation for Stroke Prevention’ (EAST-AFNET4) trial showed that systematic initiation of rhythm control therapy early after the diagnosis of AF reduces cardiovascular death, stroke or hospitalisation for heart failure or acute coronary syndrome by 21% compared with the current paradigm restricting rhythm control to symptomatic participants.^6^ The generalisability of those treatment effects is not known. Currently, it is not known how many patients with AF would be eligible for early rhythm control and how the safety outside of controlled trial settings would be.

To answer these questions, we interrogated the UK Biobank (UKB), a prospective cohort study enrolling a population-based sample of middle-aged individuals in the UK.

## Methods

### UK Biobank

The UKB is a prospective, population-based cohort study of 502 493 participants enrolled between 2006 and 2010 in the UK.^7^ Randomly selected persons aged 40–69 years were invited to participate. Comprehensive medical information was collected at baseline. Informed consent was obtained before enrolment. Data on incidence of disease and mortality were obtained via UK death registers and inpatient records from the Hospital Episode Statistics (HES) for England, Scottish Morbidity Records and Patient Episode Database for Wales. Primary care (general practioner (GP)) data providing information on treatment is available for around 45% of the participants using the TPP, Vision and EMIS databases. Self-reported outcomes were collected at baseline. Follow-up was obtained until March 2021.

### Patient involvement

The patient involvement in the UKB is widely known. This analysis was conducted anonymised data from the UKB. There was no specific patient involvement in this data science project. There is no plan to share study results with patients.

### Definition of eligibility for early rhythm control

To determine eligibility for early rhythm control, the inclusion and exclusion criteria of the EAST-AFNET4 trial were applied as far as possible in the UKB^8^ (see online supplemental definitions file). International Classification of Diseases- 10 (ICD-10) coding was used to define the presence of a diagnosis. Eligibility was defined as a diagnosis of AF within 1 year and presence of cardiovascular conditions and risk factors, namely age >75 years, a previous stroke or transient ischaemic attack (TIA), or two of the following: age >65 years, female sex, hypertension, diabetes mellitus, severe coronary artery disease, heart failure, chronic kidney disease or peripheral artery disease. Exclusion criteria were the combination of female sex and age <45 years, drug abuse, previous AF ablation, severe mitral stenosis, prosthetic mitral valve, hepatic dysfunction, thyroid dysfunction without treatment and severe renal dysfunction (stage V or dialysis). Eligibility for early rhythm control was determined in the entire UKB dataset. To compare outcomes in participants receiving early rhythm control to others, all participants with sufficient information on therapy, that is, those with GP data, were considered. Participants were assigned to early rhythm control if they received either AAD therapy (amiodarone, dronedarone, flecainide, propafenone or sotalol) or AF ablation within 1 year of diagnosing AF. Those not receiving rhythm control within 1 year of AF diagnosis were assigned to usual care. Efficacy and safety outcomes were compared between groups.

10.1136/heartjnl-2022-321196.supp2 Supplementary data

### Outcomes

Outcomes in this analysis were aligned with the primary outcome of the EAST-AFNET4 trial.^6^ The primary efficacy outcome was a composite of cardiovascular death, stroke or TIA, or hospitalisation due to worsening of heart failure or acute coronary syndrome. The main safety outcome was defined as a composite of stroke, death or a serious adverse event related to early rhythm control (non-fatal cardiac arrest, drug-induced bradycardia, atrioventricular block, torsade de pointes tachycardia, pericardial tamponade, and preprocedural bleeding, or blood pressure event).

### Statistical analysis

#### Data sources and versions

This project was approved as UKB project 31 224. The UKB data used were sourced from the main field data, bulk hospital inpatient data (see previous, called HES for simplicity) and primary care records (GP) with prescription data. Participants that died within 6 months after the date of AF were excluded from the main efficacy analysis to ensure adequate time for treatment effects to affect mortality, similar to previous analyses.^9^ Additionally, the 6-month threshold was used to exclude patients who were diagnosed shortly before their death and therefore only received usual care.

A sensitivity analysis limiting follow-up to March 2020 was conducted to evaluate the influence of the COVID-19 pandemic. Another sensitivity analysis for safety and efficacy included every patient and all events to provide comprehensive safety information on early rhythm control.

### Propensity-score matching and falsification analysis

To reduce the effect of confounders of the outcomes of interest, participants were propensity-score matched based on a number of parameters, namely sex, hypertension, heart failure, coronary artery disease, history of myocardial infarction, chronic kidney disease, stroke/TIA, peripheral artery disease, diabetes, obstructive sleep apnoea, dyslipidaemia, chronic obstructive pulmonary disease, malignancy, history of alcohol abuse, valvular heart disease, age, body mass index, CHA_2_DS_2_VASc score, chronic kidney disease, dilated cardiomyopathy, gastrointestinal bleeding, gastrointestinal ulcer, history of endocarditis, hyperthyroidism, hypertrophic cardiomyopathy, hypothyroidism, myocardial infarction, osteoporosis, pulmonary embolism, smoking and OAC. The distance applied is Mahalanobis distance,^10^ and the nearest sample matching method was used. All resulting matches were assessed to ensure a standardised difference under 20% for each variable. To detect potential bias between groups, a falsification analysis of 23 random outcomes in the unmatched and the propensity-score matched group was performed.

### Statistical reporting

Numerical parameters were reported as median (1st–3rd quartile) for non-normal variables and mean (SD) for normal variables. An Anderson-Darling test was performed when the number of elements was equal or above 5000. For all other analyses, the Shapiro-Wilk test was used. The Kruskal-Wallis Rank test was used to compare non-normal variables, and one-way analysis of variance was employed for normal variables. For categorical variables, Fisher’s exact test was performed to check for independence. If there were more than 2×2 options, a p value Monte Carlo simulation was performed. Data processing was performed using Python V.3.7.4, and all analysis was conducted in R V.4.0.3. The propensity-score matching was performed using the MatchIt R package. The set of packages used can be found in the supplementary data.

## Results

### Eligibility for early rhythm control

**Figure 1:**
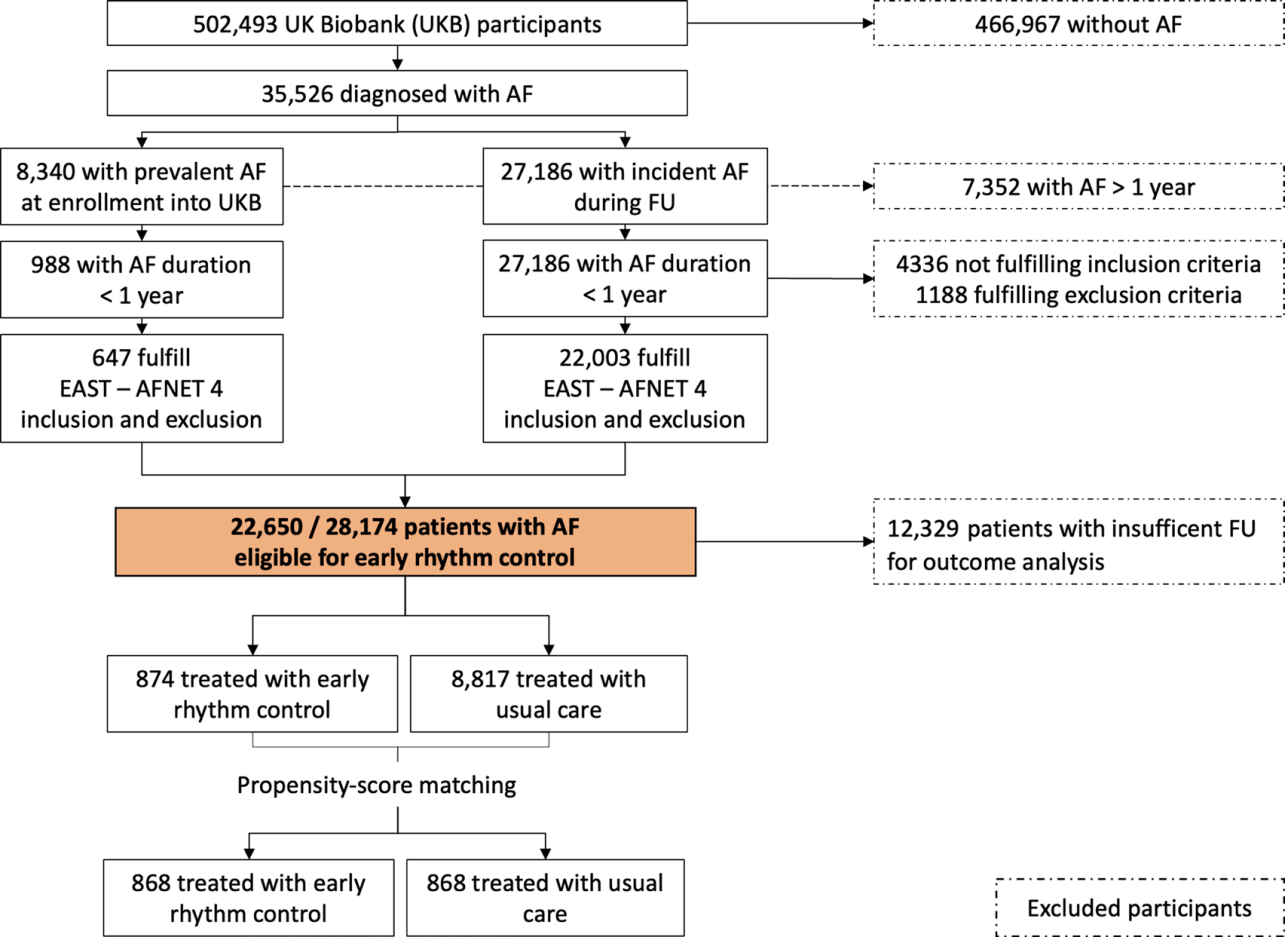
A flow chart of patient selection and proband selection. AF, atrial fibrillation; EAST-AFNET4, Early Treatment of Atrial Fibrillation for Stroke Prevention Trial; FU, follow-up

AF was diagnosed in 35 526/502 493 participants of the UKB (7.1%, figure 1). At the time of enrollment, AF was prevalent in 8340 participants, and 27 186 participants developed AF during follow-up. A total of 7352 participants had prevalent AF (>1 year) prior to their UKB enrolment and were excluded from further analyses. Out of 988 participants enrolled within a year of the first diagnosis of AF, 647 (65.49%) were eligible for early rhythm control therapy. Out of 27 186 participants with incident AF, 22 003 participants (80.94%) were eligible for early rhythm control. Eligible participants were older (70 years vs 63 years) and had more comorbidities reflected by higher CHA_2_DS_2_VASc scores (median 3 vs 1) than ineligible participants. Common conditions defining eligibility were age above 65 years, hypertension, heart failure and female sex if at least one of the named risk factors was present (online supplemental table 1). For analyses on the safety and effectiveness of treatment, 12 329 participants were excluded because of insufficient data on therapy, and 670 participants were excluded due to death within 6 months after enrolment.

10.1136/heartjnl-2022-321196.supp1 Supplementary data

### Baseline patient characteristics

Of the 9004 participants with therapy data, 874 participants (9.02%) were treated with early rhythm control, while 8817 participants were managed without early rhythm control (table 1). A propensity-score matching identified 868 pairs of participants receiving early rhythm control and usual care. Participants in the early rhythm control group were slightly younger (68 years vs 70 years, p<0.001) compared with those with usual care. OAC was initiated in 69% of participants receiving early rhythm control compared with 29% in usual care despite a median CHA_2_DS_2_-VASc score of 3 in both groups. In the matched analysis, the rate of OAC was comparable in both groups (69% vs 60%, p=0.554).

**Table 1:** Baseline characteristics of participants receiving early rhythm control or usual care in the overall cohort

	Early rhythm control (n=874)	Usual care (n=8817)	P value
Follow-up duration (years)	4.94 (2.99–6.92)	2.54 (0.58–5.50)	<0.001
Age (years)	68 (64–70)	70 (66–73)	<0.001
Body mass index (kg/m²)	28.16 (25.27–31.51)	28.61 (25.67–32.21)	0.005
Days until treatment	45.00 (18–152.25)	–	–
Sex (male)	509 (58%)	5060 (57%)	0.641
Chronic kidney disease	67 (8%)	771 (9%)	0.313
Diabetes	113 (13%)	1563 (18%)	<0.001
Heart failure	526 (60%)	4562 (52%)	<0.001
Hypertension	803 (92%)	8094 (92%)	1.000
Peripheral vascular disease	37 (4%)	331 (4%)	0.458
History of myocardial infarction	119 (14%)	1189 (13%)	0.917
Severe coronary artery disease	285 (33%)	2493 (28%)	0.008
Stroke/transient ischaemic attack	61 (7%)	791 (9%)	0.052
Valvular disease	53 (6%)	391 (4%)	0.034
Dyslipidaemia	248 (28%)	2556 (29%)	0.725
Obstructive sleep apnoea	17 (2%)	327 (4%)	0.005
Chronic obstructive pulmonary disease	44 (5%)	815 (9%)	<0.001
Malignancy	135 (15%)	1823 (21%)	0.001
History of alcohol abuse	16 (2%)	283 (3%)	0.024
CHA₂DS₂-VASc Score (1st–3rd quartile)	3 (2–4)	3 (2–4)	<0.001
Anticoagulation	607 (69%)	2599 (29%)	<0.001
Beta blocker	671 (77%)	2687 (30%)	<0.001
Digoxin	128 (15%)	510 (6%)	0.066
Sodium channel blockers	177 (20%)	67 (1%)	<0.001
Potassium channel blockers	679 (78%)	224 (3%)	<0.001
Catheter ablation	92 (11%)	0 (0)	–

Participants treated with early rhythm control were more likely to receive treatment with beta blockers, digoxin or AAD, with sotalol (28.8%) and amiodarone (43%) being the most common drugs followed by flecainide (15.6%). After propensity-score matching, no differences in baseline characteristics were observed (online supplemental table 2). The follow-up duration was 4.94 (2.99–6.92) years in participants treated with early rhythm control control and 2.54 (0.58–5.50) years in participants treated usual care (p<0.001). After matching, follow-up was comparable with 4.92 (2.98–6.91) years and 4.13 (1.65–6.69) years, respectively.

### Safety of early rhythm control

**Figure 2:**
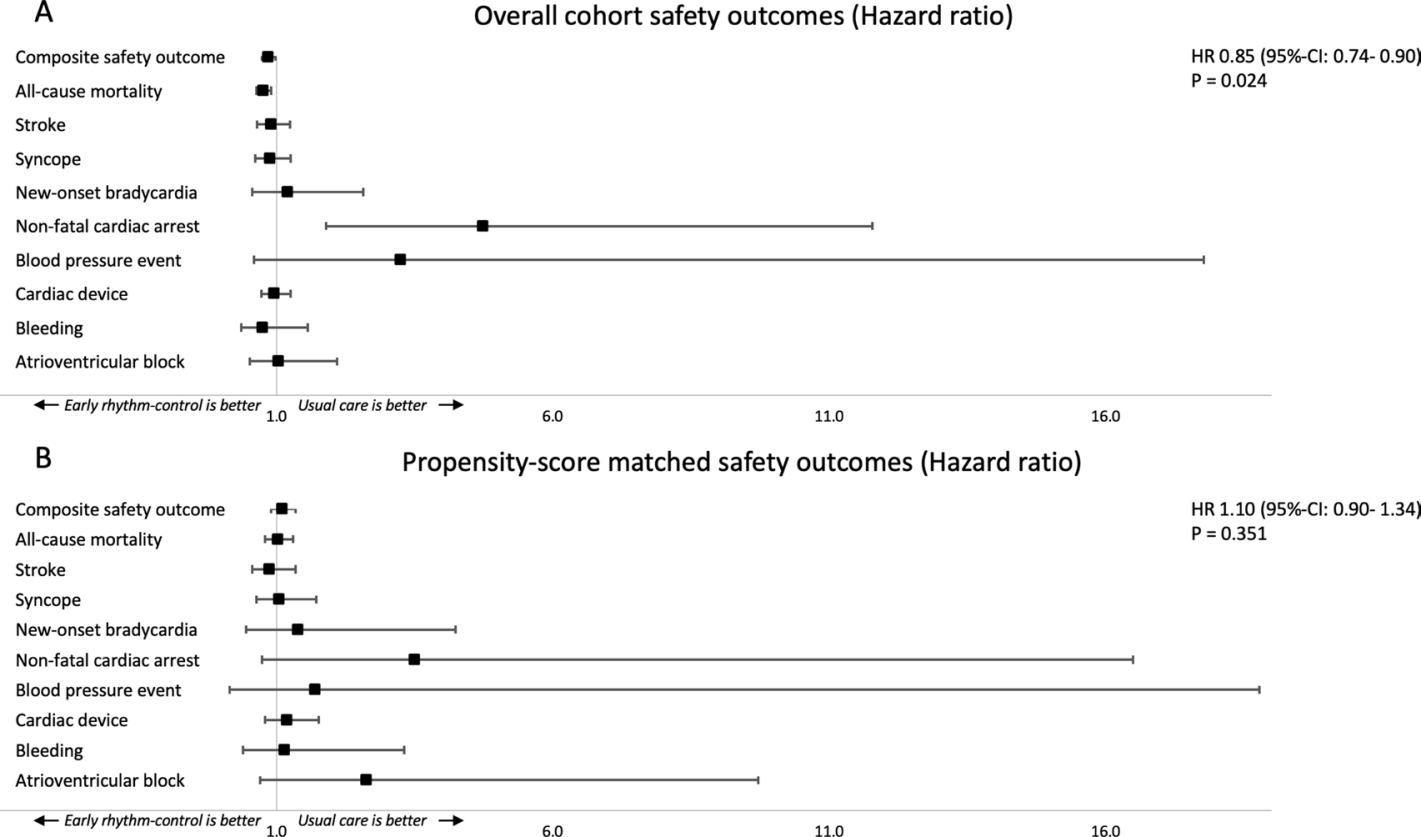
Primary safety outcome of a composite of stroke/transient ischaemic attack, all-cause death and adverse events related to rhythm control therapy of participants receiving either early rhythm control or usual care in the overall cohort (A) and a propensity-score matched analysis (B). The HRs and the 95% CIs are presented for the composite safety outcome and for its components.

The composite safety outcome (stroke, death or serious adverse event related to rhythm control therapy) occurred in 5.20 events/100 patient-years (table 2) in early rhythm control and 6.00 events/100 patient-years in participants treated with usual care (HR 0.85 (95% CI 0.74 to 0.98), p=0.024). After matching, no statistically significant differences in the composite safety outcome were observed (figure 2). The total mortality was lower in patients receiving early rhythm control in the unmatched (3.13 events/100 patient-years vs 4.05 events/100 patient-years; HR 0.76 (95% CI 0.64 to 0.90), p=0.002) but not in the matched comparison (3.14 events/100 patient-years vs 3.05 events/100 patient-years; HR 1.02 (95% CI 0.80 to 1.31), p=0.866).

**Table 2:** Safety outcomes in participants receiving early rhythm control or usual care

Outcome	Early rhythm-control (n=874)	Usual care (n=8817)	HR (95% CI)	P-value
Composite safety outcome	5.2	6.0	0.85 (0.74 to 0.98)	0.024
All-cause mortality	3.13	4.05	0.76 (0.64 to 0.90)	0.002
Stroke/TIA	0.96	1.04	0.90 (0.65 to 1.25)	0.533
Related to rhythm control therapy				
Syncope	0.80	0.89	0.88 (0.62 to 1.25)	0.479
New-onset bradycardia	0.18	0.15	1.21 (0.57 to 2.57)	0.624
Non-fatal cardiac arrest	0.18	0.04	4.73 (1.90 to 11.78)	0.001
Blood pressure event	0.04	0.01	3.25 (0.59 to 17.77)	0.175
Cardiac device	1.35	1.39	0.96 (0.73 to 1.26)	0.748
Bleeding	0.18	0.23	0.75 (0.36 to 1.57)	0.448
Atrioventricular block	0.20	0.19	1.04 (0.51 to 2.10)	0.919
Propensity-score matching				

Outcome	Early rhythm-control (n=868)	Usual care (n=868)	HR (95% CI)	P-value
Composite safety outcome	5.22	4.74	1.1 (0.90 to 1.34)	0.351
All-cause mortality	3.14	3.05	1.02 (0.80 to 1.31)	0.866
Stroke/TIA	0.95	1.09	0.87 (0.57 to 1.34)	0.533
Related to rhythm control therapy				
Syncope	0.81	0.77	1.05 (0.64 to 1.72)	0.841
New-onset bradycardia	0.18	0.13	1.39 (0.45 to 4.24)	0.565
Non-fatal cardiac arrest	0.18	0.05	3.50 (0.74 to 16.49)	0.113
Blood pressure event	0.05	0.03	1.70 (0.15 to 18.78)	0.664
Cardiac device	1.36	1.15	1.19 (0.80 to 1.76)	0.394
Bleeding	0.18	0.16	1.15 (0.40 to 3.32)	0.795
Atrioventricular block	0.20	0.08	2.63 (0.71 to 9.72)	0.147

Event rates are reported as events per 100 patient years. Composite safety outcome defined as all-cause mortality, stroke/TIA and events related to rhythm control therapy.

TIA, transient ischaemic attack.

### Efficacy of early rhythm control

**Figure 3:**
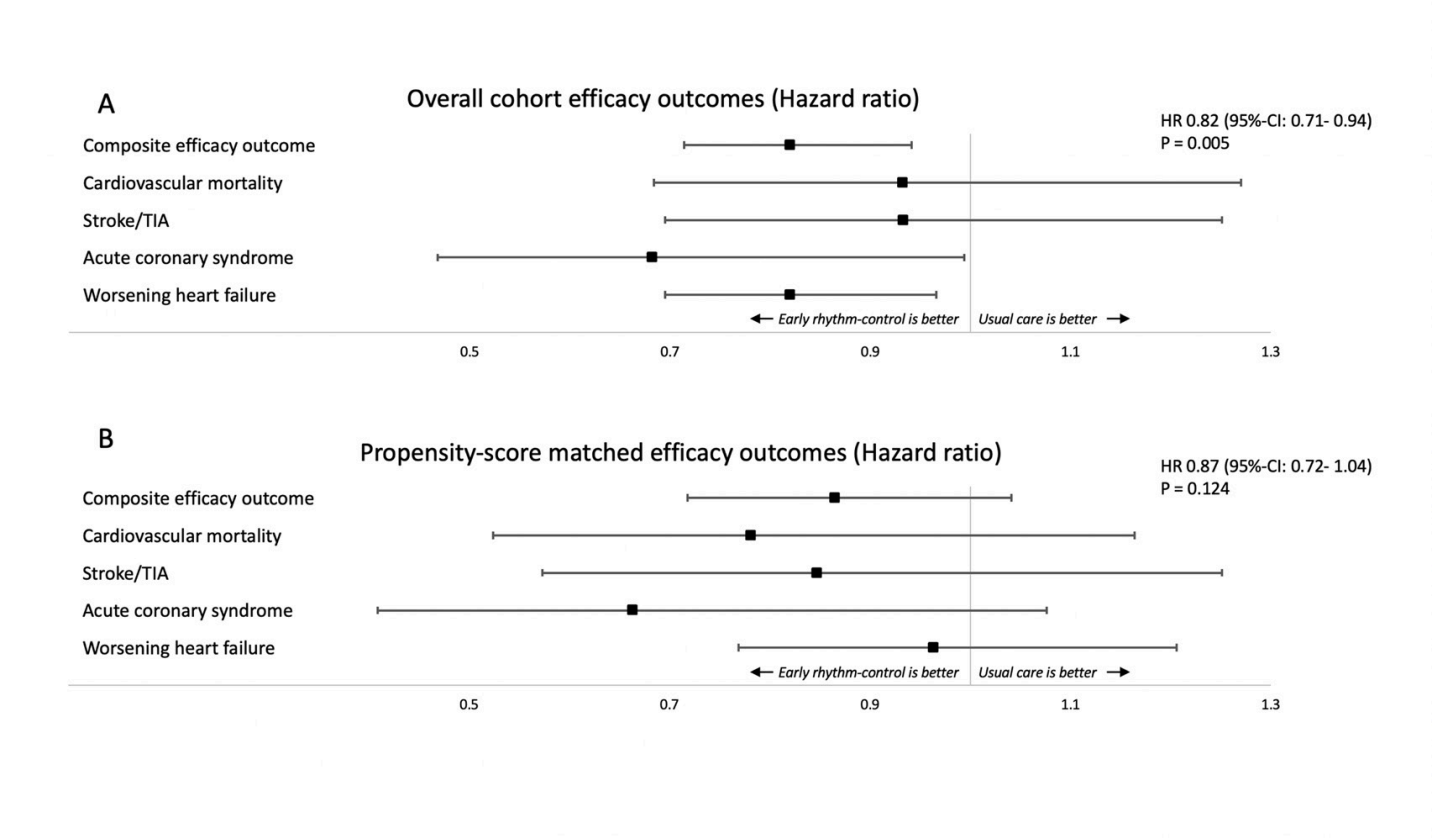
Main efficacy outcome of a composite of stroke/transient ischaemic attack, cardiovascular death, acute coronary syndrome and worsening of heart failure of participants receiving either early rhythm control or usual care in the overall cohort (A) and a propensity-score matched analysis (B). The HRs and the 95% CIs are presented for the composite efficacy outcome and for its components.

The composite outcome of cardiovascular death, stroke/TIA or hospitalisation for worsening heart failure or acute coronary syndrome occurred in 5.72 events/100 person-years (table 3) in early rhythm control and 6.92 events/100 person-years in controls (HR 0.82 (95% CI 0.71 to 0.94), p=0.005) in the unmatched cohort (figure 3). Significantly reduced components of this outcome were hospitalisation for heart failure and acute coronary syndrome in patient receiving early rhythm control. Nights spent in hospital were significantly less in participants receiving early rhythm control at 3.14±6.71 nights/year compared with 3.76±11.89 nights/year in controls (<0.001).

**Table 3:** Efficacy outcomes in participants receiving early rhythm control or usual care

Outcome	Early rhythm-control (n=874)	Usual care (n=8817)	HR (95% CI)	P-value
Composite efficacy outcome	5.72	6.92	0.82 (0.71 to 0.94)	0.005
Cardiovascular mortality	1.03	1.07	0.93 (0.68 to 1.27)	0.657
Stroke/TIA	1.17	1.23	0.93 (0.70 to 1.25)	0.642
Acute coronary syndrome	0.68	0.98	0.68 (0.47 to 0.99)	0.046
Worsening of heart failure	3.89	4.74	0.82 (0.70 to 0.97)	0.018
Nights spent in hospital per year*	3.14±6.71	3.76±11.89		<0.001
Propensity-score matching				

Outcome	Early rhythm control (n=868)	Usual care (n=868)	HR (95% CI)	P value
Composite efficacy outcome	5.73	6.60	0.87 (0.72 to 1.04)	0.124
Cardiovascular mortality	1.04	1.33	0.78 (0.52 to 1.16)	0.224
Stroke/TIA	1.16	1.37	0.85 (0.57 to 1.25)	0.403
Acute coronary syndrome	0.66	1.01	0.66 (0.41 to 1.08)	0.096
Worsening of heart failure	3.93	4.09	0.96 (0.77 to 1.21)	0.743
Nights spent in hospital per year	3.14±6.70	2.88±7.41		<0.001

Event rates are reported as events per 100 patient years. Composite efficacy outcome defined as cardiovascular death, stroke/TIA, hospitalisation for worsening heart failure or acute coronary syndrome.

P-values<0.05 were considered significant.

TIA, transient ischaemic attack.

**Figure 4:**
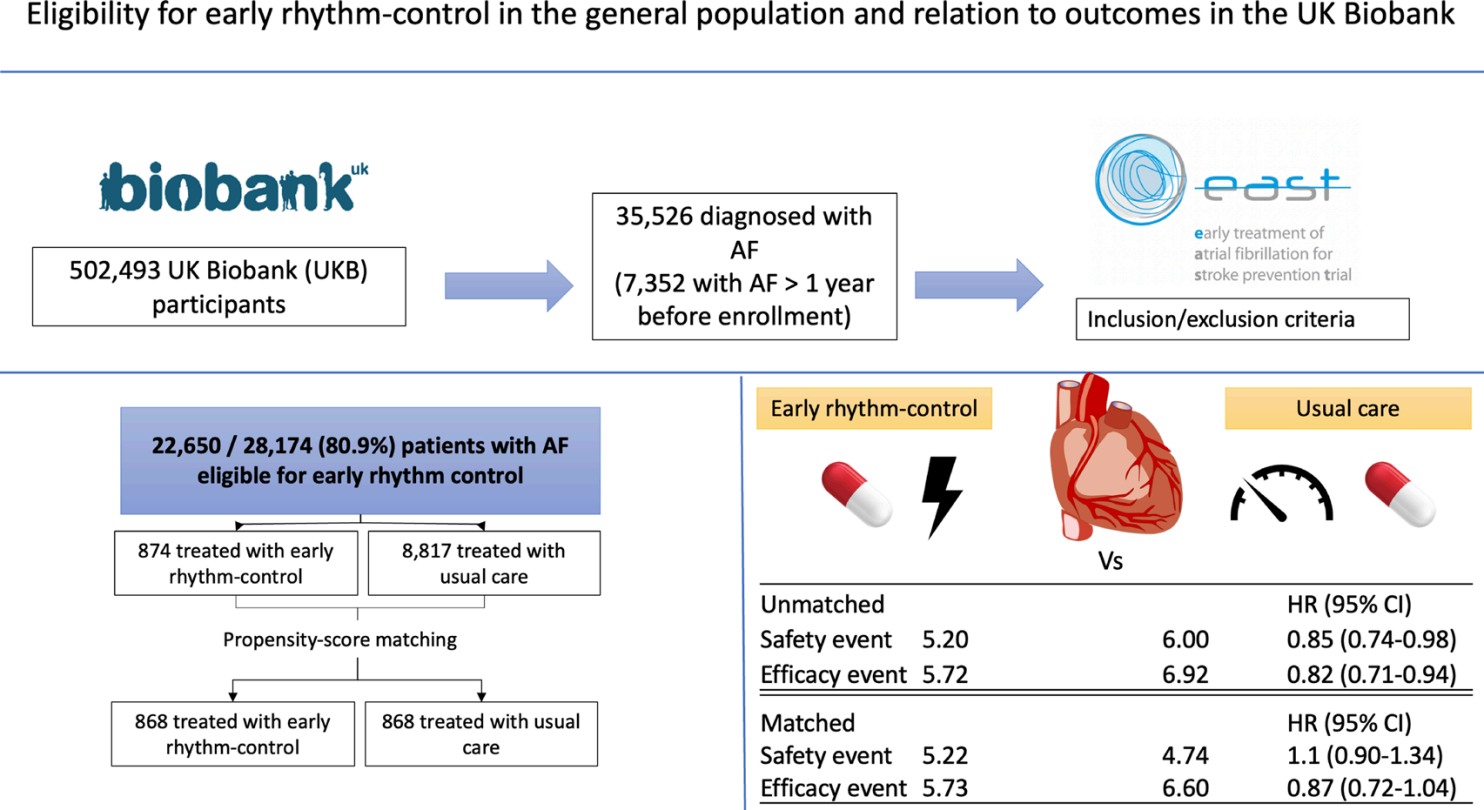
Summary figure showing the study design and the efficacy and safety outcomes in events/100 patient-years of EAST-AFNET4 eligible participants in the UK Biobank receiving either early rhythm control or usual care in the overall cohort and a propensity-score matched analysis. The HRs and the 95% CIs are presented in brackets. HR <1 favours early rhythm control. AF, atrial fibrillation; EAST-AFNET4, Early Treatment of Atrial Fibrillation for Stroke Prevention Trial.

Propensity-score matching analysis identified 5.73 events/100 person-years in participants treated with early rhythm control and 6.60 events/100 person-years in participants treated with usual care (HR 0.87, 95% CI 0.72 to 1.04, p=0.124). After matching, nights spent in hospital were higher in those who received early rhythm control with 3.14±6.70/year compared with participants treated with usual care 2.88±7.41 nights/year (p<0.001). An overview of the findings can be found in the central figure (figure 4).

The falsification analysis on the influence of early rhythm control on random outcomes demonstrated only 3 out of 23 outcomes in the overall cohort and only 1 out of 23 outcomes in the propensity-score matched analysis with significant differences between groups.

### Sensitivity analyses

To exclude the influence of reduced patient to healthcare provider contact during the COVID-19 pandemic, a sensitivity analysis limiting follow-up to March 2020 was carried out. Findings (online supplemental figures 1; 2) were consistent with the main analysis. The second sensitivity analysis was conducted without excluding participants due to death within 6 months of treatment as well as counting all events (online supplemental figures 3; 4). A reduction in the safety outcome was observed in early rhythm control. The efficacy outcomes in the overall cohort, as well in the matched cohort, were significantly reduced in early rhythm control (online supplemental figure 4).

10.1136/heartjnl-2022-321196.supp3 Supplementary data

10.1136/heartjnl-2022-321196.supp4 Supplementary data

10.1136/heartjnl-2022-321196.supp5 Supplementary data

10.1136/heartjnl-2022-321196.supp6 Supplementary data

## Discussion

### Main findings

This analysis of a population-based cohort yielded important findings (figure 4):

1. The majority of participants with newly diagnosed AF (approximately 80% in the UKB population) are eligible for early rhythm control therapy.
2. Early rhythm control appeared safe in this population-based sample receiving routine care.

Following the AFFIRM (Atrial Fibrillation Follow-up Investigation of Rhythm Management) and RACE (Rate Control versus Electrical Cardioversion for Persistent Atrial Fibrillation) trials, utilisation of rhythm control in AF therapy (electrical cardioversions and AAD) declined in the USA.^11^ Similar observations are made in Europe where 75%–90% of participants with AF are managed without rhythm control therapy.^12^ Recently, several relatively small, randomised trials identified a positive effect of rhythm control therapy using AF ablation in participants with AF and heart failure with reduced ejection fraction.^5^ The EAST-AFNET4 trial reported a 21% reduction in a composite outcome of death from cardiovascular causes, stroke or hospitalisation with worsening of heart failure or acute coronary syndrome in participants receiving rhythm control within 1 year of diagnosis compared with those who received usual care (HR 0.79; 96% CI 0.66 to 0.94; p=0.005).^6^ The clinical effectiveness of early rhythm control therapy was consistent in asymptomatic and symptomatic participants.^13^ These findings support a paradigm shift towards offering rhythm control therapy to all participants with recently diagnosed AF.

The present analysis demonstrates that most participants (approximately 80%), with newly detected AF, are eligible for early rhythm control in a large, population-based sample. Furthermore, our analyses confirm the safety of modern rhythm control therapy in routine care replicating the clinical effectiveness of early rhythm control reported in the EAST-AFNET4 trial.^6^ Our findings, based on a European population dataset, mirror those of a study related to the analysis of a nationwide South Korean health insurance dataset.^9^ This study included over 22 000 participants and demonstrated that rhythm control therapy, initiated within 1 year of diagnosis, was associated with a reduction of the composite outcome of cardiovascular death, stroke or heart failure admission or myocardial infarction (HR 0.81; 95% CI 0.71 to 0.93; p=0.002).^9^ The efficacy comparisons need to be interpreted with caution as the treatment allocation was not randomised. In our study, the matched analysis showed no difference in the primary efficacy outcome. However, this study was conducted to identify the number of eligible AF patients in the general population. Given that rhythm control therapy is rarely conducted in the UK,^14^ our early rhythm control cohort was approximately half of the EAST-AFNET4 population and therefore underpowered for efficacy studies.

The main difference between our study and the aforementioned studies lies with the investigated population. In our general population cohort, including participants who were never treated in hospital, the overall disease burden will be different compared with inpatient cohorts. Moreover, the early rhythm control therapies differed between the studies: in EAST-AFNET4 trial, the main antiarrhythmic drugs were flecainide and propafenone.^6^ In our analysis, sotalol and amiodarone were the main agents, while in the Korean dataset, amiodarone was the most common drug, closely followed by flecainide and propafenone.^9^ AF ablation was used in a minority of participants (approximately one-fourthin EAST-AFNET4 at 2 years, 11% in the propensity-matched population in this analysis, and only 1.6% in the Korean data set). In all three analyses, limited by the non-randomised treatment allocation in our analysis and in the Korean data set, early rhythm control was associated with fewer cardiovascular events during follow-up. AF ablation is more effective than AAD in preventing recurrent AF^15^ and may have positive effect on cardiovascular outcomes in patients with heart failure.^16^ It remains to be tested whether a broader use of AF ablation^15 17^ influences cardiovascular outcomes beyond the clinical effectiveness shown in EAST-AFNET4 trial. Another difference to the trial is that we use coding data for event analysis, which is dependent on data entry and does not reflect adjudication by clinicians. However, ICD-10 codes have been shown to be valid indicators for safety events in a hospital setting.^18^

Larger prospective studies would be helpful in assessing large-scale changes in treatment patterns to thoroughly investigate the safety of therapies. Recently, an analysis of a US administrative database found approximately 3/4 of all patients with newly diagnosed AF would be eligible for early rhythm control, which is comparable with our findings.^19^

Several factors could explain the low use of OAC in the UKB. For one, we capture a part of the whole population since we focused on optimal data quality and excluded those without primary care data. The data itself also does not allow to differentiate between a lack of OAC medication or missing data.

Additionally, a recent study using a database of primary care data in the UK from 2008 to 2018 showed that OAC use in the UK was around 43% and only recently increased to 71.6%.^14^ Nevertheless, our propensity-score matched analysis provides an estimate of the effects of early rhythm control therapy when the rates of OAC are similarly high.

### Strengths and limitations

The main strength of the current analysis is the use of a population-based sample, drawn in a country with an established, universal healthcare system and systematic follow-up. Thus, the number of participants eligible for early rhythm control should be a robust representation of a middle-aged European population and for populations with a similar age and disease profiles. Furthermore, the safety information is robust for routine care in the National Health Service and in comparable healthcare systems.

There are some limitations to this study. First, the comparison of outcomes is based on a non-randomised treatment allocation. Second, there were incomplete data available related to the therapy, limiting the size of the outcome cohort. Third, the focus on optimal data quality reduced the size of the matched cohorts. Fourth, we employed a 6-month blanking period since the observational design cannot account for all confounding factors, for example, if patients diagnosed with AF with a poor overall prognosis would be less likely to receive rhythm control therapy. Due to the nature of the UKB and the linkage to HES, this study is biased towards early rhythm control. Considering the standard of care over the last decade of UKB enrolment, when rhythm control was especially used in young AF patients with quality of life. Fifth, while UKB includes a population-based cohort, the UKB participants are relatively healthier, more likely to be from locations with higher socioeconomic standards and more likely to be female in comparison with the general population.^20^ To limit systematic bias, a repeated events analysis was performed.

## Conclusion

Approximately 80% of participants with recently diagnosed AF are eligible for early rhythm control in this population-based cohort. There was no safety signal attached to early rhythm control therapy, confirming the safety of early rhythm control found in the EAST-AFNET4 trial in a routine care setting. The rate of OAC in the EAST-AFNET4 trial eligible patients was low and highlights the need for anticoagulation of AF patients at risk for stroke.

Early rhythm control should become a routine part of the clinical management of most patients with recently diagnosed AF.

## Data Availability

Data are available in a public, open access repository. All data are available via the UK Biobank.

## References

[1] Chugh SS , Havmoeller R , Narayanan K , et al . Worldwide epidemiology of atrial fibrillation: a global burden of disease 2010 study. Circulation 2014;129:837–47. 10.1161/CIRCULATIONAHA.113.005119 24345399 PMC4151302

[2] Patel MR , Mahaffey KW , Garg J , et al . Rivaroxaban versus warfarin in nonvalvular atrial fibrillation. N Engl J Med 2011;365:883–91. 10.1056/NEJMoa1009638 21830957

[3] Marijon E , Le Heuzey J-Y , Connolly S , et al . Causes of death and influencing factors in patients with atrial fibrillation: a competing-risk analysis from the randomized evaluation of long-term anticoagulant therapy study. Circulation 2013;128:2192–201. 10.1161/CIRCULATIONAHA.112.000491 24016454

[4] Van Gelder IC , Hagens VE , Bosker HA , et al . A comparison of rate control and rhythm control in patients with recurrent persistent atrial fibrillation. N Engl J Med 2002;347:1834–40. 10.1056/NEJMoa021375 12466507

[5] Willems S , Meyer C , de Bono J , et al . Cabins, castles, and constant hearts: rhythm control therapy in patients with atrial fibrillation. Eur Heart J 2019;40:3793–9. 10.1093/eurheartj/ehz782 31755940 PMC6898884

[6] Kirchhof P , Camm AJ , Goette A , et al . Early Rhythm-Control therapy in patients with atrial fibrillation. N Engl J Med 2020;383:1305–16. 10.1056/NEJMoa2019422 32865375

[7] Littlejohns TJ , Sudlow C , Allen NE , et al . Uk Biobank: opportunities for cardiovascular research. Eur Heart J 2019;40:1158–66. 10.1093/eurheartj/ehx254 28531320 PMC6451771

[8] Kirchhof P , Breithardt G , Camm AJ , et al . Improving outcomes in patients with atrial fibrillation: rationale and design of the early treatment of atrial fibrillation for stroke prevention trial. Am Heart J 2013;166:442–8. 10.1016/j.ahj.2013.05.015 24016492

[9] Kim D , Yang P-S , You SC , et al . Treatment timing and the effects of rhythm control strategy in patients with atrial fibrillation: nationwide cohort study. BMJ 2021;373:n991. 10.1136/bmj.n991 33975876 PMC8111568

[10] Distance M . The Concise encyclopedia of statistics. New York. NY: Springer New York, 2008: 325–6.

[11] Martin-Doyle W , Essebag V , Zimetbaum P , et al . Trends in US hospitalization rates and rhythm control therapies following publication of the Affirm and race trials. J Cardiovasc Electrophysiol 2011;22:548–53. 10.1111/j.1540-8167.2010.01950.x 21087329 PMC3060275

[12] Kirchhof P , Ammentorp B , Darius H , et al . Management of atrial fibrillation in seven European countries after the publication of the 2010 ESC Guidelines on atrial fibrillation: primary results of the PREvention oF thromboemolic events--European Registry in Atrial Fibrillation (PREFER in AF). Europace 2014;16:6–14. 10.1093/europace/eut263 24084680 PMC3864758

[13] Willems S , Borof K , Brandes A . Systematic, early rhythm control strategy for atrial fibrillation in patients with or without symptoms: the EAST-AFNET 4 trial. Eur Heart J 2021. 10.1093/eurheartj/ehab593 PMC8934687 34447995

[14] Phillips K , Subramanian A , Thomas GN , et al . Trends in the pharmacological management of atrial fibrillation in UK general practice 2008-2018. Heart 2022;108:517–22. 10.1136/heartjnl-2021-319338 34226195

[15] Andrade JG , Wells GA , Deyell MW , et al . Cryoablation or drug therapy for initial treatment of atrial fibrillation. N Engl J Med 2021;384:305–15. 10.1056/NEJMoa2029980 33197159

[16] Packer DL , Piccini JP , Monahan KH , et al . Ablation versus drug therapy for atrial fibrillation in heart failure: results from the CABANA trial. Circulation 2021;143:1377–90. 10.1161/CIRCULATIONAHA.120.050991 33554614 PMC8030730

[17] Rillig A , Magnussen C , Ozga A-K , et al . Early rhythm control therapy in patients with atrial fibrillation and heart failure. Circulation 2021;144:845–58. 10.1161/CIRCULATIONAHA.121.056323 34328366 PMC8456351

[18] Quan H , Eastwood C , Cunningham CT , et al . Validity of AHRQ patient safety indicators derived from ICD-10 hospital discharge Abstract data (chart review study). BMJ Open 2013;3:e003716. 10.1136/bmjopen-2013-003716 PMC3796280 24114372

[19] Dickow J , Kirchhof P , Van Houten HK , et al . Generalizability of the EAST-AFNET 4 trial: assessing outcomes of early Rhythm-Control therapy in patients with atrial fibrillation. J Am Heart Assoc 2022;11:e024214. 10.1161/JAHA.121.024214 35621202 PMC9238730

[20] Fry A , Littlejohns TJ , Sudlow C , et al . Comparison of sociodemographic and health-related characteristics of UK Biobank participants with those of the general population. Am J Epidemiol 2017;186:1026–34. 10.1093/aje/kwx246 28641372 PMC5860371

