# Supplementary material for: Eligibility for early rhythm control in patients with atrial fibrillation in the UK Biobank
Source: Heart. 2022 Jul 14;108(23):1873–80. doi: 10.1136/heartjnl-2022-321196 (PMC9664114; doi:10.1136/heartjnl-2022-321196)
Supplement: Supplementary data [file heartjnl-2022-321196supp002.pdf]

Read2	BNF Code	DMD code	Drug name
**Anticoagulation**			
	20802		Apixaban Tablets 2.5 mg
	20802		Apixaban Tablets 5 mg
			Apixaban Tablets 5 mg
	50.00.00.00.00		apixaban (form not specified)
bs72.	2080200		Apixaban 2.5mg tablets
bs72.		703907006	Apixaban 2.5mg tablets
bs72.00		703907006	Apixaban 2.5mg tablets
	02.08.02.00.00		Apixaban 2.5mg tablets
bs74.	2080200		Apixaban 5mg tablets
bs74.		703908001	Apixaban 5mg tablets
bs74.00		703908001	Apixaban 5mg tablets
	02.08.02.00.00		Apixaban 5mg tablets
	20802		Dabigatran Etexilate Capsules 110 mg
	20802		Dabigatran Etexilate Capsules 150 mg
	20802		Dabigatran Etexilate Capsules 75 mg
bs4y.	2080200		Dabigatran etexilate 110mg capsules
bs4y.00		13532811000001109	Dabigatran etexilate 110mg capsules
	02.08.02.00.00		Dabigatran etexilate 110mg capsules
bs4x.	2080200		Dabigatran etexilate 150mg capsules
bs4x.00		19469811000001101	Dabigatran etexilate 150mg capsules
	02.08.02.00.00		Dabigatran etexilate 150mg capsules
bs4z.00		13532911000001104	Dabigatran etexilate 75mg capsules
	02.08.02.00.00		Dabigatran etexilate 75mg capsules
bs85.00		29903311000001108	Edoxaban 30mg tablets
bs86.00		29903411000001101	Edoxaban 60mg tablets
	02.08.02.00.00		Edoxaban 60mg tablets
	20802		Rivaroxaban Tablets 10 mg
	20802		Rivaroxaban Tablets 15 mg
			Rivaroxaban Tablets 15 mg
	20802		Rivaroxaban Tablets 20 mg
			Rivaroxaban Tablets 20 mg
	50.00.00.00.00		rivaroxaban (form not specified)
bs6z.	2080200		Rivaroxaban 10mg tablets
bs6z.00		14254711000001104	Rivaroxaban 10mg tablets
	02.08.02.00.00		Rivaroxaban 10mg tablets
bs6y.	2080200		Rivaroxaban 15mg tablets
bs6y.		19842111000001101	Rivaroxaban 15mg tablets
bs6y.00		19842111000001101	Rivaroxaban 15mg tablets
	02.08.02.00.00		Rivaroxaban 15mg tablets

bs6w.	2080200		Rivaroxaban 2.5mg tablets
bs6w.00		27810711000001104	Rivaroxaban 2.5mg tablets
	02.08.02.00.00		Rivaroxaban 2.5mg tablets
bs6x.	2080200		Rivaroxaban 20mg tablets
bs6x.		19842211000001107	Rivaroxaban 20mg tablets
bs6x.00		19842211000001107	Rivaroxaban 20mg tablets
	02.08.02.00.00		Rivaroxaban 20mg tablets
bs6z.00		14254711000001104	RIVAROXABAN FC tab 10mg
		299275001000027104	WARFARIN
bs17.00		319733000	WARFARIN 1MG
		32095001000027107	Warfarin 1mg Tablet (WB Pharmaceuticals Ltd)
bs17.	2080200		Warfarin 1mg tablets
bs17.00		319733000	Warfarin 1mg tablets
bs17.00		319733000	WARFARIN 1mg tablets
	02.08.02.00.00		Warfarin 1mg tablets
		299275001000027104	WARFARIN 1mg tablets
	02.08.02.00.00		Warfarin 1mg tablets (A A H Pharmaceuticals Ltd)
	02.08.02.00.00		Warfarin 1mg tablets (Actavis UK Ltd)
	02.08.02.00.00		Warfarin 1mg tablets (Almus Pharmaceuticals Ltd)
	02.08.02.00.00		Warfarin 1mg tablets (Arrow Generics Ltd)
		2.72711E+14	Warfarin 1mg tablets (IVAX Pharmaceuticals UK Ltd)
	02.08.02.00.00		Warfarin 1mg tablets (Sandoz Ltd)
	02.08.02.00.00		Warfarin 1mg tablets (Teva UK Ltd)
	02.08.02.00.00		Warfarin 1mg/ml oral suspension sugar free
		32105001000027102	Warfarin 3mg Tablet (WB Pharmaceuticals Ltd)
bs18.	2080200		Warfarin 3mg tablets
bs18.		319734006	Warfarin 3mg tablets
bs18.		319734006	WARFARIN 3mg tablets
bs18.		319734006	WARFARIN 3MG TABLETS
bs18.00		319734006	Warfarin 3mg tablets
	02.08.02.00.00		Warfarin 3mg tablets
	02.08.02.00.00		Warfarin 3mg tablets (A A H Pharmaceuticals Ltd)
bs18.00		5.55911E+14	Warfarin 3mg tablets (Actavis UK Ltd)
	02.08.02.00.00		Warfarin 3mg tablets (Actavis UK Ltd)
	02.08.02.00.00		Warfarin 3mg tablets (Almus Pharmaceuticals Ltd)
	02.08.02.00.00		Warfarin 3mg tablets (Arrow Generics Ltd)
	02.08.02.00.00		Warfarin 3mg tablets (IVAX Pharmaceuticals UK Ltd)
		9.27211E+14	Warfarin 3mg tablets (IVAX Pharmaceuticals UK Ltd)
	02.08.02.00.00		Warfarin 3mg tablets (Sandoz Ltd)
	02.08.02.00.00		Warfarin 3mg tablets (Teva UK Ltd)
bs1..		8798511000001101	Warfarin 3mg/5ml oral solution

	02.08.02.00.00		Warfarin 3mg/5ml oral solution
bs1A.	2080200		Warfarin 500microgram tablets
bs1A.00		319736008	Warfarin 500microgram tablets
	02.08.02.00.00		Warfarin 500microgram tablets
	02.08.02.00.00		Warfarin 500microgram tablets (A A H Pharmaceuticals Ltd)
	02.08.02.00.00		Warfarin 500microgram tablets (Actavis UK Ltd)
bs19.00		319735007	WARFARIN 5MG
bs19.	2080200		Warfarin 5mg tablets
bs19.00		319735007	Warfarin 5mg tablets
bs19.00		319735007	WARFARIN 5mg tablets
	02.08.02.00.00		Warfarin 5mg tablets
	02.08.02.00.00		Warfarin 5mg tablets (A A H Pharmaceuticals Ltd)
	02.08.02.00.00		Warfarin 5mg tablets (Actavis UK Ltd)
	02.08.02.00.00		Warfarin 5mg tablets (Almus Pharmaceuticals Ltd)
	02.08.02.00.00		Warfarin 5mg tablets (Arrow Generics Ltd)
		7.29811E+14	Warfarin 5mg tablets (IVAX Pharmaceuticals UK Ltd)
	02.08.02.00.00		Warfarin 5mg tablets (Teva UK Ltd)
	02.08.02.00.00		Warfarin 5mg tablets (Zentiva)
bs1..		8798711000001106	Warfarin 5mg/5ml oral solution
bs17.00		319733000	WARFARIN SOD TAB 1MG
		299275001000027104	WARFARIN SOD TAB 1MG
bs18.		319734006	WARFARIN SOD TAB 3MG
		299275001000027104	WARFARIN SOD TAB 3MG
bs19.00		319735007	WARFARIN SOD TAB 5MG
		299275001000027104	WARFARIN SOD TAB 5MG
bs17.00		319733000	WARFARIN SODIUM
bs18.		319734006	WARFARIN SODIUM
		299275001000027104	WARFARIN SODIUM
	20802		Warfarin Sodium Oral solution 1 mg/5 ml
	20802		Warfarin Sodium Oral solution 3 mg/5 ml
bs17.00		319733000	Warfarin Sodium Tablets 1 mg
	0208020V0AAAAAA		Warfarin Sodium Tablets 1 mg
bs18.		319734006	Warfarin Sodium Tablets 3 mg
bs18.00		319734006	Warfarin Sodium Tablets 3 mg
	0208020V0AAABAB		Warfarin Sodium Tablets 3 mg
bs19.00		319735007	Warfarin Sodium Tablets 5 mg
	0208020V0AAADAD		Warfarin Sodium Tablets 5 mg
	20802		Warfarin Sodium Tablets 500 micrograms
bs17.00		319733000	Warfarin sodium 1 mg tablets
		299275001000027104	WARFARIN SODIUM 1MG
bs17.00		319733000	WARFARIN SODIUM 1mg tablets

		299275001000027104	WARFARIN SODIUM 1mg tablets
		299275001000027104	WARFARIN SODIUM 3MG TAB
bs18.		319734006	WARFARIN SODIUM 3mg tablets
		299275001000027104	WARFARIN SODIUM 3mg tablets
		299275001000027104	WARFARIN SODIUM 5mg tablets
bs1..		879851100001101	WARFARIN SODIUM oral liq 3mg/5ml
	02.08.02.00.00		warfarin sodium oral suspension 5mg/5ml
	02.08.02.00.00		warfarin sodium oral suspension sugar-free 1mg/ml
bs17.00		319733000	Warfarin Sodium Tablets 1 mg
		299275001000027104	warfarin sodium tablets 1 mg
bs17.00		319733000	warfarin sodium tablets 1mg
bs17.00		319733000	Warfarin sodium tablets 1mg
bs17.00		319733000	Warfarin Sodium Tablets 1mg
bs17.00		319733000	WARFARIN SODIUM tablets 1mg
	0208020V0AAAAAA		Warfarin Sodium TABLETS 1MG
	0208020V0AAAAAA		WARFARIN SODIUM TABLETS 1MG
bs18.		319734006	Warfarin Sodium Tablets 3 mg
bs18.		319734006	Warfarin sodium tablets 3mg
bs18.		319734006	WARFARIN SODIUM tablets 3mg
	0208020V0AAABAB		Warfarin Sodium TABLETS 3MG
	0208020V0AAABAB		WARFARIN SODIUM TABLETS 3MG
		299275001000027104	warfarin sodium tablets 3mg
bs19.00		319735007	Warfarin Sodium Tablets 5 mg
		299275001000027104	warfarin sodium tablets 5 mg
bs1A.00		319736008	warfarin sodium tablets 500 micrograms
	20802		WARFARIN SODIUM TABLETS 500MICROGRAMS
bs19.00		319735007	Warfarin sodium tablets 5mg
bs19.00		319735007	Warfarin Sodium Tablets 5mg
bs19.00		319735007	WARFARIN SODIUM tablets 5mg
	0208020V0AAADAD		Warfarin Sodium TABLETS 5MG
	0208020V0AAADAD		WARFARIN SODIUM TABLETS 5MG
		299275001000027104	WARFARIN SODIUM Tabs
bs17.00		319733000	WARFARIN SODIUM tabs 1 mg
		299275001000027104	WARFARIN SODIUM tabs 1 mg
bs17.	2080200		WARFARIN SODIUM tabs 1mg
bs17.00		319733000	WARFARIN SODIUM tabs 1mg
bs17.00		319733000	WARFARIN SODIUM TABS 1MG
	0208020V0AAAAAA		Warfarin Sodium TABS 1MG
		299275001000027104	WARFARIN SODIUM tabs 3 mg
bs18.	2080200		WARFARIN SODIUM tabs 3mg
bs18.		319734006	WARFARIN SODIUM tabs 3mg

bs18.		319734006	WARFARIN SODIUM TABS 3MG
bs18.00		319734006	WARFARIN SODIUM tabs 3mg
	0208020V0AAABAB		Warfarin Sodium TABS 3MG
		299275001000027104	WARFARIN SODIUM TABS 3MG
		299275001000027104	WARFARIN SODIUM tabs 5 mg
bs1A.	2080200		WARFARIN SODIUM tabs 500 micrograms
bs1A.00		319736008	WARFARIN SODIUM tabs 500 micrograms
	20802		Warfarin Sodium TABS 500MICROGRAMS
bs19.	2080200		WARFARIN SODIUM tabs 5mg
bs19.00		319735007	WARFARIN SODIUM tabs 5mg
bs19.00		319735007	WARFARIN SODIUM TABS 5MG
	0208020V0AAADAD		Warfarin Sodium TABS 5MG
		299275001000027104	WARFARIN SODIUM TABS 5MG
bs17.00		319733000	WARFARIN TAB 1
bs17.00		319733000	WARFARIN TAB 1mg
bs18.		319734006	WARFARIN TAB 3
bs18.		319734006	WARFARIN TAB 3mg
bs19.00		319735007	WARFARIN TAB 5
bs19.00		319735007	WARFARIN TAB 5mg
bs17.00		319733000	Warfarin Tablets 1 mg
bs17.00		319733000	Warfarin tablets 1mg
		32095001000027107	Warfarin tablets 1mg
	02.08.02.00.00		WARFARIN tablets 1mg [AMCO]
bs18.		319734006	Warfarin Tablets 3 mg
bs18.		319734006	Warfarin tablets 3mg
bs18.00		319734006	Warfarin tablets 3mg
		299275001000027104	Warfarin tablets 3mg
		32105001000027102	Warfarin tablets 3mg
bs19.00		319735007	Warfarin tablets 5mg
		299275001000027104	Warfarin tablets 5mg
		2.72711E+14	WARFARIN tabs 1mg [IVAX]
		9.27211E+14	WARFARIN tabs 3mg [IVAX]
		32105001000027102	WARFARIN tabs 3mg [WB]
		3659711000001104	WARFARIN tabs 500 micrograms [HILLCROSS]
bs14.00		36055001000027102	WARFARIN WBP 1mg tabs
bs16.00		36075001000027104	WARFARIN WBP 5mg tabs
bs17.00		319733000	WARFARIN WBP TABLETS 1 MG
	02.08.02.00.00		WARFARIN WBP tablets 1mg [BOEH I HSP]
bs18.		319734006	WARFARIN WBP TABLETS 3 MG
	02.08.02.00.00		WARFARIN WBP tablets 3mg [BOEH I HSP]
	02.08.02.00.00		WARFARIN WBP tablets 5mg [BOEH I HSP]

Heart failure			
	02.05.05.01.00		Acepril 12.5mg tablets (Bristol-Myers Squibb Pharmaceuticals Ltd)
	02.05.05.01.00		Acepril 25mg tablets (Bristol-Myers Squibb Pharmaceuticals Ltd)
	02.05.05.01.00		Acepril 50mg tablets (Bristol-Myers Squibb Pharmaceuticals Ltd)
	02.05.05.01.00		Acezide 25mg/50mg tablets (Bristol-Myers Squibb Pharmaceuticals Ltd)
	02.02.03.00.00		Aldactide 25 tablets (Pfizer Ltd)
	02.02.01.00.00		Aldactide 50 tablets (Pfizer Ltd)
	0202040G0BBABAB		ALDACTIDE 50-P42 tablet
	0202040G0BBABAB		ALDACTIDE 50-P42 TABLET
	02.02.03.00.00		Aldactone 100mg tablets (Pfizer Ltd)
	02.02.03.00.00		Aldactone 25mg tablets (Pfizer Ltd)
	02.02.03.00.00		Aldactone 50mg tablets (Pfizer Ltd)
bk78.	02050502		Amias 16mg tablets (Takeda UK Ltd)
	02.05.05.02.00		Amias 16mg tablets (Takeda UK Ltd)
	02.05.05.02.00		Amias 2mg tablets (Takeda UK Ltd)
	02.05.05.02.00		Amias 32mg tablets (Takeda UK Ltd)
	02.05.05.02.00		Amias 4mg tablets (Takeda UK Ltd)
bk77.	02050502		Amias 8mg tablets (Takeda UK Ltd)
	02.05.05.02.00		Amias 8mg tablets (Takeda UK Ltd)
	0205052C0BBABAB		Amias TABS 4MG
	02.02.03.00.00		Amil-Co 5mg/50mg tablets (IVAX Pharmaceuticals UK Ltd)
	0205052I0BBABAB		Aprovel Tablets 150 mg
	0205052I0BBAAAA		Aprovel Tablets 75 mg
	0205052I0BBABAB		APROVEL 150MG
bk55.	02050502		Aprovel 150mg tablets (Sanofi)
	02.05.05.02.00		Aprovel 150mg tablets (Sanofi)
bk56.	02050502		Aprovel 300mg tablets (Sanofi)
	02.05.05.02.00		Aprovel 300mg tablets (Sanofi)
bk54.	02050502		Aprovel 75mg tablets (Sanofi)
	02.05.05.02.00		Aprovel 75mg tablets (Sanofi)
	0202020D0AAAEAE		Bumetanide Tablets 1 mg
	0202020D0AAAFAF		Bumetanide Tablets 5 mg
b32u.	02020200		Bumetanide 1mg tablets
	02.02.02.00.00		Bumetanide 1mg tablets
	02.02.02.00.00		Bumetanide 1mg tablets (Actavis UK Ltd)
	02.02.02.00.00		Bumetanide 1mg tablets (Almus Pharmaceuticals Ltd)
	02.02.02.00.00		Bumetanide 1mg tablets (Mylan Ltd)
	02.02.02.00.00		Bumetanide 1mg tablets (Niche Generics Ltd)
	02.02.02.00.00		Bumetanide 1mg tablets (Teva UK Ltd)

	02.02.02.00.00		Bumetanide 1mg/5ml oral solution sugar free
	02.02.08.00.00		Bumetanide 500microgram / Potassium chloride 573mg (potassium 7.7mmol) modified-release tablets
b32v.	02020200		Bumetanide 5mg tablets
	02.02.02.00.00		Bumetanide 5mg tablets
	020208		Bumetanide And Slow Potassium BP Tablets 500 micrograms + 7.7 mmol
	0202020D0AAAEAE		BUMETANIDE TABLETS 1MG
	0202020D0AAAEAE		Bumetanide TABS 1MG
	0202020D0AAAFAF		Bumetanide TABS 5MG
	02.02.02.00.00		Burinex 1mg tablets (LEO Pharma)
	02.02.02.00.00		Burinex 5mg tablets (LEO Pharma)
	02.02.02.00.00		Burinex A 5mg/1mg tablets (LEO Pharma)
	0202080C0BBAAAA		Burinex K Tablets
	02.02.08.00.00		Burinex K modified-release tablets (LEO Pharma)
	0202080C0BBAAAA		BURINEX K TABLETS
	0202080C0BBAAAA		Burinex K TABS
	0202080C0BBAAAA		Burinex k-P42 tablet
	0202080C0BBAAAA		BURINEX K-P42 TABLET
	0202080C0BBAAAA		BURINEX K-P42 tabs
	02.02.02.00.00		BURINEX oral liquid sugar-free 1mg/5ml [LEO]
bk74.	02050502		Candesartan 16mg tablets
	02.05.05.02.00		Candesartan 16mg tablets
	02.05.05.02.00		Candesartan 16mg tablets (Actavis UK Ltd)
	02.05.05.02.00		Candesartan 16mg tablets (Teva UK Ltd)
bk71.	02050502		Candesartan 2mg tablets
	02.05.05.02.00		Candesartan 2mg tablets
	02.05.05.02.00		Candesartan 2mg tablets (Teva UK Ltd)
bk7z.	02050502		Candesartan 32mg tablets
	02.05.05.02.00		Candesartan 32mg tablets
	02.05.05.02.00		Candesartan 32mg tablets (Actavis UK Ltd)
	02.05.05.02.00		Candesartan 32mg tablets (Teva UK Ltd)
bk72.	02050502		Candesartan 4mg tablets
	02.05.05.02.00		Candesartan 4mg tablets
	02.05.05.02.00		Candesartan 4mg tablets (Actavis UK Ltd)
	02.05.05.02.00		Candesartan 4mg tablets (Teva UK Ltd)
bk73.	02050502		Candesartan 8mg tablets
	02.05.05.02.00		Candesartan 8mg tablets
	02.05.05.02.00		Candesartan 8mg tablets (Actavis UK Ltd)
	02.05.05.02.00		Candesartan 8mg tablets (Teva UK Ltd)
	0205052C0AAADAD		Candesartan Cilexetil Tablets 16 mg
	0205052C0AAAAAA		Candesartan Cilexetil Tablets 2 mg

	02050502		Candesartan Cilexetil Tablets 32 mg
	0205052C0AAABAB		Candesartan Cilexetil Tablets 4 mg
	0205052C0AAACAC		Candesartan Cilexetil Tablets 8 mg
bk74.	02050502		Candesartan Cilexetil Tablets 16mg
	0205052C0AAADAD		Candesartan Cilexetil TABLETS 16MG
	0205052C0AAADAD		CANDESARTAN CILEXETIL TABLETS 16MG
	0205052C0AAAAAA		Candesartan Cilexetil TABLETS 2MG
	0205052C0AAAAAA		CANDESARTAN CILEXETIL TABLETS 2MG
	02050502		CANDESARTAN CILEXETIL TABLETS 32MG
	0205052C0AAABAB		Candesartan Cilexetil TABLETS 4MG
	0205052C0AAABAB		CANDESARTAN CILEXETIL TABLETS 4MG
	0205052C0AAACAC		CANDESARTAN CILEXETIL TABLETS 8MG
	0205052C0AAADAD		Candesartan Cilexetil TABS 16MG
	0205052C0AAAAAA		Candesartan Cilexetil TABS 2MG
	02050502		Candesartan Cilexetil TABS 32MG
	0205052C0AAABAB		Candesartan Cilexetil TABS 4MG
	0205052C0AAACAC		Candesartan Cilexetil TABS 8MG
	0205052C0AAABAB		CANDESARTAN TABLETS 4MG
bk74.	02050502		CANDESARTAN tabs 16mg
bk71.	02050502		CANDESARTAN tabs 2mg
bk7z.	02050502		CANDESARTAN tabs 32mg
bk72.	02050502		CANDESARTAN tabs 4mg
bk73.	02050502		CANDESARTAN tabs 8mg
	02.05.05.02.00		candesartan with hydrochlorothiazide (roi) tablets 16mg + 12.5mg
	02.05.05.01.00		Capoten 12.5mg tablets (Bristol-Myers Squibb Pharmaceuticals Ltd)
	02.05.05.01.00		Capoten 25mg tablets (Bristol-Myers Squibb Pharmaceuticals Ltd)
	02.05.05.01.00		Capoten 50mg tablets (Bristol-Myers Squibb Pharmaceuticals Ltd)
	0205051F0BCABAE		CAPOTEN-P42 25MG TABLET
bi1s.	02050501		Capozide 25mg/50mg tablets (Bristol-Myers Squibb Pharmace...
	02.02.01.00.00		Capozide 25mg/50mg tablets (Bristol-Myers Squibb Pharmaceuticals Ltd)
	02.05.05.01.00		Capozide LS 12.5mg/25mg tablets (Bristol-Myers Squibb Pharmaceuticals Ltd)
	02.05.05.01.00		CAPOZIDE LS tablets [SQUIBB]
	0205051F0AAADAD		Captopril Tablets 12.5 mg
	0205051F0AAAEAE		Captopril Tablets 25 mg
	0205051F0AAAFAF		Captopril Tablets 50 mg
bi1v.	02050501		Captopril 12.5mg tablets
	02.05.05.01.00		Captopril 12.5mg tablets

bi1x.	02050501		Captopril 25mg tablets
	02.05.05.01.00		Captopril 25mg tablets
bi1z.	02050501		Captopril 50mg tablets
	02.05.05.01.00		Captopril 50mg tablets
	0205051F0AAADAD		CAPTOPRIL TABLETS 12.5MG
	0205051F0AAAEAE		CAPTOPRIL TABLETS 25MG
	0205051F0AAAFAF		CAPTOPRIL TABLETS 50MG
	0205051F0AAADAD		Captopril TABS 12.5MG
	0205051F0AAAEAE		Captopril TABS 25MG
	0205051F0AAAFAF		Captopril TABS 50MG
	0205051F0AAAEAE		CAPTOPRIL-P42 25MG TABLET
	0205051K0BCABAB		CARACE 10 PLUS TABLETS
	02.02.01.00.00		CARACE 10 plus tablets [BMS]
	02.05.05.01.00		Carace 10mg tablets (Bristol-Myers Squibb Pharmaceuticals Ltd)
	02.05.05.01.00		Carace 2.5mg tablets (Bristol-Myers Squibb Pharmaceuticals Ltd)
	0205051K0BCAAAA		CARACE 20 PLUS TABLETS
	02.02.01.00.00		Carace 20 Plus tablets (Merck Sharp & Dohme Ltd)
	02.02.01.00.00		CARACE 20 plus tablets [BMS]
	02.05.05.01.00		Carace 20mg tablets (Bristol-Myers Squibb Pharmaceuticals Ltd)
	02.05.05.01.00		Carace 5mg tablets (Bristol-Myers Squibb Pharmaceuticals Ltd)
	0205051L0BCACAC		Carace TABS 10MG
	0205051L0BCABAB		Carace TABS 5MG
	0205051L0BCABAB		Carace-P42 5mg tablet
	0202010D0AAAFAF		Chlorothiazide TABS 500MG
	020201		Chlortalidone Tablets 50 mg
b23y.	02020100		Chlortalidone 50mg tablets
	02.02.01.00.00		Chlortalidone 50mg tablets
	02.02.01.00.00		Chlortalidone 50mg tablets (Sovereign Medical Ltd)
	020201		CHLORTALIDONE TABLETS 50MG
	020201		Chlortalidone TABS 50MG
	02.05.05.01.00		Cilazapril 1mg tablets
	02.05.05.01.00		Cilazapril 2.5mg tablets
	02.05.05.01.00		Cilazapril 500microgram tablets
	02.05.05.01.00		Cilazapril 5mg tablets
	0202040B0AAACAC		Co-Amilofruse 10/80 Tablets
b51z.	02020400		Co-amilofruse 10mg/80mg tablets
	02.02.04.00.00		Co-amilofruse 10mg/80mg tablets
	0202040B0AAACAC		Co-Amilofruse 10mg/80mg TABS
	0202040B0AAABAB		Co-Amilofruse 2.5/20 Tablets

b51x.	02020400		Co-amilofruse 2.5mg/20mg tablets
	02.02.02.00.00		Co-amilofruse 2.5mg/20mg tablets
	0202040B0AAABAB		Co-Amilofruse 2.5mg/20mg TABS
	0202040B0AAAAAA		Co-Amilofruse 5/40 Tablets
b51y.	02020400		Co-amilofruse 5mg/40mg tablets
	02.02.04.00.00		Co-amilofruse 5mg/40mg tablets
	0202040B0AAAAAA		CO-AMILOFRUSE 5MG/40MG TABLETS
	02.02.03.00.00		Co-amilofruse 5mg/40mg tablets (Actavis UK Ltd)
	02.02.02.00.00		Co-amilofruse 5mg/40mg tablets (Mylan Ltd)
	02.02.03.00.00		Co-amilofruse 5mg/40mg tablets (Teva UK Ltd)
	02.02.02.00.00		Co-amilofruse 5mg/40mg tablets (Wockhardt UK Ltd)
	0202040B0AAAAAA		Co-Amilofruse 5mg/40mg TABS
	02050502		Coaprovel Tablets 300 mg + 25 mg
	02050502		COAPROVEL 150MG/12.5MG TABLETS
bk57.	02050504		CoAprovel 150mg/12.5mg tablets (Sanofi)
	02.05.05.02.00		CoAprovel 150mg/12.5mg tablets (Sanofi)
bk58.	02050504		CoAprovel 300mg/12.5mg tablets (Sanofi)
	02.05.05.02.00		CoAprovel 300mg/12.5mg tablets (Sanofi)
bk59.	02050504		CoAprovel 300mg/25mg tablets (Sanofi)
	02.02.01.00.00		CoAprovel 300mg/25mg tablets (Sanofi)
	02050502		Coaprovel 300mg/25mg TABS
	02050502		Co-Diovan Tablets 160 mg/12.5 mg
	02050502		Co-Diovan Tablets 80 mg/12.5 mg
	02050502		CO-DIOVAN 160MG/12.5MG TABLETS
bk47.	02050502		Co-Diovan 160mg/12.5mg tablets (Novartis Pharmaceuticals ...
bk47.	02050504		Co-Diovan 160mg/12.5mg tablets (Novartis Pharmaceuticals ...
	02.02.01.00.00		Co-Diovan 160mg/12.5mg tablets (Novartis Pharmaceuticals UK Ltd)
	02.02.01.00.00		Co-Diovan 160mg/25mg tablets (Novartis Pharmaceuticals UK Ltd)
bk49.	02050504		Co-Diovan 80mg/12.5mg tablets (Novartis Pharmaceuticals U...
	02.02.01.00.00		Co-Diovan 80mg/12.5mg tablets (Novartis Pharmaceuticals UK Ltd)
	02050502		CO-DIOVAN TABLETS 80/12.5MG
	02.02.04.00.00		Co-flumactone 25mg/25mg tablets
	0202040G0AAAAAA		Co-Flumactone 25mg/25mg TABS
	0202040G0AAABAB		Co-Flumactone 50/50 Tablets
	02.02.03.00.00		Co-flumactone 50mg/50mg tablets
	0202040G0AAABAB		Co-Flumactone 50mg/50mg TABS
	02.02.01.00.00		Co-tenidone 50mg/12.5mg tablets
	02.02.01.00.00		Co-tenidone 50mg/12.5mg tablets (Actavis UK Ltd)

	02.05.05.01.00		Coversyl 2mg tablets (Servier Laboratories Ltd)
	02.05.05.01.00		Coversyl 4mg tablets (Servier Laboratories Ltd)
	02.05.05.01.00		Coversyl 8mg tablets (Servier Laboratories Ltd)
	02.05.05.01.00		Coversyl Arginine 10mg tablets (Servier Laboratories Ltd)
	02.05.05.01.00		Coversyl Arginine 2.5mg tablets (Servier Laboratories Ltd)
	02.05.05.01.00		Coversyl Arginine 5mg tablets (Servier Laboratories Ltd)
	02050501		Coversyl Arginine Plus Tablets 5 mg + 1.25 mg
	02.05.05.01.00		Coversyl Arginine Plus 5mg/1.25mg tablets (Servier Laboratories Ltd)
	02050501		Coversyl Plus Tablets
	02.05.05.01.00		Coversyl Plus tablets (Servier Laboratories Ltd)
	02050501		Coversyl Plus TABS
	02050501		Coversyl TABS 4MG
	02050502		Cozaar Tablets 100 mg
	0205052N0BBABAB		Cozaar Tablets 50 mg
bk38.	02050502		Cozaar 100mg tablets (Merck Sharp & Dohme Ltd)
	02.05.05.02.00		Cozaar 100mg tablets (Merck Sharp & Dohme Ltd)
	02.05.05.02.00		Cozaar 12.5mg tablets (Merck Sharp & Dohme Ltd)
	02.05.05.02.00		Cozaar 25mg tablets (Merck Sharp & Dohme Ltd)
	0205052N0BBABAB		cozaar 50mg tablet
	02.05.05.02.00		Cozaar 50mg tablets (Merck Sharp & Dohme Ltd)
	02050502		Cozaar Comp 100mg/12.5mg TABS
	02050502		Cozaar Comp 100mg/25mg TABS
	0205052P0BBAAAA		COZAAR COMP 50MG/12.5MG TABLETS
	0205052P0BBAAAA		Cozaar Comp 50mg/12.5mg TABS
	02050502		COZAAR TABLETS 100MG
	02050502		Cozaar TABS 100MG
	0205052N0BBABAB		Cozaar TABS 50MG
	0205052P0BBAAAA		Cozaar-Comp Tablets
	02050502		Cozaar-Comp Tablets 100 mg + 25 mg
	0205052P0BBAAAA		Cozaar-Comp Tablets 50 mg + 12.5 mg
	02.05.05.02.00		Cozaar-Comp 100mg/12.5mg tablets (Merck Sharp & Dohme Ltd)
	02.02.01.00.00		Cozaar-Comp 100mg/25mg tablets (Merck Sharp & Dohme Ltd)
bk36.	02050504		Cozaar-Comp 50mg/12.5mg tablets (Merck Sharp & Dohme Ltd)
	02.02.01.00.00		Cozaar-Comp 50mg/12.5mg tablets (Merck Sharp & Dohme Ltd)
	02.05.05.01.00		Co-zidocapt 12.5mg/25mg tablets
	02.02.01.00.00		Co-zidocapt 25mg/50mg tablets
	0205052V0BBACAC		Diovan Capsules 160 mg
	0205052V0BBAAAA		Diovan Capsules 40 mg

	0205052V0BBABAB		Diovan Capsules 80 mg
	02.05.05.02.00		Diovan 160mg capsules (Novartis Pharmaceuticals UK Ltd)
bk44.	02050502		Diovan 40mg capsules (Novartis Pharmaceuticals UK Ltd)
	02.05.05.02.00		Diovan 40mg capsules (Novartis Pharmaceuticals UK Ltd)
	02.05.05.02.00		Diovan 40mg tablets (Novartis Pharmaceuticals UK Ltd)
bk45.	02050502		Diovan 80mg capsules (Novartis Pharmaceuticals UK Ltd)
	02.05.05.02.00		Diovan 80mg capsules (Novartis Pharmaceuticals UK Ltd)
	0205052V0BBAAAA		Diovan CAPS 40MG
	0202080K0BCAAAB		DIUMIDE K-P42 TABLET
	02.02.08.00.00		Diumide-K Continus tablets (Teofarma)
	02.02.01.00.00		Diurexan 20mg tablets (Meda Pharmaceuticals Ltd)
	0202010Y0BBAAAA		DIUREXAN TABLETS 20MG
	0202010Y0BBAAAA		Diurexan TABS 20MG
bi2x.	02050501		Enalapril 10mg tablets
	02.05.05.01.00		Enalapril 10mg tablets
	02.05.05.01.00		Enalapril 10mg tablets (A A H Pharmaceuticals Ltd)
	02.05.05.01.00		Enalapril 10mg tablets (Almus Pharmaceuticals Ltd)
	02.05.05.01.00		Enalapril 10mg tablets (Teva UK Ltd)
bi2t.	02050501		Enalapril 2.5mg tablets
	02.05.05.01.00		Enalapril 2.5mg tablets
	02.05.05.01.00		Enalapril 2.5mg tablets (Dexcel-Pharma Ltd)
bi2b.	02050501		Enalapril 20mg / Hydrochlorothiazide 12.5mg tablets
	02.05.05.01.00		Enalapril 20mg / Hydrochlorothiazide 12.5mg tablets
bi2y.	02050501		Enalapril 20mg tablets
bi2z.	02050501		Enalapril 20mg tablets
	02.05.05.01.00		Enalapril 20mg tablets
	02.05.05.01.00		Enalapril 20mg tablets (Almus Pharmaceuticals Ltd)
	02.05.05.01.00		Enalapril 20mg tablets (Teva UK Ltd)
bi2u.	02050501		Enalapril 5mg tablets
bi2v.	02050501		Enalapril 5mg tablets
	02.05.05.01.00		Enalapril 5mg tablets
	02.05.05.01.00		Enalapril 5mg tablets (Kent Pharmaceuticals Ltd)
	02.05.05.01.00		Enalapril 5mg tablets (Teva UK Ltd)
	02050501		Enalapril And Hydrochlorothiazide Tablets 20 mg + 12.5 mg
	0205051I0AAACAC		Enalapril Maleate Tablets 10 mg
	0205051I0AAAAAA		Enalapril Maleate Tablets 2.5 mg
	0205051I0AAADAD		Enalapril Maleate Tablets 20 mg
	0205051I0AAABAB		Enalapril Maleate Tablets 5 mg
	0205051I0AAABAB		ENALAPRIL MALEATE 5MG TABLETS
	0205051I0AAADAD		ENALAPRIL MALEATE NON TARIFF TABLETS 20MG
	0205051I0AAADAD		Enalapril Maleate Non Tariff TABS 20MG

	0205051I0AAACAC		Enalapril Maleate TABLETS 10MG
	0205051I0AAACAC		ENALAPRIL MALEATE TABLETS 10MG
	0205051I0AAACAC		ENALAPRIL MALEATE TABLETS 10MG-P42 0
	0205051I0AAAAAA		Enalapril Maleate TABLETS 2.5MG
	0205051I0AAAAAA		ENALAPRIL MALEATE TABLETS 2.5MG
	0205051I0AAADAD		ENALAPRIL MALEATE TABLETS 20MG
	0205051I0AAABAB		Enalapril Maleate TABLETS 5MG
	0205051I0AAABAB		ENALAPRIL MALEATE TABLETS 5MG
	0205051I0AAABAB		ENALAPRIL MALEATE TABLETS 5MG-P42 0
	0205051I0AAACAC		Enalapril Maleate TABS 10MG
	0205051I0AAAAAA		Enalapril Maleate TABS 2.5MG
	0205051I0AAADAD		Enalapril Maleate TABS 20MG
	0205051I0AAABAB		Enalapril Maleate TABS 5MG
	0205051I0AAABAB		ENALAPRIL MALEATE tabs 5mg-P42 0
	0205051I0AAACAC		Enalapril maleate-P42 10mg tab
	0205051I0AAADAD		ENALAPRIL MALEATE-P42 20mg tab
	0205051I0AAABAB		ENALAPRIL MALEATE-P42 5MG TAB
	0205051I0AAACAC		ENALAPRIL TABLETS 10MG
	0205051I0AAACAC		ENALAPRIL TABLETS 10MG-P42 0
	0205051I0AAABAB		ENALAPRIL TABLETS 5MG
	0205051I0AAABAB		ENALAPRIL TABLETS 5MG-P42 0
	0205051I0AAACAC		ENALAPRIL tablets TAB 10mg-P42 0
	0205051I0AAABAB		ENALAPRIL tablets TAB 5mg-P42 0
bi2z.	02050501		ENALAPRIL tabs 20mg
bi2v.	02050501		ENALAPRIL tabs 5mg
	02.05.05.01.00		enalapril wafer 20mg
bkL1.	02050502		Entresto 24mg/26mg tablets (Novartis Pharmaceuticals UK Ltd)
	020203		Eplerenone Tablets 25 mg
	020203		Eplerenone Tablets 50 mg
b45z.	02020300		Eplerenone 25mg tablets
	02.02.03.00.00		Eplerenone 25mg tablets
	02.02.03.00.00		Eplerenone 25mg tablets (Actavis UK Ltd)
	02.02.03.00.00		Eplerenone 25mg tablets (Teva UK Ltd)
b45y.	02020300		Eplerenone 50mg tablets
	02.02.03.00.00		Eplerenone 50mg tablets
	020203		EPLERENONE TABLETS 25MG
b45z.	02020300		EPLERENONE tabs 25mg
	020203		Eplerenone TABS 25MG
	020203		Eplerenone TABS 50MG
	02050502		Eprosartan Tablets 300 mg
	02050502		Eprosartan Tablets 400 mg

	02050502		Eprosartan Tablets 600 mg
bk9x.	02050502		Eprosartan 300mg tablets
	02.05.05.02.00		Eprosartan 300mg tablets
bk9y.	02050502		Eprosartan 400mg tablets
	02.05.05.02.00		Eprosartan 400mg tablets
	02.05.05.02.00		Eprosartan 600mg tablets
	02050502		EPROSARTAN MESILATE TABLETS 300MG
	02050502		EPROSARTAN MESILATE TABLETS 400MG
	02050502		EPROSARTAN MESILATE TABLETS 600MG
	02050502		Eprosartan Mesilate TABS 600MG
	02.05.05.02.00		Exforge 10mg/160mg tablets (Novartis Pharmaceuticals UK Ltd)
	02.05.05.02.00		Exforge 5mg/160mg tablets (Novartis Pharmaceuticals UK Ltd)
	02.05.05.02.00		Exforge 5mg/80mg tablets (Novartis Pharmaceuticals UK Ltd)
	02.05.05.01.00		Fosinopril 10mg tablets
	02.05.05.01.00		Fosinopril 10mg tablets (Actavis UK Ltd)
bi72.	02050501		Fosinopril 20mg tablets
	02.05.05.01.00		Fosinopril 20mg tablets
	02.05.05.01.00		Fosinopril 20mg tablets (A A H Pharmaceuticals Ltd)
	0205051J0AAAAAA		Fosinopril Sodium Tablets 10 mg
	0205051J0AAABAB		Fosinopril Sodium Tablets 20 mg
	0205051J0AAAAAA		FOSINOPRIL SODIUM TABLETS 10MG
	0205051J0AAABAB		FOSINOPRIL SODIUM TABLETS 20MG
	0205051J0AAAAAA		Fosinopril Sodium TABS 10MG
	0205051J0AAABAB		Fosinopril Sodium TABS 20MG
	0205051J0AAABAB		FOSINOPRIL TABLETS 20MG
	0202040B0BBAAAA		Frumil Tablets
	02.02.03.00.00		Frumil 40mg/5mg tablets (Sanofi)
	0202040B0BBACAC		Frumil Forte Tablets
	02.02.04.00.00		Frumil Forte 10mg/80mg tablets (Sanofi)
	0202040B0BBACAC		Frumil Forte TABS
	02.02.02.00.00		Frumil LS 20mg/2.5mg tablets (Sanofi)
	0202040B0BBABAB		FRUMIL LS TABLETS
	02.02.02.00.00		FRUMIL LS tablets 20mg + 2.5mg [HELIOS]
	0202040B0BBABAB		Frumil Ls TABS
	0202040B0BBAAAA		FRUMIL TABLETS
	02.02.04.00.00		FRUMIL tablets 40mg + 5mg [HELIOS]
	0202040B0BBAAAA		Frumil TABS
	0202040B0BBAAAA		FRUMIL TABS
	0202040B0BBAAAA		Frumil-P42 tablet

	0202020L0AABBBB		Frusemide Tablets 20 mg
	0202020L0AABDBD		Frusemide Tablets 40 mg
	020202		Frusemide-P42 20mg tablet
	020202		FRUSEMIDE-P42 20mg tablet
	020202		FRUSEMIDE-P42 40mg tabs
	0202040U0BBAAAA		Frusene Tablets
b517.	02020400		Frusene 50mg/40mg tablets (Orion Pharma (UK) Ltd)
	02.02.04.00.00		Frusene 50mg/40mg tablets (Orion Pharma (UK) Ltd)
	0202040U0BBAAAA		FRUSENE TABLETS
	0202040U0BBAAAA		FRUSENE-P42 TABLET
	020202		Frusol Solution 40 mg/5 ml
	02.02.02.00.00		Frusol 40mg/5ml oral solution (Rosemont Pharmaceuticals Ltd)
	020202		Furosemide Tablets 20 mg
	020202		Furosemide Tablets 40 mg
	020202		Furosemide Tablets 500 mg
b311.	02020200		Furosemide 20mg tablets
	02.02.02.00.00		Furosemide 20mg tablets
	02.02.02.00.00		Furosemide 20mg tablets (A A H Pharmaceuticals Ltd)
	02.02.02.00.00		Furosemide 20mg tablets (Actavis UK Ltd)
	02.02.02.00.00		Furosemide 20mg tablets (Almus Pharmaceuticals Ltd)
	02.02.02.00.00		Furosemide 20mg tablets (Ranbaxy (UK) Ltd)
	02.02.02.00.00		Furosemide 20mg tablets (Teva UK Ltd)
	02.02.02.00.00		Furosemide 20mg/2ml solution for injection ampoules
b31r.	02020200		Furosemide 20mg/5ml oral solution sugar free
	02.02.02.00.00		Furosemide 20mg/5ml oral solution sugar free
	02.02.02.00.00		Furosemide 20mg/5ml oral solution sugar free (Focus Pharmaceuticals Ltd)
b312.	02020200		Furosemide 40mg tablets
	02.02.02.00.00		Furosemide 40mg tablets
	02.02.02.00.00		Furosemide 40mg tablets (A A H Pharmaceuticals Ltd)
	02.02.02.00.00		Furosemide 40mg tablets (Actavis UK Ltd)
	02.02.02.00.00		Furosemide 40mg tablets (Almus Pharmaceuticals Ltd)
	02.02.02.00.00		Furosemide 40mg tablets (Ranbaxy (UK) Ltd)
	02.02.02.00.00		Furosemide 40mg tablets (Sandoz Ltd)
	02.02.02.00.00		Furosemide 40mg tablets (Teva UK Ltd)
b31s.	02020200		Furosemide 40mg/5ml oral solution sugar free
	02.02.02.00.00		Furosemide 40mg/5ml oral solution sugar free
	02.02.02.00.00		Furosemide 500mg tablets
	02.02.02.00.00		Furosemide 500mg tablets (Actavis UK Ltd)
	02.02.02.00.00		Furosemide 50mg/5ml oral solution sugar free
b31u.	02020200		Furosemide 50mg/5ml solution for injection ampoules

	02.02.02.00.00		Furosemide 50mg/5ml solution for injection ampoules
	02.02.02.00.00		Furosemide 80mg/8ml solution for injection pre-filled syringes
	020202		Furosemide Sf Oral SOLN 40MG/5ML
	020202		Furosemide TABLETS 20MG
	020202		FUROSEMIDE TABLETS 20MG
	020202		Furosemide TABLETS 40MG
	020202		FUROSEMIDE TABLETS 40MG
	02.02.02.00.00		FUROSEMIDE tablets 40mg [CP PHARM]
b311.	02020200		FUROSEMIDE tabs 20mg
	020202		Furosemide TABS 20MG
b312.	02020200		FUROSEMIDE tabs 40mg
	020202		Furosemide TABS 40MG
	020202		Furosemide TABS 500MG
	02.02.04.00.00		furosemide with amiloride tablets 20mg + 2.5mg
bi95.	02050501		Gopten 1mg capsules (Abbott Laboratories Ltd)
	02.05.05.01.00		Gopten 1mg capsules (Abbott Laboratories Ltd)
	02.05.05.01.00		Gopten 2mg capsules (Abbott Laboratories Ltd)
	02.05.05.01.00		Gopten 4mg capsules (Abbott Laboratories Ltd)
	02.05.05.01.00		Gopten 500microgram capsules (Abbott Laboratories Ltd)
	0205051U0BBAAAA		Gopten CAPS 500MICROGRAMS
	02.02.01.00.00		Hygroton 50mg tablets (Alliance Pharmaceuticals Ltd)
	02.02.01.00.00		HYGROTON -K tablets [NOV/GEIGY]
	0202010F0BBAAAA		HYGROTON TABLETS 50MG
	0202010F0BBAAAA		Hygroton TABS 50MG
	02.05.05.01.00		HYTENEZE tablets 25mg [OPUS]
	02.05.05.01.00		HYTENEZE tablets 50mg [OPUS]
biBz.	02050501		Imidapril 10mg tablets
	02.05.05.01.00		Imidapril 10mg tablets
	02.05.05.01.00		Imidapril 20mg tablets
	02.05.05.01.00		Imidapril 5mg tablets
	02050501		IMIDAPRIL HYDROCHLORIDE TABLETS 10MG
	02050501		IMIDAPRIL HYDROCHLORIDE TABLETS 5MG
	02050501		Imidapril Hydrochloride TABS 10MG
	0202010P0AAADAD		Indapamide M/R tablets 1.5 mg
	0202010P0AAAAAA		Indapamide Tablets 2.5 mg
b285.	02020100		Indapamide 1.5mg modified-release tablets
	02.02.01.00.00		Indapamide 1.5mg modified-release tablets
	0202010P0AAADAD		Indapamide 1.5mg modified-release tablets
	02.02.01.00.00		Indapamide 1.5mg modified-release tablets (A A H Pharmaceuticals Ltd)
	02.02.01.00.00		Indapamide 1.5mg modified-release tablets (Sandoz Ltd)

b28z.	02020100		Indapamide 2.5mg tablets
	02.02.01.00.00		Indapamide 2.5mg tablets
	02.02.01.00.00		Indapamide 2.5mg tablets (A A H Pharmaceuticals Ltd)
	02.02.01.00.00		Indapamide 2.5mg tablets (Actavis UK Ltd)
	02.02.01.00.00		Indapamide 2.5mg tablets (Arrow Generics Ltd)
	02.02.01.00.00		Indapamide 2.5mg tablets (Mylan Ltd)
	02.02.01.00.00		Indapamide 2.5mg tablets (Niche Generics Ltd)
	02.02.01.00.00		Indapamide 2.5mg tablets (Teva UK Ltd)
	02.02.01.00.00		Indapamide 2.5mg tablets (Zentiva)
	0202010P0AAADAD		Indapamide Mr TABLETS 1.5MG
	0202010P0AAADAD		INDAPAMIDE MR TABLETS 1.5MG
	0202010P0AAADAD		Indapamide Mr TABS 1.5MG
	0202010P0AAAAAA		INDAPAMIDE TABLETS 2.5MG
	0202010P0AAAAAA		Indapamide TABS 2.5MG
	0205051I0BBACAC		Innovace Tablets 10 mg
	0205051I0BBADAD		Innovace Tablets 20 mg
	0205051I0BBABAB		Innovace Tablets 5 mg
bi24.	02050501		Innovace 10mg tablets (Merck Sharp & Dohme Ltd)
	02.05.05.01.00		Innovace 10mg tablets (Merck Sharp & Dohme Ltd)
	02.05.05.01.00		Innovace 2.5mg tablets (Merck Sharp & Dohme Ltd)
	02.05.05.01.00		Innovace 20mg tablets (Merck Sharp & Dohme Ltd)
	02.05.05.01.00		Innovace 5mg tablets (Merck Sharp & Dohme Ltd)
	02.05.05.01.00		INNOVACE MELT wafer 10mg [M S D]
	02.05.05.01.00		INNOVACE MELT wafer 5mg [M S D]
	0205051I0BBACAC		INNOVACE TABLETS 10MG
	0205051I0BBABAB		INNOVACE TABLETS 5MG
	0205051I0BBACAC		Innovace TABS 10MG
	0205051I0BBADAD		Innovace TABS 20MG
bi28.	02050501		Innozide 20mg/12.5mg tablets (Merck Sharp & Dohme Ltd)
	02.05.05.01.00		Innozide 20mg/12.5mg tablets (Merck Sharp & Dohme Ltd)
	0205051H0BBAAAA		Innozide TABS
	02.02.03.00.00		Inspra 25mg tablets (Pfizer Ltd)
	0205052I0AAABAB		Irbesartan Tablets 150 mg
	0205052I0AAACAC		Irbesartan Tablets 300 mg
	0205052I0AAAAAA		Irbesartan Tablets 75 mg
	02.02.01.00.00		Irbesartan 150mg / Hydrochlorothiazide 12.5mg tablets
bk52.	02050502		Irbesartan 150mg tablets
	02.05.05.02.00		Irbesartan 150mg tablets
	0205052I0AAABAB		IRBESARTAN 150MG TABLETS
	02.05.05.02.00		Irbesartan 150mg tablets (Actavis UK Ltd)

	02.05.05.02.00		Irbesartan 150mg tablets (Dexcel-Pharma Ltd)
	02.05.05.02.00		Irbesartan 150mg tablets (Teva UK Ltd)
bk5y.	02050504		Irbesartan 300mg / Hydrochlorothiazide 12.5mg tablets
	02.02.01.00.00		Irbesartan 300mg / Hydrochlorothiazide 12.5mg tablets
	02.05.05.02.00		Irbesartan 300mg / Hydrochlorothiazide 12.5mg tablets (Actavis UK Ltd)
bk5x.	02050504		Irbesartan 300mg / Hydrochlorothiazide 25mg tablets
	02.05.05.02.00		Irbesartan 300mg / Hydrochlorothiazide 25mg tablets
bk53.	02050502		Irbesartan 300mg tablets
	02.05.05.02.00		Irbesartan 300mg tablets
	0205052I0AAACAC		IRBESARTAN 300MG TABLETS
	02.05.05.02.00		Irbesartan 300mg tablets (Actavis UK Ltd)
	02.05.05.02.00		Irbesartan 300mg tablets (Teva UK Ltd)
	02.05.05.02.00		Irbesartan 300mg/5ml oral suspension
	0205052I0AAAAAA		IRBESARTAN 75MG
bk51.	02050502		Irbesartan 75mg tablets
	02.05.05.02.00		Irbesartan 75mg tablets
	02.05.05.02.00		Irbesartan 75mg tablets (Actavis UK Ltd)
	02.05.05.02.00		Irbesartan 75mg tablets (Teva UK Ltd)
	0205052I0AAABAB		IRBESARTAN TABLETS 150MG
	0205052I0AAACAC		IRBESARTAN TABLETS 300MG
	0205052I0AAAAAA		IRBESARTAN TABLETS 75MG
	0205052I0AAABAB		Irbesartan TABS 150MG
	0205052I0AAACAC		Irbesartan TABS 300MG
	0205052I0AAAAAA		Irbesartan TABS 75MG
	0202040V0BBAAAA		KALSPARE TABLETS
	02.02.01.00.00		KALSPARE tablets [DOMINION]
	02.02.08.00.00		Lasikal modified-release tablets (Borg Medicare)
b519.	02020400		Lasilactone 20mg/50mg capsules (Sanofi)
	02.02.04.00.00		Lasilactone 20mg/50mg capsules (Sanofi)
	02.02.02.00.00		Lasix 20mg tablets (Borg Medicare)
	02.02.02.00.00		Lasix 40mg tablets (Sanofi)
	02.02.02.00.00		Lasoride 5mg/40mg tablets (Sanofi)
	0205051L0AAACAC		Lisinopril Tablets 10 mg
	0205051L0AAAAAA		Lisinopril Tablets 2.5 mg
	0205051L0AAADAD		Lisinopril Tablets 20 mg
	0205051L0AAABAB		Lisinopril Tablets 5 mg
bi3t.	02050501		LISINOPRIL + HYDROCHLOROTHIAZIDE tabs 10mg + 12.5mg
bi3p.	02050501		LISINOPRIL + HYDROCHLOROTHIAZIDE tabs 20mg + 12.5mg
	0205051L0AAACAC		LISINOPRIL 10 MG TAB

bi3t.	02050501		Lisinopril 10mg / Hydrochlorothiazide 12.5mg tablets
	02.02.01.00.00		Lisinopril 10mg / Hydrochlorothiazide 12.5mg tablets
	02.05.05.01.00		Lisinopril 10mg / Hydrochlorothiazide 12.5mg tablets (Teva UK Ltd)
	0205051L0AAACAC		LISINOPRIL 10MG TABLET
bi33.	02050501		Lisinopril 10mg tablets
	02.05.05.01.00		Lisinopril 10mg tablets
	02.05.05.01.00		Lisinopril 10mg tablets (Actavis UK Ltd)
	02.05.05.01.00		Lisinopril 10mg tablets (Almus Pharmaceuticals Ltd)
	02.05.05.01.00		Lisinopril 10mg tablets (Mylan Ltd)
	02.05.05.01.00		Lisinopril 10mg tablets (Ranbaxy (UK) Ltd)
	02.05.05.01.00		Lisinopril 10mg tablets (Teva UK Ltd)
	02.05.05.01.00		Lisinopril 10mg tablets (Tillomed Laboratories Ltd)
bi31.	02050501		Lisinopril 2.5mg tablets
	02.05.05.01.00		Lisinopril 2.5mg tablets
	02.05.05.01.00		Lisinopril 2.5mg tablets (Actavis UK Ltd)
	02.05.05.01.00		Lisinopril 2.5mg tablets (Almus Pharmaceuticals Ltd)
	02.05.05.01.00		Lisinopril 2.5mg tablets (Arrow Generics Ltd)
	02.05.05.01.00		Lisinopril 2.5mg tablets (Teva UK Ltd)
	02.05.05.01.00		Lisinopril 2.5mg/5ml oral solution
bi3p.	02050501		Lisinopril 20mg / Hydrochlorothiazide 12.5mg tablets
	02.02.01.00.00		Lisinopril 20mg / Hydrochlorothiazide 12.5mg tablets
	02.02.01.00.00		Lisinopril 20mg / Hydrochlorothiazide 12.5mg tablets (A A H Pharmaceuticals Ltd)
	02.05.05.01.00		Lisinopril 20mg / Hydrochlorothiazide 12.5mg tablets (Mylan Ltd)
	02.02.01.00.00		Lisinopril 20mg / Hydrochlorothiazide 12.5mg tablets (Teva UK Ltd)
bi34.	02050501		Lisinopril 20mg tablets
	02.05.05.01.00		Lisinopril 20mg tablets
	0205051L0AAADAD		LISINOPRIL 20MG TABLETS
	02.05.05.01.00		Lisinopril 20mg tablets (Actavis UK Ltd)
	02.05.05.01.00		Lisinopril 20mg tablets (Almus Pharmaceuticals Ltd)
	02.05.05.01.00		Lisinopril 20mg tablets (Mylan Ltd)
	02.05.05.01.00		Lisinopril 20mg tablets (Ranbaxy (UK) Ltd)
	02.05.05.01.00		Lisinopril 20mg tablets (Teva UK Ltd)
	0205051L0AAABAB		LISINOPRIL 5 MGM TAB
	0205051L0AAABAB		LISINOPRIL 5MG
	0205051L0AAABAB		LISINOPRIL 5MG -
bi32.	02050501		Lisinopril 5mg tablets
	02.05.05.01.00		Lisinopril 5mg tablets
	0205051L0AAABAB		LISINOPRIL 5MG TABLETS
	02.05.05.01.00		Lisinopril 5mg tablets (A A H Pharmaceuticals Ltd)

	02.05.05.01.00		Lisinopril 5mg tablets (Actavis UK Ltd)
	02.05.05.01.00		Lisinopril 5mg tablets (Almus Pharmaceuticals Ltd)
	02.05.05.01.00		Lisinopril 5mg tablets (Mylan Ltd)
	02.05.05.01.00		Lisinopril 5mg tablets (Sandoz Ltd)
	02.05.05.01.00		Lisinopril 5mg tablets (Teva UK Ltd)
	02.05.05.01.00		Lisinopril 5mg tablets (Zentiva)
	02.05.05.01.00		Lisinopril 5mg/5ml oral solution
	02.05.05.01.00		Lisinopril 5mg/5ml oral suspension
	02050501		Lisinopril And Hydrochlorothiazide Tablets 10 mg + 12.5 mg
	02050501		Lisinopril And Hydrochlorothiazide Tablets 20 mg + 12.5 mg
	02050501		Lisinopril And Hydrochlorothiazide 10mg/12.5mg TABS
	02050501		LISINOPRIL AND HYDROCHLOROTHIAZIDE 20MG/12.5MG TABLETS
	02050501		Lisinopril And Hydrochlorothiazide 20mg/12.5mg TABS
	0205051L0AAACAC		Lisinopril Non Tariff TABS 10MG
	0205051L0AAAAAA		LISINOPRIL STARTER PACK TABLETS 2.5MG
	0205051L0AAACAC		LISINOPRIL TABLETS 10MG
	0205051L0AAACAC		LISINOPRIL TABLETS 10MG-P42 0
	0205051L0AAAAAA		LISINOPRIL TABLETS 2.5MG
	02.05.05.01.00		LISINOPRIL tablets 2.5mg [ZENTIVA]
	0205051L0AAAAAA		LISINOPRIL TABLETS 2.5MG-P42 0
	0205051L0AAADAD		LISINOPRIL TABLETS 20MG
	02.05.05.01.00		LISINOPRIL tablets 20mg [NICHE]
	02.05.05.01.00		LISINOPRIL tablets 20mg [ZENTIVA]
	0205051L0AAABAB		LISINOPRIL TABLETS 5MG
	0205051L0AAABAB		LISINOPRIL TABLETS 5MG-P42 0
bi33.	02050501		LISINOPRIL tabs 10mg
	0205051L0AAACAC		Lisinopril TABS 10MG
bi31.	02050501		LISINOPRIL tabs 2.5mg
	0205051L0AAAAAA		Lisinopril TABS 2.5MG
bi34.	02050501		LISINOPRIL tabs 20mg
	0205051L0AAADAD		Lisinopril TABS 20MG
bi32.	02050501		LISINOPRIL tabs 5mg
	0205051L0AAABAB		Lisinopril TABS 5MG
	02.05.05.01.00		Lopace 10mg capsules (Discovery Pharmaceuticals)
	02.05.05.01.00		Lopace 2.5mg capsules (Discovery Pharmaceuticals)
	02.05.05.01.00		Lopace 5mg capsules (Discovery Pharmaceuticals)
	02.02.01.00.00		LOPRESORETIC tablets [NOV/GEIGY]
bk3y.	02050504		Losartan 100mg / Hydrochlorothiazide 12.5mg tablets
	02.02.01.00.00		Losartan 100mg / Hydrochlorothiazide 12.5mg tablets

	02.05.05.02.00		Losartan 100mg / Hydrochlorothiazide 12.5mg tablets (Actavis UK Ltd)
	02.02.01.00.00		Losartan 100mg / Hydrochlorothiazide 12.5mg tablets (Teva UK Ltd)
bk3z.	02050504		Losartan 100mg / Hydrochlorothiazide 25mg tablets
	02.05.05.02.00		Losartan 100mg / Hydrochlorothiazide 25mg tablets
	02.02.01.00.00		Losartan 100mg / Hydrochlorothiazide 25mg tablets (A A H Pharmaceuticals Ltd)
	02.02.01.00.00		Losartan 100mg / Hydrochlorothiazide 25mg tablets (Teva UK Ltd)
bk37.	02050502		Losartan 100mg tablets
	02.05.05.02.00		Losartan 100mg tablets
	02.05.05.02.00		Losartan 100mg tablets (A A H Pharmaceuticals Ltd)
	02.05.05.02.00		Losartan 100mg tablets (Actavis UK Ltd)
	02.05.05.02.00		Losartan 100mg tablets (Dexcel-Pharma Ltd)
	02.05.05.02.00		Losartan 100mg tablets (Teva UK Ltd)
	02.05.05.02.00		Losartan 100mg tablets (Wockhardt UK Ltd)
bk3C.	02050502		Losartan 12.5mg tablets
	02.05.05.02.00		Losartan 12.5mg tablets
	02.05.05.02.00		Losartan 12.5mg tablets (A A H Pharmaceuticals Ltd)
	02.05.05.02.00		Losartan 12.5mg tablets (Dexcel-Pharma Ltd)
bk31.	02050502		Losartan 25mg tablets
	02.05.05.02.00		Losartan 25mg tablets
	02.05.05.02.00		Losartan 25mg tablets (A A H Pharmaceuticals Ltd)
	02.05.05.02.00		Losartan 25mg tablets (Actavis UK Ltd)
	02.05.05.02.00		Losartan 25mg tablets (Dexcel-Pharma Ltd)
	02.05.05.02.00		Losartan 25mg tablets (Teva UK Ltd)
	02.05.05.02.00		Losartan 25mg tablets (Wockhardt UK Ltd)
bk35.	02050504		Losartan 50mg / Hydrochlorothiazide 12.5mg tablets
	02.02.01.00.00		Losartan 50mg / Hydrochlorothiazide 12.5mg tablets
	02.02.01.00.00		Losartan 50mg / Hydrochlorothiazide 12.5mg tablets (Actavis UK Ltd)
	02.02.01.00.00		Losartan 50mg / Hydrochlorothiazide 12.5mg tablets (Teva UK Ltd)
bk32.	02050502		Losartan 50mg tablets
	02.05.05.02.00		Losartan 50mg tablets
	02.05.05.02.00		Losartan 50mg tablets (A A H Pharmaceuticals Ltd)
	02.05.05.02.00		Losartan 50mg tablets (Actavis UK Ltd)
	02.05.05.02.00		Losartan 50mg tablets (Dexcel-Pharma Ltd)
	02.05.05.02.00		Losartan 50mg tablets (Sovereign Medical Ltd)
	02.05.05.02.00		Losartan 50mg tablets (Teva UK Ltd)
	02.05.05.02.00		Losartan 50mg tablets (Wockhardt UK Ltd)
	02050502		Losartan And Hydrochlorothiazide Tablets 100 mg + 12.5 mg

	02050502		Losartan And Hydrochlorothiazide Tablets 100 mg + 25 mg
	02050502		Losartan And Hydrochlorothiazide Tablets 50 mg + 12.5 mg
	02050502		Losartan Potassium Tablets 100 mg
	02050502		Losartan Potassium Tablets 12.5 mg
	0205052N0AAAAAA		Losartan Potassium Tablets 25 mg
	0205052N0AAABAB		Losartan Potassium Tablets 50 mg
	02050502		Losartan Potassium TABLETS 100MG
	02050502		LOSARTAN POTASSIUM TABLETS 100MG
	02050502		LOSARTAN POTASSIUM TABLETS 100MGM
	0205052N0AAAAAA		LOSARTAN POTASSIUM TABLETS 25MG
	0205052N0AAABAB		Losartan Potassium TABLETS 50MG
	0205052N0AAABAB		LOSARTAN POTASSIUM TABLETS 50MG
	02050502		Losartan Potassium TABS 100MG
	02050502		Losartan Potassium TABS 12.5MG
	0205052N0AAAAAA		Losartan Potassium TABS 25MG
	0205052N0AAABAB		Losartan Potassium TABS 50MG
	02050502		LOSARTAN TABLETS 100MG
	0205052N0AAAAAA		LOSARTAN TABLETS 25MG
	0205052N0AAABAB		LOSARTAN TABLETS 50MG
bk37.	02050502		LOSARTAN tabs 100mg
bk31.	02050502		LOSARTAN tabs 25mg
bk32.	02050502		LOSARTAN tabs 50mg
	02.05.05.02.00		Micardis 20mg tablets (Boehringer Ingelheim Ltd)
	02.05.05.02.00		Micardis 40mg tablets (Boehringer Ingelheim Ltd)
	02.05.05.02.00		Micardis 80mg tablets (Boehringer Ingelheim Ltd)
	02.02.01.00.00		MicardisPlus 40mg/12.5mg tablets (Boehringer Ingelheim Ltd)
	02.02.01.00.00		MicardisPlus 80mg/12.5mg tablets (Boehringer Ingelheim Ltd)
	02.02.01.00.00		NATRAMID tablets 2.5mg [TRINITY]
	02.02.01.00.00		Natrilix 2.5mg tablets (Servier Laboratories Ltd)
	0202010P0BBABAD		Natrilix Sr M/R tablets 1.5 mg
b286.	02020100		Natrilix SR 1.5mg tablets (Servier Laboratories Ltd)
	02.02.01.00.00		Natrilix SR 1.5mg tablets (Servier Laboratories Ltd)
	0202010P0BBABAD		NATRILIX SR TABLETS 1.5MG
	0202010P0BBABAD		Natrilix Sr TABS 1.5MG
	0202010P0BBAAAA		NATRILIX TABLETS 2.5MG
	0202010P0BBAAAA		Natrilix TABS 2.5MG
	0202010P0BBAAAA		Natrilix-P42 2.5mg tablet
	02050502		Olmesartan Medoxomil Tablets 10 mg
	02050502		Olmesartan Medoxomil Tablets 20 mg

	02050502		Olmesartan Medoxomil Tablets 40 mg
	02.05.05.02.00		Olmesartan medoxomil 10mg tablets
	02.05.05.02.00		Olmesartan medoxomil 20mg / Hydrochlorothiazide 12.5mg tablets
	02.05.05.02.00		Olmesartan medoxomil 20mg / Hydrochlorothiazide 25mg tablets
	02.05.05.02.00		Olmesartan medoxomil 20mg tablets
	02.05.05.02.00		Olmesartan medoxomil 40mg / Amlodipine 10mg tablets
	02.05.05.02.00		Olmesartan medoxomil 40mg / Amlodipine 5mg tablets
	02.02.01.00.00		Olmesartan medoxomil 40mg / Hydrochlorothiazide 12.5mg tablets
	02.05.05.02.00		Olmesartan medoxomil 40mg tablets
	02050502		OLMESARTAN MEDOXOMIL TABLETS 10MG
	02050502		OLMESARTAN MEDOXOMIL TABLETS 20MG
	02050502		OLMESARTAN MEDOXOMIL TABLETS 40MG
	02050502		Olmesartan Medoxomil TABS 10MG
	02050502		Olmesartan Medoxomil TABS 20MG
	02.02.01.00.00		olmesartan medoxomil with amlodipine and hydrochlorothiazide tablets 20mg + 5mg + 12.5mg
	02.02.01.00.00		olmesartan medoxomil with amlodipine and hydrochlorothiazide tablets 40mg + 10mg + 25mg
	02.05.05.02.00		Olmetec 10mg tablets (Daiichi Sankyo UK Ltd)
	02.05.05.02.00		Olmetec 20mg tablets (Daiichi Sankyo UK Ltd)
	02.05.05.02.00		Olmetec 40mg tablets (Daiichi Sankyo UK Ltd)
	02050502		Olmetec Plus Tablets 20 mg/25 mg
	02.05.05.02.00		Olmetec Plus 20mg/12.5mg tablets (Daiichi Sankyo UK Ltd)
	02.02.01.00.00		Olmetec Plus 20mg/25mg tablets (Daiichi Sankyo UK Ltd)
	02050502		OLMETEC TABLETS 10MG
	02050502		OLMETEC TABLETS 20MG
	0205051M0AAAAAA		PERINDOPRIL 2MG TAB
	0205051M0AAABAB		PERINDOPRIL 4 MG
	02050501		Perindopril Arginine Tablets 10 mg
	02050501		Perindopril Arginine Tablets 2.5 mg
	02050501		Perindopril Arginine Tablets 5 mg
	02.05.05.01.00		Perindopril arginine 10mg tablets
	02.05.05.01.00		Perindopril arginine 2.5mg tablets
	02.05.05.01.00		Perindopril arginine 5mg / Indapamide 1.25mg tablets
	02.05.05.01.00		Perindopril arginine 5mg tablets
	02050501		Perindopril Arginine TABS 10MG
	02050501		Perindopril Arginine TABS 5MG
	0205051M0AAAAAA		Perindopril Erbumine Tablets 2 mg
	0205051M0AAABAB		Perindopril Erbumine Tablets 4 mg
	02050501		Perindopril Erbumine Tablets 8 mg

bi51.	02050501		Perindopril erbumine 2mg tablets
	02.05.05.01.00		Perindopril erbumine 2mg tablets
	02.05.05.01.00		Perindopril erbumine 2mg tablets (A A H Pharmaceuticals Ltd)
	02.05.05.01.00		Perindopril erbumine 2mg tablets (Actavis UK Ltd)
	02.05.05.01.00		Perindopril erbumine 2mg tablets (Apotex UK Ltd)
	02.05.05.01.00		Perindopril erbumine 2mg tablets (Mylan Ltd)
	02.05.05.01.00		Perindopril erbumine 2mg tablets (Teva UK Ltd)
	02.05.05.01.00		Perindopril erbumine 4mg / Amlodipine 5mg tablets
	02.02.01.00.00		Perindopril erbumine 4mg / Indapamide 1.25mg tablets
bi52.	02050501		Perindopril erbumine 4mg tablets
	02.05.05.01.00		Perindopril erbumine 4mg tablets
	02.05.05.01.00		Perindopril erbumine 4mg tablets (A A H Pharmaceuticals Ltd)
	02.05.05.01.00		Perindopril erbumine 4mg tablets (Actavis UK Ltd)
	02.05.05.01.00		Perindopril erbumine 4mg tablets (Apotex UK Ltd)
	02.05.05.01.00		Perindopril erbumine 4mg tablets (Mylan Ltd)
	02.05.05.01.00		Perindopril erbumine 4mg tablets (Teva UK Ltd)
	02.05.05.01.00		Perindopril erbumine 4mg/5ml oral suspension
bi57.	02050501		Perindopril erbumine 8mg tablets
	02.05.05.01.00		Perindopril erbumine 8mg tablets
	02.05.05.01.00		Perindopril erbumine 8mg tablets (A A H Pharmaceuticals Ltd)
	02.05.05.01.00		Perindopril erbumine 8mg tablets (Actavis UK Ltd)
	02.05.05.01.00		Perindopril erbumine 8mg tablets (Apotex UK Ltd)
	02.05.05.01.00		Perindopril erbumine 8mg tablets (Mylan Ltd)
	02.05.05.01.00		Perindopril erbumine 8mg tablets (Teva UK Ltd)
	0205051M0AAAAAA		Perindopril Erbumine TABLETS 2MG
	0205051M0AAAAAA		PERINDOPRIL ERBUMINE TABLETS 2MG
	0205051M0AAABAB		Perindopril Erbumine TABLETS 4MG
	0205051M0AAABAB		PERINDOPRIL ERBUMINE TABLETS 4MG
	02050501		Perindopril Erbumine TABLETS 8MG
	02050501		PERINDOPRIL ERBUMINE TABLETS 8MG
bi51.	02050501		PERINDOPRIL ERBUMINE tabs 2mg
	0205051M0AAAAAA		Perindopril Erbumine TABS 2MG
bi52.	02050501		PERINDOPRIL ERBUMINE tabs 4mg
	0205051M0AAABAB		Perindopril Erbumine TABS 4MG
	02050501		Perindopril Erbumine TABS 8MG
	02.05.05.01.00		PERINDOPRIL tablets 2mg [NEOLAB]
	02.05.05.01.00		PERINDOPRIL tablets 2mg [SERVIER]
	02.05.05.01.00		PERINDOPRIL tablets 4mg [NEOLAB]
	02.05.05.01.00		PERINDOPRIL tablets 4mg [SERVIER]

	02.05.05.01.00		PERINDOPRIL tablets 8mg [NEOLAB]
	02.05.05.01.00		PERINDOPRIL tablets 8mg [SERVIER]
	0205051M0AAAAAA		Perindopril Tert-Butylamine Tablets 2 mg
	0205051M0AAABAB		Perindopril Tert-Butylamine Tablets 4 mg
	02050501		Perindopril Tert-Butylamine Tablets 8 mg
	02.02.01.00.00		Perindopril tosilate 5mg / Indapamide 1.25mg tablets
	02050501		PERINDOPRIL/INDAPAMIDE 4MG/1.25MG TABLETS
	0205051Q0AAABAB		Quinapril Tablets 10 mg
	0205051Q0AAACAC		Quinapril Tablets 20 mg
	0205051Q0AAADAD		Quinapril Tablets 40 mg
	02.02.01.00.00		Quinapril 10mg / Hydrochlorothiazide 12.5mg tablets
bi42.	02050501		Quinapril 10mg tablets
	02.05.05.01.00		Quinapril 10mg tablets
	02.05.05.01.00		Quinapril 10mg tablets (Teva UK Ltd)
bi43.	02050501		Quinapril 20mg tablets
	02.05.05.01.00		Quinapril 20mg tablets
bi4A.	02050501		Quinapril 40mg tablets
	02.05.05.01.00		Quinapril 40mg tablets
	02.05.05.01.00		Quinapril 5mg tablets
	0205051Q0AAABAB		QUINAPRIL TABLETS 10MG
	0205051Q0AAACAC		QUINAPRIL TABLETS 20MG
	0205051Q0AAADAD		QUINAPRIL TABLETS 40MG
	0205051Q0AAAAAA		QUINAPRIL TABLETS 5MG
	0205051Q0AAABAB		Quinapril TABS 10MG
	0205051Q0AAACAC		Quinapril TABS 20MG
	0205051Q0AAADAD		Quinapril TABS 40MG
	0205051Q0AAAAAA		Quinapril TABS 5MG
	0205051R0AAAAAA		Ramipril Capsules 1.25 mg
	02050501		Ramipril Capsules 10 mg
	0205051R0AAABAB		Ramipril Capsules 2.5 mg
	0205051R0AAACAC		Ramipril Capsules 5 mg
	02050501		Ramipril Suspension 5 mg/5 ml
	02050501		Ramipril Tablets 1.25 mg
	02050501		Ramipril Tablets 10 mg
	02050501		Ramipril Tablets 2.5 mg
	02050501		Ramipril Tablets 5 mg
	02050501		RAMIPRIL - TABLETS 1.25MG
	02050501		RAMIPRIL - TABLETS 10MG
	02050501		Ramipril Titration pack 7 x 2.5 mg 21 x 5 mg 7 x 10 mg
	02050501		Ramipril Titration Pack 7 x 2.5 mg 21 x 5 mg 7 x 10 mg
bi61.	02050501		Ramipril 1.25mg capsules

	02.05.05.01.00		Ramipril 1.25mg capsules
	02.05.05.01.00		Ramipril 1.25mg capsules (A A H Pharmaceuticals Ltd)
	02.05.05.01.00		Ramipril 1.25mg capsules (Actavis UK Ltd)
	02.05.05.01.00		Ramipril 1.25mg capsules (Sandoz Ltd)
	02.05.05.01.00		Ramipril 1.25mg capsules (Teva UK Ltd)
bi6B.	02050501		Ramipril 1.25mg tablets
	02.05.05.01.00		Ramipril 1.25mg tablets
	02.05.05.01.00		Ramipril 1.25mg tablets (Teva UK Ltd)
	02.05.05.01.00		Ramipril 1.25mg tablets (Zentiva)
	02050501		RAMIPRIL 10 MG
	02050501		RAMIPRIL 10 MG CAPSULES 10 MG
	02050501		RAMIPRIL 10 MGM TAB
	02050501		RAMIPRIL 10MG
	02050501		RAMIPRIL 10mg Cap
	02050501		RAMIPRIL 10MG CAP
	02050501		RAMIPRIL 10MG CAPS
bi67.	02050501		Ramipril 10mg capsules
	02.05.05.01.00		Ramipril 10mg capsules
	02050501		Ramipril 10mg capsules
	02.05.05.01.00		Ramipril 10mg capsules (A A H Pharmaceuticals Ltd)
	02.05.05.01.00		Ramipril 10mg capsules (Actavis UK Ltd)
	02.05.05.01.00		Ramipril 10mg capsules (Almus Pharmaceuticals Ltd)
	02.05.05.01.00		Ramipril 10mg capsules (Ranbaxy (UK) Ltd)
	02.05.05.01.00		Ramipril 10mg capsules (Sandoz Ltd)
	02.05.05.01.00		Ramipril 10mg capsules (Teva UK Ltd)
	02.05.05.01.00		Ramipril 10mg capsules (Zentiva)
bi6E.	02050501		Ramipril 10mg tablets
	02.05.05.01.00		Ramipril 10mg tablets
	02050501		RAMIPRIL 10MG TABLETS
	02.05.05.01.00		Ramipril 10mg tablets (Actavis UK Ltd)
	02.05.05.01.00		Ramipril 10mg tablets (IVAX Pharmaceuticals UK Ltd)
	02.05.05.01.00		Ramipril 10mg tablets (Sandoz Ltd)
	02.05.05.01.00		Ramipril 10mg tablets (Teva UK Ltd)
	02.05.05.01.00		Ramipril 10mg/5ml oral suspension
	02050501		Ramipril 2 TABLETS 2.5mg
	0205051R0AAABAB		RAMIPRIL 2.5 MG
	0205051R0AAABAB		RAMIPRIL 2.5MG
bi62.	02050501		Ramipril 2.5mg capsules
	02.05.05.01.00		Ramipril 2.5mg capsules
	02.05.05.01.00		Ramipril 2.5mg capsules (A A H Pharmaceuticals Ltd)
	02.05.05.01.00		Ramipril 2.5mg capsules (Actavis UK Ltd)

	02.05.05.01.00		Ramipril 2.5mg capsules (Ranbaxy (UK) Ltd)
	02.05.05.01.00		Ramipril 2.5mg capsules (Sandoz Ltd)
	02.05.05.01.00		Ramipril 2.5mg capsules (Teva UK Ltd)
	02.05.05.01.00		Ramipril 2.5mg capsules (Zentiva)
bi6C.	02050501		Ramipril 2.5mg tablets
	02.05.05.01.00		Ramipril 2.5mg tablets
	02.05.05.01.00		Ramipril 2.5mg tablets (Teva UK Ltd)
bi6..	02050501		Ramipril 2.5mg/5ml oral solution sugar free
	02.05.05.01.00		Ramipril 2.5mg/5ml oral solution sugar free
	02.05.05.01.00		Ramipril 2.5mg/5ml oral suspension
	0205051R0AAACAC		RAMIPRIL 5 MG
	02050501		RAMIPRIL 5 MGM TAB
bi63.	02050501		Ramipril 5mg capsules
	02.05.05.01.00		Ramipril 5mg capsules
	02.05.05.01.00		Ramipril 5mg capsules (A A H Pharmaceuticals Ltd)
	02.05.05.01.00		Ramipril 5mg capsules (Actavis UK Ltd)
	02.05.05.01.00		Ramipril 5mg capsules (Almus Pharmaceuticals Ltd)
	02.05.05.01.00		Ramipril 5mg capsules (Arrow Generics Ltd)
	02.05.05.01.00		Ramipril 5mg capsules (Sandoz Ltd)
	02.05.05.01.00		Ramipril 5mg capsules (Teva UK Ltd)
bi6D.	02050501		Ramipril 5mg tablets
	02.05.05.01.00		Ramipril 5mg tablets
	02.05.05.01.00		Ramipril 5mg tablets (Mylan Ltd)
	02.05.05.01.00		Ramipril 5mg tablets (Teva UK Ltd)
	02.05.05.01.00		Ramipril 5mg/5ml oral solution
	02.05.05.01.00		Ramipril 5mg/5ml oral suspension
bi61.	02050501		RAMIPRIL caps 1.25mg
	0205051R0AAAAAA		Ramipril CAPS 1.25MG
bi67.	02050501		RAMIPRIL caps 10mg
	02050501		Ramipril CAPS 10mg
	02050501		Ramipril CAPS 10MG
bi62.	02050501		RAMIPRIL caps 2.5mg
	0205051R0AAABAB		Ramipril CAPS 2.5mg
	0205051R0AAABAB		Ramipril CAPS 2.5MG
bi63.	02050501		RAMIPRIL caps 5mg
	0205051R0AAACAC		Ramipril CAPS 5mg
	0205051R0AAACAC		Ramipril CAPS 5MG
	0205051R0AAAAAA		Ramipril CAPSULES 1.25MG
	0205051R0AAAAAA		RAMIPRIL CAPSULES 1.25MG
	02050501		Ramipril CAPSULES 10MG
	02050501		RAMIPRIL CAPSULES 10MG

	0205051R0AAABAB		Ramipril CAPSULES 2.5MG
	0205051R0AAABAB		RAMIPRIL CAPSULES 2.5MG
	02.05.05.01.00		ramipril capsules 2.5mg + 5mg + 10mg
	0205051R0AAABAB		RAMIPRIL CAPSULES 2.5MGS
	0205051R0AAACAC		Ramipril CAPSULES 5MG
	0205051R0AAACAC		RAMIPRIL CAPSULES 5MG
	02050501		RAMIPRIL capsules CAP 10mg-P42 0
	02050501		RAMIPRIL TABLETS 2.5MG
	02050501		RAMIPRIL TABLETS 1.25 MGM
	02050501		RAMIPRIL TABLETS 1.25MG
	02050501		RAMIPRIL TABLETS 10 MG
	02050501		RAMIPRIL TABLETS 10 MGM
	02050501		RAMIPRIL TABLETS 10 MGS
	02050501		Ramipril TABLETS 10MG
	02050501		RAMIPRIL TABLETS 10mg
	02050501		RAMIPRIL TABLETS 10MG
	02050501		RAMIPRIL TABLETS 2.5 MG
	02050501		RAMIPRIL TABLETS 2.5 MGM
	02050501		Ramipril TABLETS 2.5MG
	02050501		RAMIPRIL TABLETS 2.5mg
	02050501		RAMIPRIL TABLETS 2.5MG
	02.05.05.01.00		ramipril tablets 2.5mg + 5mg + 10mg
	02050501		RAMIPRIL TABLETS 5 MG
	02050501		RAMIPRIL TABLETS 5 MGM
	02050501		RAMIPRIL TABLETS 5 MGS
	02050501		Ramipril TABLETS 5MG
	02050501		RAMIPRIL TABLETS 5MG
	02050501		RAMIPRIL TABLETS TABLETS 2.5MG
	02050501		ramipril TABS 1.25
	02050501		Ramipril TABS 1.25mg
	02050501		Ramipril TABS 1.25MG
	02050501		Ramipril TABS 10 mg
	02050501		ramipril TABS 10mg
	02050501		Ramipril TABS 10mg
	02050501		Ramipril TABS 10MG
	02050501		ramipril TABS 2.5mg
	02050501		Ramipril TABS 2.5mg
	02050501		Ramipril TABS 2.5MG
	02050501		RAMIPRIL TABS 2.5mg
	02050501		Ramipril TABS 2.5mgs
	02050501		Ramipril TABS 5 mg

	02050501		ramipril TABS 5mg
	02050501		Ramipril TABS 5mg
	02050501		Ramipril TABS 5MG
	02050501		RAMIPRIL TABS TABS 10MG
	02050501		RAMIPRIL TABS TABS 2.5MG
	02050501		RAMIPRIL TABS TABS 5MG
	02050501		Ramipril Titration Pack 35 Capsules CAPS
	02050501		RAMIPRIL TITRATION PACK 35 CAPSULES CAPS
	02050501		RAMIPRIL TITRATION PACK 35 TABLETS TABLETS
	02050502		Sacubitril And Valsartan Tablets 24 mg + 26 mg
	02050502		Sacubitril And Valsartan Tablets 49 mg + 51 mg
	02050502		Sacubitril And Valsartan Tablets 97 mg + 103 mg
	02.05.05.02.00		Sevikar 20mg/5mg tablets (Daiichi Sankyo UK Ltd)
	02.02.01.00.00		Sevikar HCT 20mg/5mg/12.5mg tablets (Daiichi Sankyo UK Ltd)
	02.05.05.02.00		Sevikar HCT 40mg/5mg/12.5mg tablets (Daiichi Sankyo UK Ltd)
	02.02.03.00.00		SPIROLONE tablets 50mg [BERK]
	020203		Spironolactone Oral solution 25 mg/5 ml
	0202030S0AAAVAV		Spironolactone Tablets 100 mg
	0202030S0AAATAT		Spironolactone Tablets 25 mg
	0202030S0AAAUAU		Spironolactone Tablets 50 mg
b433.	02020300		Spironolactone 100mg tablets
	02.02.03.00.00		Spironolactone 100mg tablets
	02.02.03.00.00		Spironolactone 100mg tablets (A A H Pharmaceuticals Ltd)
	02.02.03.00.00		Spironolactone 100mg tablets (Almus Pharmaceuticals Ltd)
	02.02.03.00.00		Spironolactone 100mg tablets (Teva UK Ltd)
b431.	02020300		Spironolactone 25mg tablets
	02.02.03.00.00		Spironolactone 25mg tablets
	02.02.03.00.00		Spironolactone 25mg tablets (A A H Pharmaceuticals Ltd)
	02.02.03.00.00		Spironolactone 25mg tablets (Actavis UK Ltd)
	02.02.03.00.00		Spironolactone 25mg tablets (Almus Pharmaceuticals Ltd)
	02.02.03.00.00		Spironolactone 25mg tablets (Teva UK Ltd)
	02.02.03.00.00		Spironolactone 25mg/5ml oral suspension
	02.02.03.00.00		Spironolactone 50mg / Furosemide 20mg capsules
b432.	02020300		Spironolactone 50mg tablets
	02.02.03.00.00		Spironolactone 50mg tablets
	02.02.03.00.00		Spironolactone 50mg tablets (Almus Pharmaceuticals Ltd)
	02.02.03.00.00		Spironolactone 50mg tablets (Teva UK Ltd)
	02.02.03.00.00		Spironolactone 50mg/5ml oral suspension
	02.02.03.00.00		Spironolactone 5mg/5ml oral suspension

	02.02.03.00.00		spironolactone capsules 100mg
	02.02.03.00.00		spironolactone suspension sugar-free 100mg/5ml
	02.02.03.00.00		spironolactone suspension sugar-free 10mg/5ml
	02.02.03.00.00		spironolactone suspension sugar-free 25mg/5ml
	02.02.03.00.00		spironolactone suspension sugar-free 50mg/5ml
	0202030S0AAAVAV		Spironolactone TABLETS 100MG
	0202030S0AAAVAV		SPIRONOLACTONE TABLETS 100MG
	0202030S0AAATAT		Spironolactone TABLETS 25MG
	0202030S0AAATAT		SPIRONOLACTONE TABLETS 25MG
	0202030S0AAAUAU		SPIRONOLACTONE TABLETS 50MG
	0202030S0AAAVAV		Spironolactone TABS 100MG
	0202030S0AAATAT		Spironolactone TABS 25MG
	0202030S0AAAUAU		Spironolactone TABS 50MG
	0202030S0AAAUAU		Spironolactone-P42 50mg tablet
	02.02.03.00.00		Spirospare 100 tablets (Ashbourne Pharmaceuticals Ltd)
	02.05.05.01.00		Staril 10mg tablets (Bristol-Myers Squibb Pharmaceuticals Ltd)
	02.05.05.01.00		Staril 20mg tablets (Bristol-Myers Squibb Pharmaceuticals Ltd)
	0205051J0BBAAAA		Staril TABS 10MG
	0205051J0BBABAB		Staril TABS 20MG
	02050501		Tanatril Tablets 10 mg
	02.05.05.01.00		Tanatril 10mg tablets (Chiesi Ltd)
	02050501		TANATRIL TABLETS 10MG
	02.05.05.01.00		Tarka modified-release capsules (Abbott Laboratories Ltd)
	02050502		Telmisartan Tablets 20 mg
	02050502		Telmisartan Tablets 40 mg
	02050502		Telmisartan Tablets 80 mg
	02.05.05.02.00		Telmisartan 20mg tablets
	02.05.05.02.00		Telmisartan 40mg / Hydrochlorothiazide 12.5mg tablets
bk81.	02050502		Telmisartan 40mg tablets
	02.05.05.02.00		Telmisartan 40mg tablets
	02.02.01.00.00		Telmisartan 80mg / Hydrochlorothiazide 12.5mg tablets
	02.02.01.00.00		Telmisartan 80mg / Hydrochlorothiazide 25mg tablets
bk82.	02050502		Telmisartan 80mg tablets
	02.05.05.02.00		Telmisartan 80mg tablets
	02050502		TELMISARTAN TABLETS 20MG
	02050502		TELMISARTAN TABLETS 40MG
	02050502		TELMISARTAN TABLETS 80MG
	02050502		Telmisartan TABS 20MG
	02050502		Telmisartan TABS 40MG
	02050502		Telmisartan TABS 80MG

	02.05.05.02.00		telmisartan with amlodipine (roi) tablets 80mg + 5mg
	02050502		Teveten Tablets 600 mg
bk91.	02050502		Teveten 300mg tablets (Abbott Healthcare Products Ltd)
	02.05.05.02.00		Teveten 300mg tablets (BGP Products Ltd)
	02.05.05.02.00		Teveten 600mg tablets (BGP Products Ltd)
	02050502		Teveten TABS 600MG
	0202020U0AAAAAA		Torasemide Tablets 2.5 mg
	0202020U0AAABAB		Torasemide Tablets 5 mg
b358.	02020200		Torasemide 10mg tablets
	02.02.02.00.00		Torasemide 10mg tablets
b356.	02020200		Torasemide 2.5mg tablets
	02.02.02.00.00		Torasemide 2.5mg tablets
	02.02.02.00.00		Torasemide 5mg tablets
	0202020U0AAAAAA		TORASEMIDE TABLETS 2.5MG
	02.02.02.00.00		Torem 2.5mg tablets (Meda Pharmaceuticals Ltd)
	02.02.02.00.00		Torem 5mg tablets (Meda Pharmaceuticals Ltd)
	0205051U0AAACAC		Trandolapril Capsules 2 mg
	02050501		Trandolapril Capsules 4 mg
bi92.	02050501		Trandolapril 1mg capsules
	02.05.05.01.00		Trandolapril 1mg capsules
	02.05.05.01.00		Trandolapril 1mg capsules (Actavis UK Ltd)
bi93.	02050501		Trandolapril 2mg capsules
	02.05.05.01.00		Trandolapril 2mg capsules
bi9z.	02050501		Trandolapril 4mg capsules
	02.05.05.01.00		Trandolapril 4mg capsules
bi91.	02050501		Trandolapril 500microgram capsules
	02.05.05.01.00		Trandolapril 500microgram capsules
	0205051U0AAABAB		Trandolapril CAPS 1MG
	0205051U0AAACAC		Trandolapril CAPS 2MG
	02050501		Trandolapril CAPS 4MG
	0205051U0AAAAAA		Trandolapril CAPS 500MICROGRAMS
	0205051U0AAABAB		Trandolapril CAPSULES 1MG
	0205051U0AAABAB		TRANDOLAPRIL CAPSULES 1MG
	0205051U0AAACAC		Trandolapril CAPSULES 2MG
	0205051U0AAACAC		TRANDOLAPRIL CAPSULES 2MG
	02050501		Trandolapril CAPSULES 4MG
	02050501		TRANDOLAPRIL CAPSULES 4MG
	0205051U0AAAAAA		TRANDOLAPRIL CAPSULES 500MICROGRAMS
	02050501		Tritace Tablet Titration Pack 7 x 2.5 mg 21 x 5 mg 7 x 10 mg
	02.05.05.01.00		Tritace 1.25mg capsules (Aventis Pharma)
	02.05.05.01.00		Tritace 1.25mg tablets (Sanofi)

	02.05.05.01.00		Tritace 10mg capsules (Sanofi)
	02.05.05.01.00		Tritace 10mg tablets (Sanofi)
	02.05.05.01.00		Tritace 2.5mg capsules (Sanofi)
	02.05.05.01.00		Tritace 2.5mg tablets (Sanofi)
	02.05.05.01.00		Tritace 5mg capsules (Sanofi)
	02.05.05.01.00		Tritace 5mg tablets (Sanofi)
	02050501		Tritace CAPS 1.25MG
	02050501		Tritace CAPS 5MG
	02050501		TRITACE TABLETS 10MG
	02050501		TRITACE TABLETS 5MG
	02050501		TRITACE TITRATION PACK 35 CAPSULES CAPS
	02.05.05.01.00		Tritace titration pack capsules (Sanofi)
bi6o.	02050501		Tritace titration pack tablets (Sanofi)
	02.05.05.01.00		Tritace titration pack tablets (Sanofi)
	0205052V0AAACAC		Valsartan Capsules 160 mg
	0205052V0AAAAAA		Valsartan Capsules 40 mg
	0205052V0AAABAB		Valsartan Capsules 80 mg
	02050502		Valsartan Tablets 160 mg
	02050502		Valsartan Tablets 320 mg
	02050502		Valsartan Tablets 40 mg
bk4z.	02050502		Valsartan 160mg / Hydrochlorothiazide 12.5mg tablets
bk4z.	02050504		Valsartan 160mg / Hydrochlorothiazide 12.5mg tablets
	02.05.05.02.00		Valsartan 160mg / Hydrochlorothiazide 12.5mg tablets
	02.02.01.00.00		Valsartan 160mg / Hydrochlorothiazide 25mg tablets
bk43.	02050502		Valsartan 160mg capsules
	02.05.05.02.00		Valsartan 160mg capsules
	02.05.05.02.00		Valsartan 160mg capsules (Actavis UK Ltd)
	02.05.05.02.00		Valsartan 160mg capsules (Teva UK Ltd)
	02.05.05.02.00		Valsartan 160mg tablets
bk4v.	02050502		Valsartan 320mg tablets
	02.05.05.02.00		Valsartan 320mg tablets
	02.05.05.02.00		Valsartan 320mg tablets (Actavis UK Ltd)
	02.05.05.02.00		Valsartan 320mg tablets (Teva UK Ltd)
bk41.	02050502		Valsartan 40mg capsules
	02.05.05.02.00		Valsartan 40mg capsules
	02.05.05.02.00		Valsartan 40mg capsules (Teva UK Ltd)
bk4w.	02050502		Valsartan 40mg tablets
	02.05.05.02.00		Valsartan 40mg tablets
	02.05.05.02.00		Valsartan 40mg tablets (Actavis UK Ltd)
	02.05.05.02.00		Valsartan 40mg tablets (Teva UK Ltd)
bk4x.	02050504		Valsartan 80mg / Hydrochlorothiazide 12.5mg tablets

	02.02.01.00.00		Valsartan 80mg / Hydrochlorothiazide 12.5mg tablets
	02.05.05.02.00		Valsartan 80mg / Hydrochlorothiazide 12.5mg tablets (Actavis UK Ltd)
bk42.	02050502		Valsartan 80mg capsules
	02.05.05.02.00		Valsartan 80mg capsules
	02.05.05.02.00		Valsartan 80mg capsules (Actavis UK Ltd)
	02.05.05.02.00		Valsartan 80mg capsules (Teva UK Ltd)
bk4s.	02050502		Valsartan 80mg tablets
	02.05.05.02.00		Valsartan 80mg tablets
	02050502		Valsartan And Hydrochlorothiazide Tablets 160 mg + 12.5 mg
bk43.	02050502		VALSARTAN caps 160mg
	0205052V0AAACAC		Valsartan CAPS 160MG
	0205052V0AAAAAA		Valsartan CAPS 40MG
	0205052V0AAABAB		Valsartan CAPS 80MG
	0205052V0AAACAC		VALSARTAN CAPSULES 160MG
	0205052V0AAAAAA		VALSARTAN CAPSULES 40MG
	0205052V0AAABAB		VALSARTAN CAPSULES 80MG
	02050502		VALSARTAN TABLETS 40MG
	02050502		Valsartan TABS 320MG
	02.05.05.01.00		Vascace 1mg tablets (Roche Products Ltd)
	02.05.05.01.00		Vascace 2.5mg tablets (Roche Products Ltd)
	02.05.05.01.00		Vascace 5mg tablets (Roche Products Ltd)
	0202010Y0AAAAAA		Xipamide Tablets 20 mg
	02.02.01.00.00		Xipamide 20mg tablets
	0202010Y0AAAAAA		Xipamide TABS 20MG
bi3s.	02050501		Zestoretic 10 tablets (AstraZeneca UK Ltd)
	02.02.01.00.00		Zestoretic 10 tablets (AstraZeneca UK Ltd)
	0205051K0BBABAB		Zestoretic 10 TABS
	02.05.05.01.00		ZESTORETIC 10-tablets 10mg + 12.5mg [ASTRAZENEC]
	0205051K0BBAAAA		Zestoretic 20 Tablets
	0205051K0BBAAAA		ZESTORETIC 20 TABLETS
bi3n.	02050501		Zestoretic 20 tablets (AstraZeneca UK Ltd)
	02.02.01.00.00		Zestoretic 20 tablets (AstraZeneca UK Ltd)
	0205051K0BBAAAA		Zestoretic 20 TABS
	02.05.05.01.00		ZESTORETIC 20-tablets 20mg + 12.5mg [ASTRAZENEC]
	0205051L0BBACAC		Zestril Tablets 10 mg
bi3h.	02050501		Zestril 10mg tablets (AstraZeneca UK Ltd)
	02.05.05.01.00		Zestril 10mg tablets (AstraZeneca UK Ltd)
	02.05.05.01.00		Zestril 2.5mg tablets (AstraZeneca UK Ltd)
	02.05.05.01.00		Zestril 20mg tablets (AstraZeneca UK Ltd)
bi3f.	02050501		Zestril 5mg tablets (AstraZeneca UK Ltd)

	02.05.05.01.00		Zestril 5mg tablets (AstraZeneca UK Ltd)
	0205051L0BBACAC		ZESTRIL TABLETS 10MG
	0205051L0BBADAD		ZESTRIL TABLETS 20MG
	0205051L0BBACAC		Zestril TABS 10MG
	0205051L0BBADAD		Zestril TABS 20MG
	0205051L0BBABAB		Zestril TABS 5MG
	0205051L0BBABAB		Zestril-P42 5mg tablet
**Diabetes**			
	06010102		Abasaglar Solution for injection 100 units/ml 3 ml cartridge
f29C.	06010102		Abasaglar KwikPen 100units/ml solution for injection 3ml ...
	06.01.01.02.00		Abasaglar KwikPen 100units/ml solution for injection 3ml pre-filled pen (Eli Lilly and Company Ltd)
	0601023A0AAABAB		Acarbose Tablets 100 mg
	0601023A0AAAAAA		Acarbose Tablets 50 mg
	06.01.02.03.00		Acarbose 100mg tablets
	06.01.02.03.00		Acarbose 50mg tablets
	0601023A0AAABAB		ACARBOSE TABLETS 100MG
	0601023A0AAAAAA		ACARBOSE TABLETS 50MG
	0601023A0AAABAB		Acarbose TABS 100MG
	0601023A0AAAAAA		Acarbose TABS 50MG
	060106		Accu-Chek Aviva Blood Glucose Testing System
	060106		Accu-Chek Compact Meter
	060106		ACCU-CHEK COMPACT GLUCOSE STRIPS COMPACT GLUCOSE STRIPS
	060106		Accu-Chek Compact Plus Blood Glucose Testing System
ft51.	06010203		Actos 15mg tablets (Takeda UK Ltd)
	06.01.02.03.00		Actos 15mg tablets (Takeda UK Ltd)
	06.01.02.03.00		Actos 30mg tablets (Takeda UK Ltd)
ft53.	06010203		Actos 45mg tablets (Takeda UK Ltd)
	06.01.02.03.00		Actos 45mg tablets (Takeda UK Ltd)
	06.01.03.00.00		Actrapid 100units/ml solution for injection 10ml vials (Novo Nordisk Ltd)
	06.01.01.01.00		ACTRAPID injection 100 units/ml [NOVO]
	06.01.01.01.00		ACTRAPID MC injection 100 units/ml [ARUN]
	06.01.01.01.00		Actrapid NovoLet 100units/ml solution for injection (Novo Nordisk Ltd)
	06010101		Actrapid Novolet Pen 3ml INJ 100UNITS/ML
	06010101		Actrapid Penfill Injection 100 units/ml 3 ml cartridge
	06.01.01.01.00		ACTRAPID PENFILL 100 units/ml [NOVO]
	06.01.01.01.00		Actrapid Penfill 100units/ml solution for injection 3ml cartridges (Novo Nordisk Ltd)
	06010101		ACTRAPID PENFILL 3ML INJ 100UNITS/ML

	06010101		ACTRAPID PRELOADED PEN 3ML INJ 100UNITS/ML 5
	06010203		Alogliptin Tablets 25 mg
	06.01.02.03.00		Alogliptin 12.5mg / Metformin 1g tablets
ftk4.	06010203		Alogliptin 12.5mg tablets
	06.01.02.03.00		Alogliptin 12.5mg tablets
ftk6.	06010203		Alogliptin 25mg tablets
	06.01.02.03.00		Alogliptin 25mg tablets
ftk2.	06010203		Alogliptin 6.25mg tablets
	06.01.02.03.00		Alogliptin 6.25mg tablets
	06.01.02.01.00		Amaryl 1mg tablets (Zentiva)
	06.01.02.01.00		Amaryl 2mg tablets (Zentiva)
	06.01.02.01.00		Amaryl 3mg tablets (Zentiva)
	06.01.02.01.00		Amaryl 4mg tablets (Zentiva)
	06.01.02.01.00		AMARYL tablets 1mg [AVENTIS]
	06.01.02.01.00		AMARYL tablets 2mg [AVENTIS]
	06.01.02.01.00		AMARYL tablets 3mg [AVENTIS]
	06.01.02.01.00		AMARYL tablets 4mg [AVENTIS]
	06010101		Apidra Injection 100 units/ml 3 ml cartridge
f151.	06010101		Apidra 100units/ml solution for injection 10ml vials (San...
	06.01.01.01.00		Apidra 100units/ml solution for injection 10ml vials (Sanofi)
	06.01.01.01.00		Apidra 100units/ml solution for injection 3ml cartridges (Sanofi)
	06.01.01.01.00		Apidra 100units/ml solution for injection 3ml OptiClik cartridges (Sanofi)
	06.01.01.01.00		Apidra 100units/ml solution for injection 3ml pre-filled OptiSet pen (Sanofi)
	06.01.01.01.00		Apidra 100units/ml solution for injection 3ml pre-filled SoloStar pen (Sanofi)
	06010101		Apidra Solostar Pre-filled Disposable Pen 100 units/ml 3 ml pen
	06010203		Avandamet Tablets 1 mg + 500 mg
	06010203		Avandamet Tablets 2 mg + 1 gram
	06010203		Avandamet Tablets 2 mg + 500 mg
	06010203		Avandamet Tablets 4 mg + 1 gram
	06.01.02.03.00		Avandamet 1mg/500mg tablets (GlaxoSmithKline UK Ltd)
	06010203		AVANDAMET 2MG/1000MG TABLETS
	06.01.02.03.00		Avandamet 2mg/1000mg tablets (GlaxoSmithKline UK Ltd)
	06010203		Avandamet 2mg/1000mg TABS
	06.01.02.03.00		Avandamet 2mg/500mg tablets (GlaxoSmithKline UK Ltd)
	06010203		Avandamet 2mg/500mg TABS
	06010203		AVANDAMET 4MG/1000MG TABLETS

	06.01.02.03.00		Avandamet 4mg/1000mg tablets (GlaxoSmithKline UK Ltd)
	06010203		Avandamet 4mg/1000mg TABS
	06010203		AVANDAMET TABLETS 1G:2MG
	06010203		AVANDAMET TABLETS 500MG : 1MG
	06010203		AVANDAMET TABLETS 500MG : 2MG
	06010203		AVANDAMET TABS 1MG/500MG
	06010203		avandamet TABS 2/1000
	06010203		Avandamet TABS 2/1000
	06010203		Avandamet TABS 2mg/500mg
	06010203		Avandamet TABS 4/1000
	06.01.02.03.00		Avandia 4mg tablets (GlaxoSmithKline UK Ltd)
	06.01.02.03.00		Avandia 8mg tablets (GlaxoSmithKline UK Ltd)
	06.01.02.02.00		Bolamyn SR 1000mg tablets (Teva UK Ltd)
	06.01.02.02.00		Bolamyn SR 500mg tablets (Teva UK Ltd)
	06010203		Bydureon Powder and solvent for suspension for injection 2 mg pen
	06010203		Bydureon Powder and solvent for suspension for injection 2 mg vial
	06010203		Bydureon Prolonged release suspension for injection 2 mg pen
ft94.	06010203		Bydureon 2mg powder and solvent for prolonged-release sus...
	06.01.02.03.00		Bydureon 2mg powder and solvent for prolonged-release suspension for injection pre-filled pen (AstraZeneca UK Ltd)
ft93.	06010203		Bydureon 2mg powder and solvent for suspension for inject...
ft94.	06010203		Bydureon 2mg powder and solvent for suspension for inject...
	06.01.02.03.00		Bydureon 2mg powder and solvent for suspension for prolonged-release injection vials (AstraZeneca UK Ltd)
	06010203		Byetta Injection (Pre-Filled Pen) 10 micrograms/dose 60 dose pen
	06010203		Byetta Injection (Pre-Filled Pen) 5 micrograms/dose 60 dose pen
ft92.	06010203		Byetta 10micrograms/0.04ml solution for injection 2.4ml p...
	06.01.02.03.00		Byetta 10micrograms/0.04ml solution for injection 2.4ml pre-filled disposable devices (AstraZeneca UK Ltd)
ft91.	06010203		Byetta 5micrograms/0.02ml solution for injection 1.2ml pr...
	06.01.02.03.00		Byetta 5micrograms/0.02ml solution for injection 1.2ml pre-filled disposable devices (AstraZeneca UK Ltd)
	06010203		Canagliflozin Tablets 100 mg
	06010203		Canagliflozin Tablets 300 mg
ftn2.	06010203		Canagliflozin 100mg tablets

	06.01.02.03.00		Canagliflozin 100mg tablets
ftn4.	06010203		Canagliflozin 300mg tablets
	06.01.02.03.00		Canagliflozin 300mg tablets
	06.01.02.02.00		Canagliflozin 50mg / Metformin 1g tablets
	06.01.02.02.00		Canagliflozin 50mg / Metformin 850mg tablets
	06.01.02.01.00		Chlorpropamide 100mg tablets
	06.01.02.01.00		Chlorpropamide 250mg tablets
	0601021E0AAABAB		Chlorpropamide TABS 250MG
	06010203		Competact Tablets 15 mg + 850 mg
ft71.	06010203		Competact 15mg/850mg tablets (Takeda UK Ltd)
	06.01.02.02.00		Competact 15mg/850mg tablets (Takeda UK Ltd)
	06010203		Competact 15mg/850mg TABS
	06.01.02.01.00		Daonil 5mg tablets (Sanofi)
	06010203		Dapagliflozin Tablets 10 mg
	06010203		Dapagliflozin Tablets 5 mg
fth4.	06010203		Dapagliflozin 10mg tablets
	06.01.02.03.00		Dapagliflozin 10mg tablets
	06.01.02.03.00		Dapagliflozin 5mg / Metformin 1g tablets
	06.01.02.03.00		Dapagliflozin 5mg / Metformin 850mg tablets
fth3.	06010203		Dapagliflozin 5mg tablets
	06.01.02.03.00		Dapagliflozin 5mg tablets
	06010203		Dapagliflozin And Metformin Tablets 5 mg + 1000 mg
	06.01.02.01.00		Diabetamide 2.5mg tablets (Ashbourne Pharmaceuticals Ltd)
	06.01.02.01.00		Diabetamide 5mg tablets (Ashbourne Pharmaceuticals Ltd)
	06.01.02.01.00		DIABINESE tablets 100mg [PFIZER]
	06.01.02.01.00		DIABINESE tablets 250mg [PFIZER]
	06.01.02.02.00		Diagemet XL 500mg tablets (Genus Pharmaceuticals Ltd)
	06.01.02.01.00		Diaglyk 80mg tablets (Ashbourne Pharmaceuticals Ltd)
	0601021M0BBAAAA		Diamicron Tablets 80 mg
	06010201		Diamicron 30 mg Mr M/R tablets 30 mg
f354.	06010201		Diamicron 30mg MR tablets (Servier Laboratories Ltd)
	06.01.02.01.00		Diamicron 30mg MR tablets (Servier Laboratories Ltd)
	06.01.02.01.00		Diamicron 80mg tablets (Servier Laboratories Ltd)
	06010201		Diamicron Mr TABLETS 30MG
	06010201		DIAMICRON MR TABLETS 30MG
	06010201		Diamicron Mr TABS 30MG
	0601021M0BBAAAA		DIAMICRON TABLETS 80MG
	0601021M0BBAAAA		Diamicron TABS 80MG
	06.01.04.00.00		Diazoxide 50mg tablets

	06010203		Dulaglutide Solution for injection 0.75 mg/0.5 ml pre-filled device
	06010203		Dulaglutide Solution for injection 1.5 mg/0.5 ml pre-filled device
ftq2.	06010203		Dulaglutide 0.75mg/0.5ml solution for injection pre-fille...
	06.01.02.03.00		Dulaglutide 0.75mg/0.5ml solution for injection pre-filled disposable devices
	06.01.02.03.00		Dulaglutide 1.5mg/0.5ml solution for injection pre-filled disposable devices
ftq4.	06010203		Dulaglutide 1.5mg/0.5ml solution for injection pre-filled...
	06010203		Empagliflozin Tablets 10 mg
	06010203		Empagliflozin Tablets 25 mg
fto2.	06010203		Empagliflozin 10mg tablets
	06.01.02.03.00		Empagliflozin 10mg tablets
fto4.	06010203		Empagliflozin 25mg tablets
	06.01.02.03.00		Empagliflozin 25mg tablets
	06.01.02.03.00		Empagliflozin 5mg / Metformin 1g tablets
	06010203		Eucreas Tablets 1 gram + 50 mg
	06.01.02.02.00		Eucreas 50mg/1000mg tablets (Novartis Pharmaceuticals UK Ltd)
	06.01.02.01.00		Euglucon 2.5mg tablets (Aventis Pharma)
	06.01.02.01.00		Euglucon 5mg tablets (Sanofi)
	06010203		Exenatide Injection (Pre-Filled Pen) 10 micrograms/dose 60 dose pen
	06010203		Exenatide Injection (Pre-Filled Pen) 5 micrograms/dose 60 dose pen
	06010203		Exenatide Powder and solvent for suspension for injection 2 mg device
	06010203		Exenatide Powder and solvent for suspension for injection 2 mg vial
	06010203		Exenatide Prolonged release suspension for injection 2 mg device
	06010203		Exenatide Prolonged release suspension for injection 2 mg vial
ft9y.	06010203		Exenatide 10micrograms/0.04ml solution for injection 2.4m...
	06.01.02.03.00		Exenatide 10micrograms/0.04ml solution for injection 2.4ml pre-filled disposable devices
ft95.	06010203		Exenatide 2mg powder and solvent for suspension for injec...
ft9x.	06010203		Exenatide 2mg powder and solvent for suspension for injec...
	06.01.02.03.00		Exenatide 2mg powder and solvent for suspension for injection pre-filled disposable devices
	06.01.02.03.00		Exenatide 2mg powder and solvent for suspension for injection vials
	06.01.02.03.00		Exenatide 5micrograms/0.02ml solution for injection 1.2ml pre-filled disposable devices

ft9z.	06010203		Exenatide 5micrograms/0.02ml solution for injection 1.2ml...
	06010203		Exenatide 60 Dose Prefilled Pen 1.2ml INJ 5MICROGRAMS
	06010203		EXENATIDE 60 DOSE PREFILLED PEN 1.2ML INJ 5MICROGRAMS
	06010203		Exenatide 60 Dose Prefilled Pen 2.4ml INJ 10MICROGRAMS
	06010203		EXENATIDE 60 DOSE PREFILLED PEN 2.4ML INJ 10MICROGRAMS
fth2.	06010203		Forxiga 10mg tablets (AstraZeneca UK Ltd)
	06.01.02.03.00		Forxiga 10mg tablets (AstraZeneca UK Ltd)
	06.01.02.03.00		Forxiga 5mg tablets (AstraZeneca UK Ltd)
	06.01.02.03.00		Galvus 50mg tablets (Novartis Pharmaceuticals UK Ltd)
	06010203		GALVUS TABLETS 50MG
	0601021H0AAAAAA		Glibenclamide Tablets 2.5 mg
	0601021H0AAABAB		Glibenclamide Tablets 5 mg
	06.01.02.01.00		Glibenclamide 2.5mg tablets
f332.	06010201		Glibenclamide 5mg tablets
	06.01.02.01.00		Glibenclamide 5mg tablets
	0601021H0AAAAAA		GLIBENCLAMIDE TABLETS 2.5MG
	0601021H0AAABAB		GLIBENCLAMIDE TABLETS 5MG
	0601021H0AAAAAA		Glibenclamide TABS 2.5MG
	0601021H0AAABAB		Glibenclamide TABS 5MG
	0601021H0AAABAB		GLIBENCLAMIDE-P42 5MG TABLET
	06.01.02.01.00		Glibenese 5mg tablets (Pfizer Ltd)
	06010201		Gliclazide M/R tablets 30 mg
	06010201		Gliclazide M/R Tablets 30 mg
	06010201		Gliclazide Tablets 40 mg
	0601021M0AAAAAA		Gliclazide Tablets 80 mg
f35y.	06010201		Gliclazide 30mg modified-release tablets
	06.01.02.01.00		Gliclazide 30mg modified-release tablets
	06.01.02.01.00		Gliclazide 30mg modified-release tablets (A A H Pharmaceuticals Ltd)
	06.01.02.01.00		Gliclazide 30mg modified-release tablets (Actavis UK Ltd)
f35x.	06010201		Gliclazide 40mg tablets
	06.01.02.01.00		Gliclazide 40mg tablets
	06.01.02.01.00		Gliclazide 40mg/5ml oral suspension
f35w.	06010201		Gliclazide 60mg modified-release tablets
	06.01.02.01.00		Gliclazide 60mg modified-release tablets
f35z.	06010201		Gliclazide 80mg tablets
	06.01.02.01.00		Gliclazide 80mg tablets
	06.01.02.01.00		Gliclazide 80mg tablets (A A H Pharmaceuticals Ltd)
	06.01.02.01.00		Gliclazide 80mg tablets (Actavis UK Ltd)

	06.01.02.01.00		Gliclazide 80mg tablets (Almus Pharmaceuticals Ltd)
	06.01.02.01.00		Gliclazide 80mg tablets (Mylan Ltd)
	06.01.02.01.00		Gliclazide 80mg tablets (PLIVA Pharma Ltd)
	06.01.02.01.00		Gliclazide 80mg tablets (Sandoz Ltd)
	06.01.02.01.00		Gliclazide 80mg tablets (Teva UK Ltd)
	0601021M0AAAAAA		GLICLAZIDE 80MG TABLETS-P42 0
	06.01.02.01.00		Gliclazide 80mg/5ml oral suspension
	06010201		GLICLAZIDE MR TABLETS 30MG
	06010201		Gliclazide Mr TABS 30MG
	0601021M0AAAAAA		Gliclazide TABLETS 80MG
	0601021M0AAAAAA		GLICLAZIDE TABLETS 80MG
	0601021M0AAAAAA		GLICLAZIDE TABLETS 80MG-P42 0
f35z.	06010201		GLICLAZIDE tabs 80mg
	0601021M0AAAAAA		Gliclazide TABS 80MG
	0601021A0AAABAB		Glimepiride Tablets 1 mg
	0601021A0AAAAAA		Glimepiride Tablets 2 mg
	0601021A0AAADAD		Glimepiride Tablets 4 mg
f3A3.	06010201		Glimepiride 1mg tablets
	06.01.02.01.00		Glimepiride 1mg tablets
	06.01.02.01.00		Glimepiride 1mg tablets (A A H Pharmaceuticals Ltd)
f3A1.	06010201		Glimepiride 2mg tablets
	06.01.02.01.00		Glimepiride 2mg tablets
	06.01.02.01.00		Glimepiride 2mg tablets (A A H Pharmaceuticals Ltd)
	06.01.02.01.00		Glimepiride 2mg tablets (Teva UK Ltd)
	06.01.02.01.00		Glimepiride 2mg tablets (Zentiva)
	06.01.02.01.00		Glimepiride 3mg tablets
f3A5.	06010201		Glimepiride 4mg tablets
	06.01.02.01.00		Glimepiride 4mg tablets
	0601021A0AAACAC		GLIMEPIRIDE TABLETS 3MG
	0601021A0AAADAD		GLIMEPIRIDE TABLETS 4MG
	0601021P0AAABAB		Glipizide Tablets 5 mg
	06.01.02.01.00		Glipizide 2.5mg tablets
f361.	06010201		Glipizide 5mg tablets
	06.01.02.01.00		Glipizide 5mg tablets
	06.01.02.01.00		Glipizide 5mg tablets (Actavis UK Ltd)
	0601021P0AAAAAA		GLIPIZIDE TABLETS 2.5MG
	0601021P0AAABAB		Glipizide TABLETS 5MG
	0601021P0AAABAB		GLIPIZIDE TABLETS 5MG
	0601021P0AAAAAA		Glipizide TABS 2.5MG
f361.	06010201		GLIPIZIDE tabs 5mg
	0601021P0AAABAB		Glipizide TABS 5MG

	06.01.02.03.00		Glucobay 100mg tablets (Bayer Plc)
	06.01.02.03.00		Glucobay 50mg tablets (Bayer Plc)
	0601023A0BBACAA		Glucobay TABS 50MG
	0601022B0BBAAAB		Glucophage Tablets 500 mg
	06.01.02.02.00		Glucophage 1000mg oral powder sachets (Merck Serono Ltd)
	06.01.02.02.00		Glucophage 500mg oral powder sachets (Merck Serono Ltd)
f411.	06010202		Glucophage 500mg tablets (Merck Serono Ltd)
	06.01.02.02.00		Glucophage 500mg tablets (Merck Serono Ltd)
	06.01.02.02.00		Glucophage 850mg tablets (Merck Serono Ltd)
	06010202		Glucophage Sr M/R tablets 1 gram
	06010202		Glucophage Sr M/R tablets 500 mg
	06010202		Glucophage Sr M/R tablets 750 mg
f41A.	06010202		Glucophage SR 1000mg tablets (Merck Serono Ltd)
	06.01.02.02.00		Glucophage SR 1000mg tablets (Merck Serono Ltd)
f417.	06010202		Glucophage SR 500mg tablets (Merck Serono Ltd)
	06.01.02.02.00		Glucophage SR 500mg tablets (Merck Serono Ltd)
f419.	06010202		Glucophage SR 750mg tablets (Merck Serono Ltd)
	06.01.02.02.00		Glucophage SR 750mg tablets (Merck Serono Ltd)
	06010202		GLUCOPHAGE SR TABLETS 500MG
	06010202		Glucophage Sr TABS 1G
	06010202		Glucophage Sr TABS 500MG
	06010202		Glucophage SR TABS 500mg
	06010202		glucophage SR TABS 500MGS
	0601022B0BBAAAB		GLUCOPHAGE TABLETS 500MG
	0601022B0BBAAAB		Glucophage TABS 500MG
	0601060D0BLAAA0		Glucotide Test strips
	0601060D0BLAAA0		Glucotide Strip Blood Glucose Testing 50
	06.01.01.02.00		HUMAJECT I pen 100 units/ml [LILLY]
	06.01.01.02.00		HumaJect M3 Pen 100units/ml suspension for injection (Eli Lilly and Company Ltd)
	06.01.01.01.00		HumaJect S Pen 100units/ml solution for injection (Eli Lilly and Company Ltd)
	0601011L0BBAAAA		Humalog Cartridges (1.5 Ml) 100 units/ml
	06010101		Humalog Cartridges (3 Ml) 100 units/ml
	0601011L0BBABAB		Humalog Injection 100 units/ml
	06010101		Humalog Solution for injection 100 units/ml 3 ml pre-filled pen
f132.	06010101		Humalog 100units/ml solution for injection 10ml vials (El...
	06.01.01.01.00		Humalog 100units/ml solution for injection 10ml vials (Eli Lilly and Company Ltd)

	06.01.01.01.00		Humalog 100units/ml solution for injection 3ml cartridges (Eli Lilly and Company Ltd)
f135.	06010101		Humalog 100units/ml solution for injection 3ml cartridges...
	0601011L0BBAAAA		Humalog Cartridge 1.5ml INJ 100UNITS/ML
	06010101		Humalog Cartridge 3ml INJ 100UNITS/ML
	06010101		HUMALOG CARTRIDGE 3ML INJ 100UNITS/ML
f135.	06010101		HUMALOG CARTRIDGE inj soln 100 units/ml
	06.01.01.01.00		HUMALOG injection 100 units/ml [LILLY]
	06010101		Humalog Kwikpen Solution for injection 100 units/ml 3 ml pre-filled pen
f138.	06010101		Humalog KwikPen 100units/ml solution for injection 3ml pr...
	06.01.01.01.00		Humalog KwikPen 100units/ml solution for injection 3ml pre-filled pen (Eli Lilly and Company Ltd)
	06010101		Humalog Kwikpen 3ml INJ 100UNITS/ML
	06010102		Humalog Mix 25 Cartridge 3ml INJ 100UNITS/ML
	06010102		HUMALOG MIX 25 CARTRIDGE 3ML INJ 100UNITS/ML
	06.01.01.02.00		HUMALOG MIX 25 injection 25:75; 100 units/ml [LILLY]
	06010102		HUMALOG MIX 25 KWICKPEN 3ML INJ 100UNITS/ML
	06010102		HUMALOG MIX 25 PEN 3ML INJ 100UNITS/ML
	06010102		HUMALOG MIX 50 PREFILLED PEN 3ML INJ 100UNITS/ML
	06010102		Humalog Mix25 Cartridges (3 Ml) 100 units/ml
	06010102		Humalog Mix25 Suspension For Injection 100 units/ml 3 ml pre-filled pen
	06.01.01.02.00		Humalog Mix25 100units/ml suspension for injection 10ml vials (Eli Lilly and Company Ltd)
fw11.	06010102		Humalog Mix25 100units/ml suspension for injection 3ml ca...
	06.01.01.02.00		Humalog Mix25 100units/ml suspension for injection 3ml cartridges (Eli Lilly and Company Ltd)
	06010102		Humalog Mix25 Kwikpen Suspension For Injection 100 units/ml 3 ml pre-filled pen
fw15.	06010102		Humalog Mix25 KwikPen 100units/ml suspension for injectio...
	06.01.01.02.00		Humalog Mix25 KwikPen 100units/ml suspension for injection 3ml pre-filled pen (Eli Lilly and Company Ltd)
	06.01.01.02.00		Humalog Mix25 Pen 100units/ml suspension for injection 3ml pre-filled pen (Eli Lilly and Company Ltd)
	06010102		Humalog Mix50 Injection (cartridges) 100 units/ml 3 ml cartridge
	06010102		Humalog Mix50 Suspension For Injection 100 units/ml 3 ml pre-filled pen
fw14.	06010102		Humalog Mix50 100units/ml suspension for injection 3ml ca...
	06.01.01.02.00		Humalog Mix50 100units/ml suspension for injection 3ml cartridges (Eli Lilly and Company Ltd)

	06010102		Humalog Mix50 Kwikpen Suspension For Injection 100 units/ml 3 ml pre-filled pen
fw16.	06010102		Humalog Mix50 KwikPen 100units/ml suspension for injectio...
	06.01.01.02.00		Humalog Mix50 KwikPen 100units/ml suspension for injection 3ml pre-filled pen (Eli Lilly and Company Ltd)
	06.01.01.02.00		Humalog Mix50 Pen 100units/ml suspension for injection 3ml pre-filled pen (Eli Lilly and Company Ltd)
	06.01.01.01.00		Humalog Pen 100units/ml solution for injection 3ml pre-filled pen (Eli Lilly and Company Ltd)
	06.01.01.02.00		HUMAN ACTRAPHANE injection 100 units/ml [NOVO]
	0601011N0BFAEAL		Human Actrapid Injection 100 units/ml
	0601011N0BFAPAM		Human Actrapid Preloaded Pen 100 units/ml
	06010101		HUMAN ACTRAPID 3ML PENFIL CARTRIDGES
	0601011N0BFAJAD		Human Actrapid Penfill Cartridges (1.5 Ml) 100 units/ml
	0601011N0BFAQAS		Human Actrapid Penfill Cartridges (3 Ml) 100 units/ml
	0601011N0BFAJAD		Human Actrapid Penfill 1.5ml INJ 100UNITS/ML
	06.01.01.01.00		Human Actrapid Penfill 100units/ml solution for injection 1.5ml cartridges (Novo Nordisk Ltd)
	06010101		human actrapid penfill 3ml INJ 100 units/ml
	06010101		Human Actrapid Penfill 3ml inj 100 units/ml
	06010101		Human Actrapid Penfill 3ml INJ 100UNITS/ML
	06010101		HUMAN ACTRAPID PENFILL 3ML INJ 100UNITS/ML
	06.01.01.02.00		HUMAN INITARD 50/50 injection 100 units/ml [NOVO]
	06010102		HUMAN INSULATARD 3ML PENFIL CARTRIDGES
	06010102		Human Insulatard Ge Penfill Cartridges (1.5 Ml) 100 units/ml
	0601012S0BDALAK		Human Insulatard Ge Penfill cartridges (3 ml) 100 units/ml
	0601012S0BDALAK		Human Insulatard Ge Penfill Cartridges (3 Ml) 100 units/ml
	0601012S0AAAHAH		Human Insulatard Ge Preloaded Pen 100 units/ml
	0601012S0BDAJAC		HUMAN INSULATARD GE VIAL 10ML INJ 100UNITS/ML
	06010102		Human Insulatard Penfill 1.5ml INJ 100UNITS/ML
	06010102		Human Insulatard Penfill 3ml INJ 100UNITS/ML
	06010102		HUMAN INSULATARD PENFILL 3ML INJ 100UNITS/ML
	0601012D0BCAZA0		Human Mixtard 20 Penfill cartridges (3 ml)
	06010102		HUMAN MIXTARD 20 PEN 3ML INJ 100UNITS/ML
	0601012D0BCAZA0		HUMAN MIXTARD 20 PEN 3ML INJ 100UNITS/ML
	0601012D0BCAZA0		HUMAN MIXTARD 20 PENFILL 3ML CARTRIDGE
	06010102		Human Mixtard 20 Penfill 3ml INJ 100UNITS/ML
	06010102		HUMAN MIXTARD 20 PENFILL 3ML INJ 100UNITS/ML
	06010102		Human Mixtard 30 Penfill Cartridges (1.5 Ml)
	0601012D0BCARA5		Human Mixtard 30 Preloaded pen
	06010102		HUMAN MIXTARD 30 100U/ML PENFILL 3ML

	0601012D0BCAWAQ		Human Mixtard 30 Ge Injection
	06010102		Human Mixtard 30 Ge Vial 10ml INJ 100UNITS/ML
	06010102		human mixtard 30 innolet
	06010102		HUMAN MIXTARD 30 INNOLET PREFILLED DEVICE 3ML INJ 100UNITS/ML
	0601012D0BCARA5		HUMAN MIXTARD 30 PEN
	06010102		Human Mixtard 30 Pen 3ml INJ 100UNITS/ML
	06010102		HUMAN MIXTARD 30 PEN 3ML INJ 100UNITS/ML
	0601012D0BCARA5		HUMAN MIXTARD 30 PEN 3ML INJ 100UNITS/ML
	0601012D0BCARA5		HUMAN MIXTARD 30 PEN 5 X 3ML
	06010102		Human Mixtard 30 Penfill 3ml INJ 100UNITS/ML
	06010102		HUMAN MIXTARD 30 PENFILL 3ML INJ 100UNITS/ML
	0601012D0BCBAA0		HUMAN MIXTARD 30 PENFILL 3ML INJ 100UNITS/ML
	0601012D0BCAUA0		Human Mixtard 40 Preloaded pen
	06010102		Human Mixtard 40 Penfill 3ml INJ 100UNITS/ML
	06.01.01.02.00		Human Mixtard 50 100units/ml suspension for injection 10ml vials (Novo Nordisk Ltd)
	06010102		Human Mixtard 50 Pen 3ml INJ 100UNITS/ML
	06010102		Human Mixtard 50 Penfill 3ml INJ 100UNITS/ML
	0601012G0BDABAE		Human Monotard (Novo) Injection 100 units/ml
	06010102		Human Monotard Vial 10ml INJ 100UNITS/ML
	06010102		HUMAN MONOTARD VIAL 10ML INJ 100UNITS/ML
	06.01.01.02.00		HUMAN PROTAPHANE injection 100 units/ml [NOVO]
	06.01.01.02.00		HUMAN PROTAPHANE PENFILL 100 units/ml [NOVO]
	0601011N0BFACAB		Human Velosulin Injection 100 u/ml
	06010101		Human Velosulin Vial 10ml INJ 100UNITS/ML
	06.01.01.04.00		HumaPen Ergo hypodermic insulin injection pen reusable for 3ml cartridge 1 unit dial up / range 1-60 units Burgundy (Eli Lilly and Company Ltd)
	06.01.01.04.00		HumaPen Luxura HD hypodermic insulin injection pen reusable for 3ml cartridge 0.5 unit dial up / range 1-30 units (Eli Lilly and Company Ltd)
	06.01.01.03.00		HumaPen Luxura hypodermic insulin injection pen reusable for 3ml cartridge 1 unit dial up / range 1-60 units Champagne (Eli Lilly and Company Ltd)
	06010103		Humapen Savvio Re-usable pen 3 ml 1-60 units Blue
	06010103		Humapen Savvio Re-usable pen 3 ml 1-60 units Graphite
	06010103		Humapen Savvio Re-usable pen 3 ml 1-60 units Green
	06010103		Humapen Savvio Re-usable pen 3 ml 1-60 units Pink
	06010103		Humapen Savvio Re-usable pen 3 ml 1-60 units Red
	06010103		Humapen Savvio Re-usable pen 3 ml 1-60 units Silver
	06.01.01.03.00		HumaPen Savvio hypodermic insulin injection pen reusable for 3ml cartridge 1 unit dial up / range 1-60 units Pink (Eli Lilly and Company Ltd)

	06.01.01.04.00		HumaPen Savvio hypodermic insulin injection pen reusable for 3ml cartridge 1 unit dial up / range 1-60 units Red (Eli Lilly and Company Ltd)
	0601012S0BGABAF		Humulin I Cartridges (1.5 Ml) 100 units/ml
	0601012S0BGADAL		Humulin I Cartridges (3 Ml) 100 units/ml
	0601012S0BGAAAE		Humulin I Injection 100 units/ml
	06.01.01.02.00		Humulin I 100units/ml suspension for injection 10ml vials (Eli Lilly and Company Ltd)
f25a.	06010102		Humulin I 100units/ml suspension for injection 10ml vials...
f25o.	06010102		Humulin I 100units/ml suspension for injection 3ml cartri...
	06.01.01.02.00		Humulin I 100units/ml suspension for injection 3ml cartridges (Eli Lilly and Company Ltd)
	0601012S0BGABAF		Humulin I Cartridge 1.5ml INJ 100UNITS/ML
	0601012S0BGABAF		HUMULIN I CARTRIDGE 1.5ML INJ 100UNITS/ML
	0601012S0BGADAL		Humulin I Cartridge 3ml INJ 100UNITS/ML
	0601012S0BGADAL		HUMULIN I CARTRIDGE 3ML INJ 100UNITS/ML
	06.01.01.02.00		HUMULIN I injection 100 units/ml [LILLY]
	06010102		Humulin I Kwikpen Suspension For Injection 100 units/ml 3 ml pre-filled pen
f25G.	06010102		Humulin I KwikPen 100units/ml suspension for injection 3m...
	06.01.01.02.00		Humulin I KwikPen 100units/ml suspension for injection 3ml pre-filled pen (Eli Lilly and Company Ltd)
	06.01.01.02.00		Humulin I Pen 100units/ml suspension for injection 3ml pre-filled pen (Eli Lilly and Company Ltd)
	06010102		Humulin I Prefilled Pen 3ml INJ 100UNITS/ML
	0601012S0BGAAAE		HUMULIN I VIAL 10ML INJ 100UNITS/ML
	0601012D0BBAQAY		Humulin M1 Cartridges (3 Ml)
	06.01.01.02.00		HUMULIN M1 injection 100 units/ml [LILLY]
	0601012D0BBARAZ		Humulin M2 Cartridges (3 Ml)
	06.01.01.02.00		Humulin M2 100units/ml suspension for injection 3ml cartridges (Eli Lilly and Company Ltd)
	0601012D0BBAFAL		HUMULIN M2 CARTRIDGE 1.5ML INJ 100UNITS/ML
	0601012D0BBARAZ		Humulin M2 Cartridge 3ml INJ 100UNITS/ML
	0601012D0BBARAZ		HUMULIN M2 CARTRIDGE 3ML INJ 100UNITS/ML
	0601012D0BBABAF		Humulin M2 Vial 10ml INJ 100UNITS/ML
	0601012D0BBASBA		Humulin M3 Cartridges (3 Ml)
	0601012D0BBACAG		Humulin M3 Suspension For Injection 100 units/ml 10 ml vial
	0601012D0BBASBA		Humulin M3 Suspension For Injection 100 units/ml 3 ml cartridge
	06010102		Humulin M3 Suspension For Injection 100 units/ml 3 ml pre-filled pen
f27d.	06010151		Humulin M3 100units/ml suspension for injection 10ml vial...

	06.01.01.02.00		Humulin M3 100units/ml suspension for injection 10ml vials (Eli Lilly and Company Ltd)
f27z.	06010102		Humulin M3 100units/ml suspension for injection 3ml cartr...
f27z.	06010151		Humulin M3 100units/ml suspension for injection 3ml cartr...
	06.01.01.02.00		Humulin M3 100units/ml suspension for injection 3ml cartridges (Eli Lilly and Company Ltd)
	0601012D0BBASBA		Humulin M3 Cartridge 3ml INJ 100UNITS/ML
	0601012D0BBASBA		HUMULIN M3 CARTRIDGE 3ML INJ 100UNITS/ML
	06.01.01.02.00		HUMULIN M3 injection 100 units/ml [LILLY]
	06010102		Humulin M3 Kwikpen Suspension For Injection 100 units/ml 3 ml pre-filled pen
f28E.	06010151		Humulin M3 KwikPen 100units/ml suspension for injection 3...
	06.01.01.02.00		Humulin M3 KwikPen 100units/ml suspension for injection 3ml pre-filled pen (Eli Lilly and Company Ltd)
	06.01.01.02.00		Humulin M3 Pen 100units/ml suspension for injection 3ml pre-filled pen (Eli Lilly and Company Ltd)
	06010102		Humulin M3 Prefilled Pen 3ml INJ 100UNITS/ML
	06010102		HUMULIN M3 PREFILLED PEN 3ML INJ 100UNITS/ML
	0601012D0BBACAG		Humulin M3 Vial 10ml INJ 100UNITS/ML
	0601012D0BBADAH		Humulin M4 Vial 10ml INJ 100UNITS/ML
	06.01.01.02.00		Humulin M5 100units/ml suspension for injection 10ml vials (Eli Lilly and Company Ltd)
	0601011N0BHABAI		Humulin S Cartridges (1.5 Ml) 100 units/ml
	0601011N0BHADAP		Humulin S Cartridges (3 Ml) 100 units/ml
	0601011N0BHAAAG		Humulin S Injection 100 units/ml
	06.01.01.01.00		Humulin S 100units/ml solution for injection 10ml vials (Eli Lilly and Company Ltd)
f12s.	06010101		Humulin S 100units/ml solution for injection 3ml cartridg...
	06.01.01.01.00		Humulin S 100units/ml solution for injection 3ml cartridges (Eli Lilly and Company Ltd)
	0601011N0BHABAI		Humulin S Cartridge 1.5ml INJ 100UNITS/ML
	0601011N0BHADAP		Humulin S Cartridge 3ml INJ 100UNITS/ML
	0601011N0BHADAP		HUMULIN S CARTRIDGE 3ML INJ 100UNITS/ML
	06.01.01.01.00		HUMULIN S injection 100 units/ml [LILLY]
	0601011N0BHAAAG		HUMULIN S VIAL 10ML INJ 100UNITS/ML
	06.01.01.02.00		Humulin Zn 100units/ml suspension for injection 10ml vials (Eli Lilly and Company Ltd)
f25v.	06010102		Hypurin Bovine Isophane 100units/ml suspension for inject...
	06.01.01.02.00		Hypurin Bovine Isophane 100units/ml suspension for injection 10ml vials (Wockhardt UK Ltd)
	06.01.01.02.00		HYPURIN BOVINE ISOPHANE injection 100 units/ml [CP PHARM]

	06.01.01.02.00		Hypurin Bovine Lente 100units/ml suspension for injection 10ml vials (Wockhardt UK Ltd)
	06.01.03.00.00		Hypurin Bovine Neutral 100units/ml solution for injection 10ml vials (Wockhardt UK Ltd)
f12H.	06010101		Hypurin Bovine Neutral 100units/ml solution for injection...
	06.01.01.01.00		HYPURIN BOVINE NEUTRAL injection 100 units/ml [CP PHARM]
	06.01.01.02.00		Hypurin Bovine Protamine Zinc 100units/ml suspension for injection 10ml vials (Wockhardt UK Ltd)
	06.01.01.02.00		Hypurin Porcine 30/70 Mix 100units/ml suspension for injection 10ml vials (Wockhardt UK Ltd)
	06.01.01.02.00		Hypurin Porcine 30/70 Mix 100units/ml suspension for injection 3ml cartridges (Wockhardt UK Ltd)
	06010102		Hypurin Porcine 30/70 Mix Cartridge 3ml INJ 100UNITS/ML
f25w.	06010102		Hypurin Porcine Isophane 100units/ml suspension for injec...
	06.01.01.02.00		Hypurin Porcine Isophane 100units/ml suspension for injection 10ml vials (Wockhardt UK Ltd)
	06.01.01.02.00		Hypurin Porcine Isophane 100units/ml suspension for injection 3ml cartridges (Wockhardt UK Ltd)
	06010102		Hypurin Porcine Isophane Cartridge 3ml INJ 100UNITS/ML
	06.01.01.02.00		HYPURIN PORCINE ISOPHANE injection 100 units/ml [WOCKHARDT]
f12I.	06010101		Hypurin Porcine Neutral 100units/ml solution for injectio...
	06.01.03.00.00		Hypurin Porcine Neutral 100units/ml solution for injection 10ml vials (Wockhardt UK Ltd)
	06.01.03.00.00		Hypurin Porcine Neutral 100units/ml solution for injection 3ml cartridges (Wockhardt UK Ltd)
	06010101		Hypurin Porcine Neutral Cartridge 3ml INJ 100UNITS/ML
	06.01.01.01.00		HYPURIN PORCINE NEUTRAL injection 100 units/ml [CP PHARM]
	06.01.01.03.00		Innovo hypodermic insulin injection pen reusable for 3ml cartridge 1 unit dial up / range 1-70 units Green (Novo Nordisk Ltd)
	06.01.01.04.00		Innovo hypodermic insulin injection pen reusable for 3ml cartridge 1 unit dial up / range 1-70 units Orange (Novo Nordisk Ltd)
	06.01.01.04.00		InsuJet needle free starter kit [EU PHAR BV]
	06010102		Insulatard Injection 100 units/ml 10 ml vial
f25p.	06010102		Insulatard 100units/ml suspension for injection 10ml vial...
	06.01.01.02.00		Insulatard 100units/ml suspension for injection 10ml vials (Novo Nordisk Ltd)
	06.01.01.02.00		Insulatard FlexPen 100units/ml suspension for injection (Novo Nordisk Ltd)
	06010102		Insulatard Flexpen Prefilled Pen 3ml INJ 100UNITS/ML
	06.01.01.02.00		INSULATARD ge injection 100 units/ml [NOVO]

	06010102		INSULATARD HUMAN (INSULIN) 3ML CARTRIDGE
	06.01.01.02.00		INSULATARD injection 100 units/ml [NOVO]
	06010102		Insulatard Innolet Suspension For Injection 100 units/ml 3 ml pre-filled pen
f25C.	06010102		Insulatard InnoLet 100units/ml suspension for injection 3...
	06.01.01.02.00		Insulatard InnoLet 100units/ml suspension for injection 3ml pre-filled pen (Novo Nordisk Ltd)
	06.01.01.02.00		INSULATARD INNOLET injection 100 units/ml [NOVO]
	06.01.01.02.00		Insulatard NovoLet 100units/ml suspension for injection (Novo Nordisk Ltd)
	06010102		Insulatard Penfill Injection 100 units/ml 3 ml cartridge
	06.01.01.02.00		INSULATARD PENFILL 100 units/ml [NOVO]
f25x.	06010102		Insulatard Penfill 100units/ml suspension for injection 3...
	06.01.01.02.00		Insulatard Penfill 100units/ml suspension for injection 3ml cartridges (Novo Nordisk Ltd)
	06010102		Insulatard Penfill 3ml INJ 100UNITS/ML
	06.01.01.01.00		Insulin aspart 100units/ml solution for injection 10ml vials
	06.01.01.01.00		Insulin aspart 100units/ml solution for injection 3ml cartridges
	06.01.01.01.00		Insulin aspart 100units/ml solution for injection 3ml pre-filled disposable devices
	06.01.01.02.00		Insulin aspart biphasic 30/70 100units/ml suspension for injection 3ml cartridges
	06.01.01.02.00		Insulin aspart biphasic 30/70 100units/ml suspension for injection 3ml pre-filled disposable devices
	06.01.01.01.00		insulin aspart human pyr injection 100 units/ml
	06.01.01.02.00		insulin biphasic injection 100 units/ml
	06.01.01.02.00		insulin biphasic isophane human prb injection 30:70; 100 units/ml
	06.01.01.02.00		insulin biphasic isophane human pyr injection 30:70; 100 units/ml
	06.01.01.02.00		insulin biphasic lispro human prb injection 25:75; 100 units/ml
	06.01.01.02.00		insulin biphasic lispro human prb injection 50:50; 100 units/ml
	06.01.01.02.00		Insulin degludec 100units/ml solution for injection 3ml pre-filled disposable devices
	06010102		Insulin degludec 200units/ml solution for injection 3ml p...
	06.01.01.02.00		Insulin degludec 200units/ml solution for injection 3ml pre-filled disposable devices
	06010102		Insulin Detemir Solution for injection 100 units/ml 3 ml pre-filled pen
	06.01.01.02.00		Insulin detemir 100units/ml solution for injection 3ml cartridges
	06.01.01.02.00		Insulin detemir 100units/ml solution for injection 3ml pre-filled disposable devices

	06.01.01.02.00		insulin detemir injection solution 100 units/ml
	06010102		Insulin Glargine Injection 100 units/ml 3 ml cartridge
	06010102		Insulin Glargine Pre-filled Disposable Pen 100 units/ml 3 ml pen
	06010102		INSULIN GLARGINE 100UNITS/ML 3ML CARTIDGES 100UNITS/ML
	06.01.01.02.00		Insulin glargine 100units/ml solution for injection 10ml vials
f291.	06010102		Insulin glargine 100units/ml solution for injection 3ml c...
	06.01.01.02.00		Insulin glargine 100units/ml solution for injection 3ml cartridges
	06.01.01.02.00		Insulin glargine 100units/ml solution for injection 3ml pre-filled disposable devices
	06.01.01.02.00		Insulin glargine 300units/ml solution for injection 1.5ml pre-filled disposable devices
	06.01.01.02.00		insulin glargine injection 100 units/ml
	06.01.01.01.00		Insulin glulisine 100units/ml solution for injection 3ml cartridges
	06.01.01.01.00		Insulin glulisine 100units/ml solution for injection 3ml pre-filled disposable devices
	06.01.01.02.00		Insulin isophane biphasic human 30/70 100units/ml suspension for injection 10ml vials
	06.01.01.02.00		Insulin isophane biphasic human 30/70 100units/ml suspension for injection 3ml cartridges
	06.01.01.02.00		Insulin isophane biphasic human 30/70 100units/ml suspension for injection 3ml pre-filled disposable devices
	06.01.01.02.00		insulin isophane biphasic human crb injection 25:75; 100 units/ml
	06.01.01.02.00		Insulin isophane biphasic porcine 30/70 100units/ml suspension for injection 3ml cartridges
	06.01.01.02.00		Insulin isophane human 100units/ml suspension for injection 1.5ml cartridges
	06.01.01.02.00		Insulin isophane human 100units/ml suspension for injection 3ml cartridges
	06.01.01.02.00		insulin isophane human emp injection 100 units/ml
	06.01.01.02.00		insulin isophane human injection 100 units/ml
	06.01.01.02.00		insulin isophane human prb injection 100 units/ml
	06.01.01.02.00		Insulin isophane porcine 100units/ml suspension for injection 3ml cartridges
	06.01.01.02.00		insulin isophane porcine injection 100 units/ml
	06.01.01.01.00		Insulin lispro 100units/ml solution for injection 10ml vials
	06.01.01.01.00		Insulin lispro 100units/ml solution for injection 3ml cartridges
	06.01.01.01.00		Insulin lispro 100units/ml solution for injection 3ml pre-filled disposable devices
	06.01.01.02.00		Insulin lispro biphasic 25/75 100units/ml suspension for injection 3ml cartridges
	06.01.01.01.00		insulin lispro human prb injection 100 units/ml
	06.01.03.00.00		insulin neutral human injection 100 units/ml

	06.01.01.01.00		insulin soluble bovine injection 100 units/ml
	06.01.03.00.00		Insulin soluble human 100units/ml solution for injection 10ml vials
	06.01.03.00.00		Insulin soluble human 100units/ml solution for injection 3ml cartridges
	06.01.01.01.00		insulin soluble human prb injection 100 units/ml
	06.01.01.01.00		insulin soluble human pyr injection 100 units/ml
	06010103		INSULIN SYRINGE 1 MLDISPOSABLE
	06010103		Insulin Syringe-P42 1ml disposable
	06010103		INSULIN SYRINGES 1ML
	06.01.01.02.00		Insuman Basal 100units/ml suspension for injection 3ml cartridges (Sanofi)
	06.01.01.02.00		Insuman Basal 100units/ml suspension for injection 3ml pre-filled SoloStar pen (Sanofi)
	06010102		Insuman Basal Solostar Pre-filled Disposable Pen 100 units/ml 3 ml pen
	06010102		Insuman Comb 15 Injection (cartridges) 100 units/ml 3 ml cartridge
f286.	06010151		Insuman Comb 15 100units/ml suspension for injection 3ml ...
	06.01.01.02.00		Insuman Comb 15 100units/ml suspension for injection 3ml cartridges (Sanofi)
	06010102		Insuman Comb 15 Optiset Pre-filled Disposable Pen 100 units/ml 3 ml pen
	06010102		INSUMAN COMB 15 OPTISET PRE LOADED DISPOSABLE PEN 3ML INJ 100UNITS/ML
	06.01.01.02.00		Insuman Comb 25 100units/ml suspension for injection 3ml cartridges (Sanofi)
	06.01.01.02.00		Insuman Comb 25 100units/ml suspension for injection 3ml pre-filled SoloStar pen (Sanofi)
	06010102		INSUMAN COMB 25 CARTRIDGE 3ML INJ 100UNITS/ML
	06010102		Insuman Comb 50 Injection (cartridges) 100 units/ml 3 ml cartridge
f28C.	06010151		Insuman Comb 50 100units/ml suspension for injection 3ml ...
	06.01.01.02.00		Insuman Comb 50 100units/ml suspension for injection 3ml cartridges (Sanofi)
	06.01.01.01.00		Insuman Rapid 100units/ml solution for injection 3ml pre-filled OptiSet pen (Sanofi)
	06.01.01.03.00		Insupen hypodermic insulin needles for pre-filled / reusable pen injectors screw on 4mm/32gauge (Spirit Healthcare Ltd)
	06.01.01.03.00		Insupen hypodermic insulin needles for pre-filled / reusable pen injectors screw on 4mm/33gauge (Spirit Healthcare Ltd)
	06.01.01.03.00		Insupen hypodermic insulin needles for pre-filled / reusable pen injectors screw on 6mm/31gauge (Spirit Healthcare Ltd)

	06.01.01.03.00		Insupen hypodermic insulin needles for pre-filled / reusable pen injectors screw on 8mm/31gauge (Spirit Healthcare Ltd)
	06.01.01.03.00		Insupen hypodermic insulin needles for pre-filled / reusable pen injectors screw on 8mm/32gauge (Spirit Healthcare Ltd)
	06.01.02.03.00		Invokana 100mg tablets (Janssen-Cilag Ltd)
	06.01.02.03.00		Invokana 300mg tablets (Janssen-Cilag Ltd)
	06.01.01.02.00		isophane insulin injection 100 units/ml
	06010203		Janumet Tablets 50 mg + 1000 mg
fte1.	06010203		Janumet 50mg/1000mg tablets (Merck Sharp & Dohme Ltd)
	06.01.02.03.00		Janumet 50mg/1000mg tablets (Merck Sharp & Dohme Ltd)
	06010203		Januvia Tablets 100 mg
ft81.	06010203		Januvia 100mg tablets (Merck Sharp & Dohme Ltd)
	06.01.02.03.00		Januvia 100mg tablets (Merck Sharp & Dohme Ltd)
	06010203		JANUVIA TABLETS 100MG
	06010203		Januvia TABS 100MG
ftg3.	06010203		Jentadueto 2.5mg/1000mg tablets (Boehringer Ingelheim Ltd)
	06.01.02.02.00		Jentadueto 2.5mg/1000mg tablets (Boehringer Ingelheim Ltd)
	06.01.01.03.00		Kendall Magellan hypodermic U100 insulin syringe sterile single use / single patient use 0.3ml with 8mm safety needle 0.3mm/30gauge (Covidien (UK) Commercial Ltd)
	06.01.01.03.00		Kendall Magellan hypodermic U100 insulin syringe sterile single use / single patient use 0.5ml with 8mm safety needle 0.3mm/30gauge (Covidien (UK) Commercial Ltd)
	06010102		Lantus Injection 100 units/ml 10 ml vial
	06010102		Lantus Injection 100 units/ml 3 ml cartridge
f295.	06010102		Lantus 100units/ml solution for injection 10ml vials (San...
	06.01.01.02.00		Lantus 100units/ml solution for injection 10ml vials (Sanofi)
	06.01.01.02.00		Lantus 100units/ml solution for injection 3ml cartridges (Sanofi)
f294.	06010102		Lantus 100units/ml solution for injection 3ml cartridges ...
	06.01.01.02.00		Lantus 100units/ml solution for injection 3ml OptiClik cartridges (Sanofi)
f298.	06010102		Lantus 100units/ml solution for injection 3ml pre-filled ...
	06.01.01.02.00		Lantus 100units/ml solution for injection 3ml pre-filled OptiSet pen (Sanofi)
	06.01.01.02.00		Lantus 100units/ml solution for injection 3ml pre-filled SoloStar pen (Sanofi)
	06010102		Lantus Cartridge 3ml INJ 100UNITS/ML
	06010102		LANTUS CARTRIDGE 3ML INJ 100UNITS/ML
f294.	06010102		LANTUS CARTRIDGE inj soln 100 units/ml

	06010102		lantus Glargine 3ml cartridge
	06.01.01.02.00		LANTUS injection 100 units/ml [AVENTIS]
	06010102		Lantus Insulin 3ml cartridge
	06010102		LANTUS INSULIN 3ML CARTRIDGES
	06010102		LANTUS INSULIN 3ML OPTISET
	06010102		LANTUS INSULIN CARTRIDGES 5 X 3 ML
	06010102		Lantus insulin INJ 3ml cartridge
	06010102		LANTUS INSULIN Penfill Cartiridges 3ml
	06010102		LANTUS LANTUS INSULIN 5 CART 3ML
	06010102		Lantus Opticlick Cartridge 3ml INJ 100UNITS/ML
	06010102		Lantus Opticlik Injection 100 units/ml 3 ml cartridge
	06010102		Lantus OptiSet
	06010102		Lantus Optiset Pre-filled Disposable Pen 100 units/ml 3 ml pen
	06010102		LANTUS OPTISET 3 ML PEN
	06010102		Lantus Optiset Prefilled Disposable Pen 3ml INJ 100UNITS/ML
	06010102		LANTUS OPTISET PREFILLED DISPOSABLE PEN 3ML INJ 100UNITS/ML
	06010102		Lantus Solostar Pre-filled Disposable Pen 100 units/ml 3 ml pen
	06010102		lantus solostar disposable pen pre filled syringe 100units per ml
f298.	06010102		LANTUS SOLOSTAR inj soln 100 units/ml
	06010102		Lantus Solostar Pen
	06010102		Lantus Solostar Prefilled Disposable Pen 3ml INJ 100UNITS/ML
	06010102		LANTUS SOLOSTAR PREFILLED DISPOSABLE PEN 3ML INJ 100UNITS/ML
	06010102		Lantus Vial 10ml INJ 100UNITS/ML
	06010102		LEVEMIR 3ML FLEXPEN
	06010102		Levemir Flexpen Solution for injection 100 units/ml 3 ml pre-filled pen
f2A2.	06010102		Levemir FlexPen 100units/ml solution for injection 3ml pr...
	06.01.01.02.00		Levemir FlexPen 100units/ml solution for injection 3ml pre-filled pen (Novo Nordisk Ltd)
	06010102		Levemir Flexpen 3ml Insulin
	06010102		Levemir Flexpen Prefilled Pen 3ml INJ 100UNITS/ML
	06010102		LEVEMIR FLEXPEN PREFILLED PEN 3ML INJ 100UNITS/ML
f2A3.	06010102		Levemir InnoLet 100units/ml solution for injection 3ml pr...
	06.01.01.02.00		Levemir InnoLet 100units/ml solution for injection 3ml pre-filled pen (Novo Nordisk Ltd)
	06010102		LEVEMIR NOVO NORDISK FLEXIPEN INJ 3 ML

	06010102		Levemir Penfill Injection (cartridges) 100 units/ml 3 ml cartridge
	06010102		Levemir Penfill Injection (Cartridges) 100 units/ml 3 ml cartridge
f2A1.	06010102		Levemir Penfill 100units/ml solution for injection 3ml ca...
	06.01.01.02.00		Levemir Penfill 100units/ml solution for injection 3ml cartridges (Novo Nordisk Ltd)
	06010102		Levemir Penfill 3ml INJ 100UNITS/ML
	06010102		LEVEMIR PENFILL 3ML INJ 100UNITS/ML
f2A1.	06010102		LEVEMIR PENFILL inj soln 100 units/ml
	06010203		Linagliptin Tablets 5 mg
	06.01.02.03.00		Linagliptin 2.5mg / Metformin 1g tablets
	06.01.02.02.00		Linagliptin 2.5mg / Metformin 850mg tablets
	06010203		Linagliptin 2.5mg / Metformin 850mg tablets
ftf2.	06010203		Linagliptin 5mg tablets
	06.01.02.03.00		Linagliptin 5mg tablets
	06010203		Liraglutide Solution for injection in pre-filled pen 6 mg/ml 3 ml pen
ftc2.	06010203		Liraglutide 6mg/ml solution for injection 3ml pre-filled ...
	06.01.02.03.00		Liraglutide 6mg/ml solution for injection 3ml pre-filled disposable devices
	06010203		Liraglutide Prefilled Pen 3ml INJ 6MG/ML
	06010203		Lixisenatide Solution for injection 10 micrograms/0.2 ml 3 ml device
	06010203		Lixisenatide Solution for injection 20 micrograms/0.2 ml 3 ml device
ftj2.	06010203		Lixisenatide 10micrograms/0.2ml solution for injection 3m...
ftj6.	06010203		Lixisenatide 10micrograms/0.2ml solution for injection 3m...
	06.01.02.03.00		Lixisenatide 10micrograms/0.2ml solution for injection 3ml pre-filled disposable devices
	06.01.02.03.00		Lixisenatide 10micrograms/0.2ml solution for injection 3ml pre-filled disposable devices and Lixisenatide 20micrograms/0.2ml solution for injection 3ml pre-filled disposable devices
ftj4.	06010203		Lixisenatide 20micrograms/0.2ml solution for injection 3m...
	06.01.02.03.00		Lixisenatide 20micrograms/0.2ml solution for injection 3ml pre-filled disposable devices
ftj1.	06010203		Lyxumia 10micrograms/0.2ml solution for injection 3ml pre...
ftj5.	06010203		Lyxumia 10micrograms/0.2ml solution for injection 3ml pre...
	06.01.02.03.00		Lyxumia 10micrograms/0.2ml solution for injection 3ml pre-filled pen (Sanofi)
	06.01.02.03.00		Lyxumia 10micrograms/20micrograms treatment initiation pack (Sanofi)

ftj3.	06010203		Lyxumia 20micrograms/0.2ml solution for injection 3ml pre...
	06.01.02.03.00		Lyxumia 20micrograms/0.2ml solution for injection 3ml pre-filled pen (Sanofi)
	06.01.02.02.00		Metabet SR 1000mg tablets (Morningside Healthcare Ltd)
	06.01.02.02.00		Metabet SR 500mg tablets (Actavis UK Ltd)
f41E.	06010202		Metabet SR 500mg tablets (Morningside Healthcare Ltd)
	06.01.02.02.00		Metabet SR 500mg tablets (Morningside Healthcare Ltd)
	06.01.02.02.00		metformin (roi) tablets 1000mg
ftez.	06010203		Metformin 1g / Sitagliptin 50mg tablets
	06.01.02.02.00		Metformin 1g / Sitagliptin 50mg tablets
f41u.	06010202		Metformin 1g modified-release tablets
	06.01.02.02.00		Metformin 1g modified-release tablets
	06.01.02.02.00		Metformin 1g modified-release tablets (Actavis UK Ltd)
	06.01.02.02.00		Metformin 1g oral powder sachets sugar free
f41..	06010202		Metformin 1g/5ml oral solution sugar free
f41x.	06010202		Metformin 500mg modified-release tablets
	06.01.02.02.00		Metformin 500mg modified-release tablets
	06.01.02.02.00		Metformin 500mg modified-release tablets (Actavis UK Ltd)
	06.01.02.02.00		Metformin 500mg modified-release tablets (Almus Pharmaceuticals Ltd)
f41t.	06010202		Metformin 500mg oral powder sachets sugar free
	06.01.02.02.00		Metformin 500mg oral powder sachets sugar free
f41y.	06010202		Metformin 500mg tablets
	06.01.02.02.00		Metformin 500mg tablets
	0601022B0AAABAB		Metformin 500mg tablets
	06.01.02.02.00		Metformin 500mg tablets (A A H Pharmaceuticals Ltd)
	0601022B0AAABAB		Metformin 500mg tablets (A A H Pharmaceuticals Ltd)
	06.01.02.02.00		Metformin 500mg tablets (Actavis UK Ltd)
	06.01.02.02.00		Metformin 500mg tablets (Almus Pharmaceuticals Ltd)
	06.01.02.02.00		Metformin 500mg tablets (Kent Pharmaceuticals Ltd)
	06.01.02.02.00		Metformin 500mg tablets (Mylan Ltd)
	06.01.02.02.00		Metformin 500mg tablets (Sandoz Ltd)
	06.01.02.02.00		Metformin 500mg tablets (Teva UK Ltd)
	06.01.02.02.00		Metformin 500mg tablets (Wockhardt UK Ltd)
	06.01.02.02.00		Metformin 500mg tablets (Zentiva)
f41w.	06010202		Metformin 500mg/5ml oral solution sugar free
	06.01.02.02.00		Metformin 500mg/5ml oral solution sugar free
	06.01.02.02.00		Metformin 500mg/5ml oral solution sugar free (A A H Pharmaceuticals Ltd)
	06.01.02.02.00		Metformin 500mg/5ml oral solution sugar free (Rosemont Pharmaceuticals Ltd)
f41v.	06010202		Metformin 750mg modified-release tablets

	06.01.02.02.00		Metformin 750mg modified-release tablets
f41z.	06010202		Metformin 850mg tablets
	06.01.02.02.00		Metformin 850mg tablets
	06.01.02.02.00		Metformin 850mg tablets (A A H Pharmaceuticals Ltd)
	06.01.02.02.00		Metformin 850mg tablets (Actavis UK Ltd)
	06.01.02.02.00		Metformin 850mg tablets (Kent Pharmaceuticals Ltd)
	06.01.02.02.00		Metformin 850mg tablets (Teva UK Ltd)
	06.01.02.02.00		Metformin 850mg tablets (Wockhardt UK Ltd)
	06010203		Metformin And Linagliptin Tablets 1000 mg + 2.5 mg
	06010203		Metformin And Sitagliptin Tablets 1000 mg + 50 mg
	06010203		Metformin And Vildagliptin Tablets 1 gram + 50 mg
	06010203		Metformin And Vildagliptin Tablets 850 mg + 50 mg
	06010202		Metformin Hydrochloride M/R tablets 1 gram
	06010202		Metformin Hydrochloride M/R tablets 500 mg
	06010202		Metformin Hydrochloride M/R tablets 750 mg
	06010202		Metformin Hydrochloride Oral solution 500 mg/5 ml
	06010202		Metformin Hydrochloride Oral solution Sugar Free 500 mg/5 ml
	06010202		Metformin Hydrochloride Sugar free suspension 500 mg/5 ml
	0601022B0AAABAB		Metformin Hydrochloride Tablets 500 mg
	0601022B0AAADAD		Metformin Hydrochloride Tablets 850 mg
	06010202		METFORMIN HYDROCHLORIDE MR TABLETS 500MG
	06010202		Metformin Hydrochloride Mr TABS 1G
	06010202		Metformin Hydrochloride Mr TABS 500MG
	06010202		METFORMIN HYDROCHLORIDE SF ORAL SOLN 500MG/5ML
	06010202		Metformin Hydrochloride Sf Powder For Oral Solution SACH 500MG
f41y.	06010202		Metformin Hydrochloride Tablets 500mg
	0601022B0AAABAB		Metformin Hydrochloride TABLETS 500MG
	0601022B0AAABAB		METFORMIN HYDROCHLORIDE TABLETS 500MG
	0601022B0AAADAD		METFORMIN HYDROCHLORIDE TABLETS 850MG
	0601022B0AAABAB		Metformin Hydrochloride TABS 500MG
	0601022B0AAADAD		Metformin Hydrochloride TABS 850MG
f41x.	06010202		METFORMIN mr tab 500mg
	06.01.02.02.00		metformin oral suspension 100mg/ml
	0601022B0AAABAB		METFORMIN TABLETS 500MG
	0601022B0AAABAB		METFORMIN tablets TAB 500mg-P42 0
f41y.	06010202		METFORMIN tabs 500mg
	06.01.02.01.00		Minodiab 5mg tablets (Pfizer Ltd)
	06.01.01.02.00		Mixtard 10 NovoLet 100units/ml suspension for injection (Novo Nordisk Ltd)

	06.01.01.02.00		MIXTARD 10 PENFILL 100 units/ml [NOVO]
	06.01.01.02.00		Mixtard 10 Penfill 100units/ml suspension for injection 3ml cartridges (Novo Nordisk Ltd)
	06010102		Mixtard 10 Penfill 3ml INJ 100UNITS/ML
	06010102		Mixtard 20 Novolet Pre-filled Disposable Injection 100 units/ml 3 ml device
	06.01.01.02.00		Mixtard 20 NovoLet 100units/ml suspension for injection (Novo Nordisk Ltd)
	06.01.01.02.00		MIXTARD 20 PENFILL 100 units/ml [NOVO]
	06.01.01.02.00		Mixtard 20 Penfill 100units/ml suspension for injection 3ml cartridges (Novo Nordisk Ltd)
	06010102		Mixtard 20 Penfill 3ml INJ 100UNITS/ML
	06010102		MIXTARD 20 PENFILL 3ML INJ 100UNITS/ML
	06.01.01.02.00		Mixtard 30 100units/ml suspension for injection 10ml vials (Novo Nordisk Ltd)
	06.01.01.02.00		MIXTARD 30 ge injection 100 units/ml [NOVO]
	06010102		Mixtard 30 Innolet Suspension For Injection 100 units/ml 3 ml pre-filled pen
	06.01.01.02.00		Mixtard 30 InnoLet 100units/ml suspension for injection 3ml pre-filled pen (Novo Nordisk Ltd)
	06010102		Mixtard 30 Innolet Prefilled Device 3ml INJ 100UNITS/ML
	06010102		MIXTARD 30 INNOLET PREFILLED DEVICE 3ML INJ 100UNITS/ML
	06010102		Mixtard 30 Novolet Pre-filled Disposable Injection 100 units/ml 3 ml device
	06.01.01.02.00		Mixtard 30 NovoLet 100units/ml suspension for injection (Novo Nordisk Ltd)
	06010102		Mixtard 30 Novolet Pen 3ml INJ 100UNITS/ML
	06010102		Mixtard 30 Penfill Injection 100 units/ml 3 ml cartridge
	06.01.01.02.00		MIXTARD 30 PENFILL 100 units/ml [NOVO]
	06.01.01.02.00		Mixtard 30 Penfill 100units/ml suspension for injection 3ml cartridges (Novo Nordisk Ltd)
	06010102		Mixtard 30 Penfill 3ml INJ 100UNITS/ML
	06010102		MIXTARD 30 PENFILL 3ML INJ 100UNITS/ML
	06010102		Mixtard 30 Vial 10ml INJ 100UNITS/ML
	06.01.01.02.00		MIXTARD 30/70 injection 100 units/ml [NOVO]
	06010102		Mixtard 40 Novolet Pen 3ml INJ 100UNITS/ML
	06.01.01.02.00		MIXTARD 40 PENFILL 100 units/ml [NOVO]
	06.01.01.02.00		Mixtard 40 Penfill 100units/ml suspension for injection 3ml cartridges (Novo Nordisk Ltd)
	06010102		Mixtard 40 Penfill 3ml INJ 100UNITS/ML
	06010102		MIXTARD 40 PENFILL 3ML INJ 100UNITS/ML
	06.01.01.02.00		MIXTARD 50 PENFILL 100 units/ml [NOVO]
	06.01.01.02.00		Mixtard 50 Penfill 100units/ml suspension for injection 3ml cartridges (Novo Nordisk Ltd)
	06.01.01.03.00		Monoject hypodermic needle sterile single use 0.4mm/27gauge 12mm (Covidien (UK) Commercial Ltd)

	06.01.01.04.00		Monoject hypodermic U100 insulin syringe sterile single use / single patient use 0.5ml with 12mm needle 0.36mm/28gauge (Covidien (UK) Commercial Ltd)
	06.01.01.03.00		Monoject hypodermic U100 insulin syringe sterile single use / single patient use 1ml with 12mm needle 0.36mm/28gauge (Covidien (UK) Commercial Ltd)
	06.01.01.02.00		Monotard 100units/ml suspension for injection 10ml vials (Novo Nordisk Ltd)
	06.01.01.02.00		MONOTARD MC injection 100 units/ml [NOVO]
	06.01.02.03.00		Nateglinide 120mg tablets
	06.01.02.03.00		Nateglinide 180mg tablets
	06.01.02.03.00		Nateglinide 60mg tablets
	06010203		Nateglinide TABS 120MG
	06010203		Nateglinide TABS 180MG
	06010203		Nateglinide TABS 60MG
	06010102		NOVOMIX 30 FLEXPEN
	06010102		Novomix 30 Flexpen Suspension For Injection 100 units/ml 3 ml pre-filled pen
fw22.	06010102		NovoMix 30 FlexPen 100units/ml suspension for injection 3...
fw22.	06010151		NovoMix 30 FlexPen 100units/ml suspension for injection 3...
	06.01.01.02.00		NovoMix 30 FlexPen 100units/ml suspension for injection 3ml pre-filled pen (Novo Nordisk Ltd)
	06010102		Novomix 30 Flexpen Prefilled Pen 3ml INJ 100UNITS/ML
	06010102		NOVOMIX 30 FLEXPEN PREFILLED PEN 3ML INJ 100UNITS/ML
	06.01.01.02.00		NOVOMIX 30 injection 30:70; 100 units/ml [NOVO]
	06010102		Novomix 30 Penfill Injection (cartridges) 100 units/ml 3 ml cartridge
fw21.	06010102		NovoMix 30 Penfill 100units/ml suspension for injection 3...
fw21.	06010151		NovoMix 30 Penfill 100units/ml suspension for injection 3...
	06.01.01.02.00		NovoMix 30 Penfill 100units/ml suspension for injection 3ml cartridges (Novo Nordisk Ltd)
	06010102		Novomix 30 Penfill 3ml INJ 100UNITS/ML
	06010102		NOVOMIX 30 PENFILL 3ML INJ 100UNITS/ML
	06.01.02.03.00		NovoNorm 1mg tablets (Novo Nordisk Ltd)
	06.01.02.03.00		NovoNorm 2mg tablets (Novo Nordisk Ltd)
	06.01.02.03.00		NovoNorm 500microgram tablets (Novo Nordisk Ltd)
	06.01.01.03.00		NovoPen 3 Demi hypodermic insulin injection pen reusable for 3ml cartridge 0.5 unit dial up / range 1-35 units (Novo Nordisk Ltd)
	06.01.01.04.00		NovoPen 3 Fun hypodermic insulin injection pen reusable for 3ml cartridge 1 unit dial up / range 2-70 units Red (Novo Nordisk Ltd)

pm1y.	06010103		NovoPen 4 hypodermic insulin injection pen reusable for 3...
	06.01.01.04.00		NovoPen 5 hypodermic insulin injection pen reusable for 3ml cartridge 1 unit dial up / range 1-60 units Blue (Novo Nordisk Ltd)
	06.01.01.04.00		NovoPen CLASSIC injection device [NOVO]
	06.01.01.03.00		NovoPen Echo hypodermic insulin injection pen reusable for 3ml cartridge 0.5 unit dial up / range 0.5-30 units Blue (Novo Nordisk Ltd)
	06.01.01.01.00		NOVOPEN injection device 100 units/ml [NOVO]
	06010101		Novorapid Injection 100 units/ml 10 ml vial
f141.	06010101		NovoRapid 100units/ml solution for injection 10ml vials (...
	06.01.01.01.00		NovoRapid 100units/ml solution for injection 10ml vials (Novo Nordisk Ltd)
	06010101		Novorapid Flexpen Pre-filled Disposable Pen 100 units/ml 3 ml pen
	06010101		Novorapid Flexpen Solution for injection 100 units/ml 3 ml pre-filled pen
f144.	06010101		NovoRapid FlexPen 100units/ml solution for injection 3ml ...
	06.01.01.01.00		NovoRapid FlexPen 100units/ml solution for injection 3ml pre-filled pen (Novo Nordisk Ltd)
f144.	06010101		NOVORAPID FLEXPEN inj soln 100 units/ml
	06010101		Novorapid Flexpen Prefilled Pen 3ml INJ 100UNITS/ML
	06010101		NOVORAPID FLEXPEN PREFILLED PEN 3ML INJ 100UNITS/ML
	06010101		Novorapid Flextouch Solution for injection 100 units/ml 3 ml pre-filled pen
f145.	06010101		NovoRapid FlexTouch 100units/ml solution for injection 3m...
	06.01.01.01.00		NovoRapid FlexTouch 100units/ml solution for injection 3ml pre-filled pen (Novo Nordisk Ltd)
	06010101		Novorapid Novolet Prefilled syringes 100 units/ml
	06.01.01.01.00		NovoRapid Novolet 100units/ml solution for injection (Novo Nordisk Ltd)
	06010101		Novorapid Novolet 3ml INJ 100UNITS/ML
	06010101		NOVORAPID PENFIL 3ML INJ 100U/ML 5
	06010101		NOVORAPID PENFILL
	06010101		Novorapid Penfill Cartridges (3 Ml) 100 units/ml
f143.	06010101		NovoRapid Penfill 100units/ml solution for injection 3ml ...
	06.01.01.01.00		NovoRapid Penfill 100units/ml solution for injection 3ml cartridges (Novo Nordisk Ltd)
	06010101		Novorapid Penfill 3ml INJ 100UNITS/ML
	06010101		NOVORAPID PENFILL 3ML INJ 100UNITS/ML
f143.	06010101		NOVORAPID PENFILL inj soln 100 units/ml

	06.01.01.01.00		NovoRapid PumpCart 100units/ml solution for injection 1.6ml cartridges (Novo Nordisk Ltd)
	06010101		Novorapid Vial 10ml INJ 100UNITS/ML
	06010101		NOVORAPID VIAL 10ML INJ 100UNITS/ML
	06.01.02.03.00		Onglyza 2.5mg tablets (AstraZeneca UK Ltd)
	06.01.01.04.00		OptiClik hypodermic insulin injection pen reusable for 3ml cartridge 1 unit dial up / range 1-80 units Blue (Sanofi)
	06.01.01.03.00		OptiClik hypodermic insulin injection pen reusable for 3ml cartridge 1 unit dial up / range 1-80 units Grey (Sanofi)
	06.01.01.02.00		PENMIX 30/70 injection 100 units/ml [NOVO]
	06.01.01.02.00		PENMIX 30/70 PENFILL injection 100 units/ml [NOVO]
ft7..	06010203		Pioglitazone 15mg / Metformin 850mg tablets
	06.01.02.02.00		Pioglitazone 15mg / Metformin 850mg tablets
ft5z.	06010203		Pioglitazone 15mg tablets
	06.01.02.03.00		Pioglitazone 15mg tablets
	06010203		Pioglitazone 15mg tablets
	06.01.02.03.00		Pioglitazone 15mg tablets (A A H Pharmaceuticals Ltd)
	06.01.02.03.00		Pioglitazone 15mg tablets (Actavis UK Ltd)
	06.01.02.03.00		Pioglitazone 15mg tablets (Teva UK Ltd)
ft5y.	06010203		Pioglitazone 30mg tablets
	06.01.02.03.00		Pioglitazone 30mg tablets
	06010203		Pioglitazone 30mg tablets
	06010203		PIOGLITAZONE 30MG TABLETS
	06.01.02.03.00		Pioglitazone 30mg tablets (A A H Pharmaceuticals Ltd)
	06.01.02.03.00		Pioglitazone 30mg tablets (Actavis UK Ltd)
ft5x.	06010203		Pioglitazone 45mg tablets
	06.01.02.03.00		Pioglitazone 45mg tablets
	06010203		Pioglitazone And Metformin Tablets 15 mg + 850 mg
	06010203		Pioglitazone Hydrochloride Tablets 15 mg
	06010203		Pioglitazone Hydrochloride Tablets 30 mg
	06010203		Pioglitazone Hydrochloride Tablets 45 mg
	06010203		Pioglitazone TABLETS 15MG
	06010203		PIOGLITAZONE TABLETS 15MG
	06010203		Pioglitazone TABLETS 30MG
	06010203		PIOGLITAZONE TABLETS 30MG
	06010203		PIOGLITAZONE TABLETS 45MG
ft5z.	06010203		PIOGLITAZONE tabs 15mg
	06010203		Pioglitazone TABS 15MG
	06010203		Pioglitazone TABS 30MG
ft5x.	06010203		PIOGLITAZONE tabs 45mg
	06010203		Pioglitazone TABS 45MG
	06010101		Pork Actrapid Injection 100 units/ml 10 ml vial

	06.01.03.00.00		Pork Actrapid 100units/ml solution for injection 10ml vials (Novo Nordisk Ltd)
	06010101		Pork Actrapid Vial 10ml INJ 100UNITS/ML
	06010102		Pork Insulatard Injection 100 units/ml 10 ml vial
	06.01.01.02.00		Pork Insulatard 100units/ml suspension for injection 10ml vials (Novo Nordisk Ltd)
	06010102		Pork Insulatard Vial 10ml INJ 100UNITS/ML
	06.01.01.02.00		Pork Mixtard 30 100units/ml suspension for injection 10ml vials (Novo Nordisk Ltd)
	0601012D0BCABAI		Pork Mixtard 30 Vial 10ml INJ 100UNITS/ML
	0601011N0BFAAAC		Pork Velosulin Injection 100 units/ml
	06.01.01.01.00		PORK VELOSULIN injection 100 units/ml [NOVO]
	0601011N0BFAAAC		PORK VELOSULIN VIAL 10ML INJ 100UNITS/ML
	06.01.02.01.00		RASTINON tablets 500mg [HOECHSTMAR]
	06.01.02.03.00		Repaglinide 1mg tablets
	06.01.02.03.00		Repaglinide 1mg tablets (Actavis UK Ltd)
	06.01.02.03.00		Repaglinide 2mg tablets
	06.01.02.03.00		Repaglinide 500microgram tablets
	0601023R0AAABAB		Repaglinide TABS 1MG
	06.01.02.03.00		ROMOZIN tablets 200mg [GLAXO]
	06.01.02.02.00		Rosiglitazone 1mg / Metformin 500mg tablets
	06.01.02.03.00		Rosiglitazone 2mg / Metformin 1g tablets
	06.01.02.03.00		Rosiglitazone 2mg / Metformin 500mg tablets
	06.01.02.03.00		Rosiglitazone 4mg / Metformin 1g tablets
	06.01.02.03.00		Rosiglitazone 4mg tablets
	06010203		ROSIGLITAZONE 4MG TABLETS
	06010203		Rosiglitazone 8mg tab
	06.01.02.03.00		Rosiglitazone 8mg tablets
	06010203		Rosiglitazone And Metformin Tablets 2 mg + 1 gram
	06010203		Rosiglitazone And Metformin Tablets 2 mg + 500 mg
	06010203		Rosiglitazone And Metformin Tablets 4 mg + 1 gram
	06010203		Rosiglitazone Maleate Tablets 4 mg
	06010203		Rosiglitazone Maleate Tablets 8 mg
	06010203		ROSIGLITAZONE TABLETS 4MG
	06010203		ROSIGLITAZONE TABLETS 8MG
	06010203		Rosiglitazone TABS 4MG
	06010203		Rosiglitazone TABS 8MG
	06010203		Saxagliptin Tablets 2.5 mg
	06010203		Saxagliptin Tablets 5 mg
	06.01.02.03.00		Saxagliptin 2.5mg / Metformin 1g tablets
ftdy.	06010203		Saxagliptin 2.5mg tablets
	06.01.02.03.00		Saxagliptin 2.5mg tablets

ftdz.	06010203		Saxagliptin 5mg tablets
	06.01.02.03.00		Saxagliptin 5mg tablets
	06010203		SAXAGLIPTIN TABLETS 5MG
	06010203		Sitagliptin Tablets 100 mg
	06010203		Sitagliptin Tablets 25 mg
	06010203		Sitagliptin Tablets 50 mg
ft8z.	06010203		Sitagliptin 100mg tablets
	06.01.02.03.00		Sitagliptin 100mg tablets
ft8x.	06010203		Sitagliptin 25mg tablets
	06.01.02.03.00		Sitagliptin 25mg tablets
ft8y.	06010203		Sitagliptin 50mg tablets
	06.01.02.03.00		Sitagliptin 50mg tablets
	06010203		SITAGLIPTIN TABLETS 100 MG
	06010203		Sitagliptin TABLETS 100MG
	06010203		SITAGLIPTIN TABLETS 100MG
ft8z.	06010203		SITAGLIPTIN tabs 100mg
	06010203		Sitagliptin TABS 100MG
	06.01.02.02.00		Sukkarto SR 1000mg tablets (Morningside Healthcare Ltd)
	06.01.02.02.00		Sukkarto SR 500mg tablets (Morningside Healthcare Ltd)
	06.01.02.01.00		TOLANASE tablets 250mg [PHARMACIA]
	06.01.02.01.00		tolazamide tablets 100mg
	06.01.02.01.00		tolazamide tablets 250mg
	0601021X0AAADAD		Tolbutamide Tablets 500 mg
f3a1.	06010201		Tolbutamide 500mg tablets
	06.01.02.01.00		Tolbutamide 500mg tablets
	06.01.02.01.00		Tolbutamide 500mg tablets (Actavis UK Ltd)
	0601021X0AAADAD		Tolbutamide TABS 500MG
f299.	06010102		Toujeo 300units/ml solution for injection 1.5ml pre-fille...
	06.01.01.02.00		Toujeo 300units/ml solution for injection 1.5ml pre-filled SoloStar pen (Sanofi)
	06010102		Toujeo Solostar Pre-filled Disposable Pen 300 units/ml 1.5 ml pen
	06.01.02.03.00		Trajenta 5mg tablets (Boehringer Ingelheim Ltd)
	06010102		Tresiba Flextouch Solution for injection 100 units/ml 3 ml pre-filled pen
f2B1.	06010102		Tresiba FlexTouch 100units/ml solution for injection 3ml ...
	06.01.01.02.00		Tresiba FlexTouch 100units/ml solution for injection 3ml pre-filled pen (Novo Nordisk Ltd)
	06.01.01.02.00		Tresiba FlexTouch 200units/ml solution for injection 3ml pre-filled pen (Novo Nordisk Ltd)
	06.01.01.02.00		Tresiba Penfill 100units/ml solution for injection 3ml cartridges (Novo Nordisk Ltd)

	06.01.02.03.00		Trulicity 0.75mg/0.5ml solution for injection pre-filled pen (Eli Lilly and Company Ltd)
ftq7.	06010203		Trulicity 1.5mg/0.5ml solution for injection pre-filled p...
	06.01.02.03.00		Trulicity 1.5mg/0.5ml solution for injection pre-filled pen (Eli Lilly and Company Ltd)
	06.01.01.04.00		u100 insulin syringe 0.5ml
	06.01.01.04.00		u100 single use insulin syringe with 12mm needle(26G) 0.5ml
	06.01.01.03.00		u100 single use insulin syringe with 12mm needle(26G) 1ml
	06.01.01.04.00		u100 single use insulin syringe with 12mm needle(27G) 0.3ml
	06.01.01.03.00		u100 single use insulin syringe with 12mm needle(28G) 0.3ml
	06.01.01.02.00		Ultratard 100units/ml suspension for injection 10ml vials (Novo Nordisk Ltd)
	06.01.02.01.00		Vamju 30mg modified-release tablets (AMCo)
	06.01.03.00.00		Velosulin 100units/ml solution for injection 10ml vials (Novo Nordisk Ltd)
	06.01.01.01.00		VELOSULIN CARTRIDGE injection 100 units/ml [NOVO]
	06010203		Victoza Solution for injection in pre-filled pen 6 mg/ml 3 ml pen
	06.01.02.03.00		Victoza 6mg/ml solution for injection 3ml pre-filled pen (Novo Nordisk Ltd)
ftc1.	06010203		Victoza 6mg/ml solution for injection 3ml pre-filled pen ...
	06010203		Victoza Prefilled Pen 3ml INJ 6MG/ML
	06010203		VICTOZA PREFILLED PEN 3ML INJ 6MG/ML
	06010203		Vildagliptin Tablets 50 mg
	06.01.02.03.00		Vildagliptin 50mg / Metformin 1g tablets
	06.01.02.02.00		Vildagliptin 50mg / Metformin 850mg tablets
ftaZ.	06010203		Vildagliptin 50mg tablets
	06.01.02.03.00		Vildagliptin 50mg tablets
	06010203		VILDAGLIPTIN TABLETS 50MG
	06010203		VILDAGLIPTIN/METFORMIN TABLETS 50MG/1000MG
	06.01.02.03.00		Vipdomet 12.5mg/1000mg tablets (Takeda UK Ltd)
	06.01.02.03.00		Vipidia 25mg tablets (Takeda UK Ltd)
	06.01.01.03.00		Vitrex Soft lancets 0.65mm/23gauge (Vitrex Medical Ltd)
	06010203		Xultophy Solution for injection 100 units/ml + 3.6 mg/ml 3 ml pen
f2C1.	06010203		Xultophy 100units/ml / 3.6mg/ml solution for injection 3m...
	06.01.02.03.00		Xultophy 100units/ml / 3.6mg/ml solution for injection 3ml pre-filled pen (Novo Nordisk Ltd)
	06010201		Zicron Tablets 40 mg
	06.01.02.01.00		Zicron 40mg tablets (Bristol Laboratories Ltd)
**Thyroid drugs**			

	06.02.01.00.00		levothyroxine capsules
	06.02.01.00.00		levothyroxine lactose free oral suspension sugar-free 100microgram/5ml
	06.02.01.00.00		levothyroxine lactose free oral suspension sugar-free 25micrograms/ml
	06.02.01.00.00		levothyroxine oral liquid
	06.02.01.00.00		levothyroxine oral powder
	06.02.01.00.00		levothyroxine oral suspension 25micrograms/5ml
	06.02.01.00.00		levothyroxine oral suspension 50micrograms/5ml
f929.00		1.075E+16	LEVOTHYROXINE sf oral soln 100microgram/5ml
f927.00		1.075E+16	LEVOTHYROXINE sf oral soln 25micrograms/5ml
f928.00		1.075E+16	LEVOTHYROXINE sf oral soln 50micrograms/5ml
	60201		Levothyroxine Sodium Oral solution Sugar Free 100 micrograms/5 ml
	60201		Levothyroxine Sodium Suspension 100 micrograms/5 ml
f923.00		374296008	Levothyroxine Sodium Tablets 100 micrograms
	60201		Levothyroxine Sodium Tablets 100 micrograms
f923.00		374296008	Levothyroxine Sodium Tablets 100 micrograms~(f923.)
f921.00		374294006	Levothyroxine Sodium Tablets 25 micrograms
	60201		Levothyroxine Sodium Tablets 25 micrograms
f922.00		374295007	Levothyroxine Sodium Tablets 50 micrograms
	60201		Levothyroxine Sodium Tablets 50 micrograms
f92..00		424608008	Levothyroxine sodium 100microgram capsules
	06.02.01.00.00		Levothyroxine sodium 100microgram capsules
f92..00		8.8164E+15	Levothyroxine sodium 100microgram oral powder sachets
f923.	6020100		Levothyroxine sodium 100microgram tablets
f923.00		374296008	Levothyroxine sodium 100microgram tablets
	06.02.01.00.00		Levothyroxine sodium 100microgram tablets
	06.02.01.00.00		Levothyroxine sodium 100microgram tablets (A A H Pharmaceuticals Ltd)
	06.02.01.00.00		Levothyroxine sodium 100microgram tablets (Actavis UK Ltd)
	06.02.01.00.00		Levothyroxine sodium 100microgram tablets (Almus Pharmaceuticals Ltd)
	06.02.01.00.00		Levothyroxine sodium 100microgram tablets (IVAX Pharmaceuticals UK Ltd)
		2.1031E+14	Levothyroxine sodium 100microgram tablets (IVAX Pharmaceuticals UK Ltd)
	06.02.01.00.00		Levothyroxine sodium 100microgram tablets (Mylan Ltd)
	06.02.01.00.00		Levothyroxine sodium 100microgram tablets (Teva UK Ltd)
		9.0201E+14	Levothyroxine sodium 100microgram tablets (Teva UK Ltd)
f929.	6020100		Levothyroxine sodium 100micrograms/5ml oral solution suga...

f929.00		1.075E+16	Levothyroxine sodium 100micrograms/5ml oral solution sugar free
	06.02.01.00.00		Levothyroxine sodium 100micrograms/5ml oral solution sugar free
	06.02.01.00.00		Levothyroxine sodium 100micrograms/5ml oral solution sugar free (A A H Pharmaceuticals Ltd)
	06.02.01.00.00		Levothyroxine sodium 100micrograms/5ml oral solution sugar free (Teva UK Ltd)
f92..	6020100		Levothyroxine sodium 12.5microgram tablets
f92..00		3.362E+16	Levothyroxine sodium 12.5microgram tablets
	06.02.01.00.00		Levothyroxine sodium 250micrograms/5ml oral solution
	06.02.01.00.00		Levothyroxine sodium 250micrograms/5ml oral suspension
f92..	6020100		Levothyroxine sodium 25microgram capsules
f92..00		424458007	Levothyroxine sodium 25microgram capsules
	06.02.01.00.00		Levothyroxine sodium 25microgram capsules
f921.	6020100		Levothyroxine sodium 25microgram tablets
f921.00		374294006	Levothyroxine sodium 25microgram tablets
	06.02.01.00.00		Levothyroxine sodium 25microgram tablets
	06.02.01.00.00		Levothyroxine sodium 25microgram tablets (A A H Pharmaceuticals Ltd)
	06.02.01.00.00		Levothyroxine sodium 25microgram tablets (Actavis UK Ltd)
		4.8011E+13	Levothyroxine sodium 25microgram tablets (Actavis UK Ltd)
	06.02.01.00.00		Levothyroxine sodium 25microgram tablets (AMCo)
	06.02.01.00.00		Levothyroxine sodium 25microgram tablets (IVAX Pharmaceuticals UK Ltd)
	06.02.01.00.00		Levothyroxine sodium 25microgram tablets (Teva UK Ltd)
	06.02.01.00.00		Levothyroxine sodium 25microgram tablets (Wockhardt UK Ltd)
f927.00		1.075E+16	Levothyroxine sodium 25micrograms/5ml oral solution sugar free
	06.02.01.00.00		Levothyroxine sodium 25micrograms/5ml oral solution sugar free
f927.00		1.075E+16	Levothyroxine sodium 25micrograms/5ml oral solution sugar...
	06.02.01.00.00		Levothyroxine sodium 38microgram / Liothyronine 9microgram tablets
f92..00		424293002	Levothyroxine sodium 50microgram capsules
	06.02.01.00.00		Levothyroxine sodium 50microgram capsules
f922.	6020100		Levothyroxine sodium 50microgram tablets
f922.00		374295007	Levothyroxine sodium 50microgram tablets
	60201		Levothyroxine sodium 50microgram tablets
	06.02.01.00.00		Levothyroxine sodium 50microgram tablets
	06.02.01.00.00		Levothyroxine sodium 50microgram tablets (A A H Pharmaceuticals Ltd)

	06.02.01.00.00		Levothyroxine sodium 50microgram tablets (Actavis UK Ltd)
f922.00		9.8095E+15	Levothyroxine sodium 50microgram tablets (Almus Pharmaceu...
	06.02.01.00.00		Levothyroxine sodium 50microgram tablets (Almus Pharmaceuticals Ltd)
	06.02.01.00.00		Levothyroxine sodium 50microgram tablets (Mylan Ltd)
	06.02.01.00.00		Levothyroxine sodium 50microgram tablets (Sigma Pharmaceuticals Plc)
f922.00		8.3011E+13	Levothyroxine sodium 50microgram tablets (Teva UK Ltd)
	06.02.01.00.00		Levothyroxine sodium 50microgram tablets (Teva UK Ltd)
		8.3011E+13	Levothyroxine sodium 50microgram tablets (Teva UK Ltd)
f928.00		1.075E+16	Levothyroxine sodium 50micrograms/5ml oral solution sugar free
	06.02.01.00.00		Levothyroxine sodium 50micrograms/5ml oral solution sugar free
f92..	6020100		Levothyroxine sodium 75microgram tablets
f92..00		376988009	Levothyroxine sodium 75microgram tablets
	60201		LEVOTHYROXINE SODIUM NON TARIFF TABLETS 100MICROGRAMS
	60201		Levothyroxine Sodium Non Tariff TABS 25MICROGRAMS
	60201		Levothyroxine Sodium Sf Oral SOLN 25MCG/5ML
f923.00		374296008	Levothyroxine Sodium Tablets 100 micrograms
f923.00		374296008	Levothyroxine Sodium Tablets 100micrograms
	60201		Levothyroxine Sodium TABLETS 100MICROGRAMS
	60201		LEVOTHYROXINE SODIUM TABLETS 100MICROGRAMS
f921.00		374294006	Levothyroxine Sodium Tablets 25 micrograms
	60201		Levothyroxine Sodium TABLETS 25MICROGRAMS
	60201		LEVOTHYROXINE SODIUM TABLETS 25MICROGRAMS
f922.00		374295007	Levothyroxine Sodium Tablets 50 micrograms
	60201		Levothyroxine Sodium TABLETS 50MICROGRAMS
	60201		LEVOTHYROXINE SODIUM TABLETS 50MICROGRAMS
	60201		Levothyroxine Sodium TABS 100MICROGRAMS
	60201		Levothyroxine Sodium TABS 25MICROGRAMS
	60201		Levothyroxine Sodium TABS 50MICROGRAMS
f929.00		1.075E+16	levothyroxine sugar free oral solution 100microgram/5ml
f927.00		1.075E+16	levothyroxine sugar free oral solution 25micrograms/5ml
f923.00		374296008	levothyroxine tablets 100micrograms
f923.00		374296008	Levothyroxine tablets 100micrograms
f921.00		374294006	levothyroxine tablets 25micrograms
f921.00		374294006	Levothyroxine tablets 25micrograms
f921.00		374294006	LEVOTHYROXINE tablets 25micrograms
	0602010V0AABWBW		LEVOTHYROXINE TABLETS 25MICROGRAMS
f922.00		374295007	levothyroxine tablets 50micrograms

f922.00		374295007	Levothyroxine tablets 50micrograms
f922.00		374295007	LEVOTHYROXINE tablets 50micrograms
	60201		LEVOTHYROXINE TABLETS 50MICROGRAMS
		2.9928E+17	LEVOTHYROXINE tablets 50micrograms
f923.	6020100		LEVOTHYROXINE tabs 100micrograms
f923.00		374296008	LEVOTHYROXINE tabs 100micrograms
		9.0201E+14	LEVOTHYROXINE tabs 100micrograms [APS]
		2.1031E+14	LEVOTHYROXINE tabs 100micrograms [IVAX]
		9.0201E+14	LEVOTHYROXINE tabs 100micrograms [TEVA]
f921.	6020100		LEVOTHYROXINE tabs 25micrograms
f921.00		374294006	LEVOTHYROXINE tabs 25micrograms
		4.8011E+13	LEVOTHYROXINE tabs 25micrograms [ACTAVIS]
		4.8011E+13	LEVOTHYROXINE tabs 25micrograms [ALPHAR/COX]
f922.	6020100		LEVOTHYROXINE tabs 50micrograms
f922.00		374295007	LEVOTHYROXINE tabs 50micrograms
		8.3011E+13	LEVOTHYROXINE tabs 50micrograms [APS]
		8.3011E+13	LEVOTHYROXINE tabs 50micrograms [TEVA]
**Rhythm and rate-control**			
Read2	0203020D0AAAAAA		AMIODARONE
	0203020D0AAABAB		AMIODARONE
	2030201		AMIODARONE
		Amiodarone 100mg tablets	AMIODARONE
		Amiodarone 100mg tablets (Actavis UK Ltd)	AMIODARONE
		Amiodarone 100mg tablets (Teva UK Ltd)	AMIODARONE
		Amiodarone 200mg tablets	AMIODARONE
		Amiodarone 200mg tablets (A A H Pharmaceuticals Ltd)	AMIODARONE
		Amiodarone 200mg tablets (Actavis UK Ltd)	AMIODARONE
		Amiodarone 200mg tablets (Teva UK Ltd)	AMIODARONE
		Cordarone X 200 tablets (Sanofi)	AMIODARONE
	02.08.02.00.00		APIXABAN
	20802		APIXABAN
	2080200		APIXABAN
	02.02.01.00.00		BISOPROLOL
	0204000H0AAAAAA		BISOPROLOL

	0204000H0AAABAB		BISOPROLOL
	0204000H0BCABAB		BISOPROLOL
	204		BISOPROLOL
	2040000		BISOPROLOL
		Bisoprolol 5mg tablets	BISOPROLOL
		Bisoprolol Fumarate Tablets 5mg	BISOPROLOL
		BISOPROLOL tabs 5mg	BISOPROLOL
		Bisoprolol 10mg tablets	BISOPROLOL
		BISOPROLOL tabs 10mg	BISOPROLOL
		Cardicor 2.5mg tablets (Merck Serono Ltd)	BISOPROLOL
		Bisoprolol 1.25mg tablets	BISOPROLOL
		BISOPROLOL tabs 1.25mg	BISOPROLOL
		Bisoprolol 2.5mg tablets	BISOPROLOL
		BISOPROLOL tabs 2.5mg	BISOPROLOL
		Bisoprolol 3.75mg tablets	BISOPROLOL
		Bisoprolol 7.5mg tablets	BISOPROLOL
		Bisoprolol 10mg tablets	BISOPROLOL
		Bisoprolol 10mg tablets (A A H Pharmaceuticals Ltd)	BISOPROLOL
		Bisoprolol 10mg tablets (Actavis UK Ltd)	BISOPROLOL
		Bisoprolol 10mg tablets (Almus Pharmaceuticals Ltd)	BISOPROLOL
		Bisoprolol 10mg tablets (Mylan Ltd)	BISOPROLOL
		Bisoprolol 10mg tablets (Niche Generics Ltd)	BISOPROLOL
		Bisoprolol 10mg tablets (Teva UK Ltd)	BISOPROLOL
		Bisoprolol 1.25mg tablets	BISOPROLOL
		Bisoprolol 1.25mg tablets (A A H Pharmaceuticals Ltd)	BISOPROLOL

		Bisoprolol 1.25mg tablets (Almus Pharmaceuticals Ltd)	BISOPROLOL
		Bisoprolol 1.25mg tablets (Mylan Ltd)	BISOPROLOL
		Bisoprolol 1.25mg tablets (Teva UK Ltd)	BISOPROLOL
		Bisoprolol 2.5mg/5ml oral suspension	BISOPROLOL
		Bisoprolol 2.5mg tablets	BISOPROLOL
		Bisoprolol 2.5mg tablets (A A H Pharmaceuticals Ltd)	BISOPROLOL
		Bisoprolol 2.5mg tablets (Actavis UK Ltd)	BISOPROLOL
		Bisoprolol 2.5mg tablets (Almus Pharmaceuticals Ltd)	BISOPROLOL
		Bisoprolol 2.5mg tablets (Mylan Ltd)	BISOPROLOL
		Bisoprolol 2.5mg tablets (Teva UK Ltd)	BISOPROLOL
		Bisoprolol 3.75mg tablets	BISOPROLOL
		Bisoprolol 3.75mg tablets (A A H Pharmaceuticals Ltd)	BISOPROLOL
		Bisoprolol 3.75mg tablets (Almus Pharmaceuticals Ltd)	BISOPROLOL
		Bisoprolol 3.75mg tablets (Teva UK Ltd)	BISOPROLOL
		Bisoprolol 5mg/5ml oral suspension	BISOPROLOL
		Bisoprolol 5mg tablets	BISOPROLOL
		Bisoprolol 5mg tablets (A A H Pharmaceuticals Ltd)	BISOPROLOL
		Bisoprolol 5mg tablets (Actavis UK Ltd)	BISOPROLOL
		Bisoprolol 5mg tablets (C P Pharmaceuticals Ltd)	BISOPROLOL
		Bisoprolol 5mg tablets (Kent Pharmaceuticals Ltd)	BISOPROLOL
		Bisoprolol 5mg tablets (Ranbaxy (UK) Ltd)	BISOPROLOL

		Bisoprolol 5mg tablets (Sandoz Ltd)	BISOPROLOL
		Bisoprolol 5mg tablets (Teva UK Ltd)	BISOPROLOL
		Bisoprolol 7.5mg tablets	BISOPROLOL
		Bisoprolol 7.5mg tablets (A A H Pharmaceuticals Ltd)	BISOPROLOL
		Bisoprolol 7.5mg tablets (Almus Pharmaceuticals Ltd)	BISOPROLOL
		Cardicor 10mg tablets (Merck Serono Ltd)	BISOPROLOL
		Cardicor 1.25mg tablets (Merck Serono Ltd)	BISOPROLOL
		Cardicor 2.5mg tablets (Merck Serono Ltd)	BISOPROLOL
		Cardicor 3.75mg tablets (Merck Serono Ltd)	BISOPROLOL
		Cardicor 5mg tablets (Merck Serono Ltd)	BISOPROLOL
		Cardicor 7.5mg tablets (Merck Serono Ltd)	BISOPROLOL
		Monocor 10mg tablets (Wyeth Pharmaceuticals)	BISOPROLOL
		Monocor 5mg tablets (Wyeth Pharmaceuticals)	BISOPROLOL
	020400080AAABAB		CARVEDILOL
	020400080AAACAC		CARVEDILOL
	020400080AAAEAE		CARVEDILOL
	020400080AAAFAF		CARVEDILOL
	2040000		CARVEDILOL
		Carvedilol 12.5mg tablets	CARVEDILOL
		Carvedilol 25mg tablets	CARVEDILOL
		Carvedilol 3.125mg tablets	CARVEDILOL
		Carvedilol 6.25mg tablets	CARVEDILOL
		Carvedilol 12.5mg tablets	CARVEDILOL

		Carvedilol 12.5mg tablets (Almus Pharmaceuticals Ltd)	CARVEDILOL
		Carvedilol 12.5mg tablets (Teva UK Ltd)	CARVEDILOL
		Carvedilol 25mg tablets	CARVEDILOL
		Carvedilol 3.125mg tablets	CARVEDILOL
		Carvedilol 3.125mg tablets (A A H Pharmaceuticals Ltd)	CARVEDILOL
		Carvedilol 6.25mg tablets	CARVEDILOL
		Eucardic 12.5mg tablets (Roche Products Ltd)	CARVEDILOL
		Eucardic 25mg tablets (Roche Products Ltd)	CARVEDILOL
	02.08.02.00.00		DABIGATRAN
	20802		DABIGATRAN
	2080200		DABIGATRAN
	02.01.01.00.00		DIGITOXIN
	02.01.01.00.00		DIGOXIN
	0201010F0AAAAAA		DIGOXIN
	0201010F0AAADAD		DIGOXIN
	0201010F0AAAEAE		DIGOXIN
	0201010F0AAAFAF		DIGOXIN
	2010100		DIGOXIN
	02.06.02.00.00		DILTIAZEM
	0206020C0AAAAAA		DILTIAZEM
	0206020C0AAACAC		DILTIAZEM
	0206020C0AAAEAE		DILTIAZEM
	0206020C0AAAJAJ		DILTIAZEM
	0206020C0AAASAS		DILTIAZEM
	0206020C0AAATAT		DILTIAZEM
	0206020C0AAAUAU		DILTIAZEM
	0206020C0AAAVAV		DILTIAZEM
	0206020C0AAAXAX		DILTIAZEM
	0206020C0AAAYAY		DILTIAZEM
	2060200		DILTIAZEM
	20302		DRONEDARONE
	2030202		DRONEDARONE
		Dronedarone 400mg tablets	DRONEDARONE

		DRONEDARONE tabs 400mg	DRONEDARONE
	02.08.02.00.00		EDOXABAN
	0203020I0AAADAD		FLECAINIDE
	0203020I0AAAKAK		FLECAINIDE
	2030200		FLECAINIDE
	2030201		FLECAINIDE
		Flecainide 100mg tablets	FLECAINIDE
		Flecainide 100mg tablets (A A H Pharmaceuticals Ltd)	FLECAINIDE
		Flecainide 100mg tablets (Almus Pharmaceuticals Ltd)	FLECAINIDE
		Flecainide 100mg tablets (Mylan Ltd)	FLECAINIDE
		Flecainide 100mg tablets (Zentiva)	FLECAINIDE
		Flecainide 200mg modified-release capsules	FLECAINIDE
		Flecainide 25mg/5ml oral suspension	FLECAINIDE
		Flecainide 50mg tablets	FLECAINIDE
		Flecainide 50mg tablets (A A H Pharmaceuticals Ltd)	FLECAINIDE
		Flecainide 50mg tablets (Actavis UK Ltd)	FLECAINIDE
		Flecainide 50mg tablets (Almus Pharmaceuticals Ltd)	FLECAINIDE
		Flecainide 50mg tablets (Mylan Ltd)	FLECAINIDE
		Flecainide 50mg tablets (Teva UK Ltd)	FLECAINIDE
		Tambocor 100mg tablets (Meda Pharmaceuticals Ltd)	FLECAINIDE
		Tambocor 50mg tablets (Meda Pharmaceuticals Ltd)	FLECAINIDE
		Tambocor XL 200mg capsules (Meda Pharmaceuticals Ltd)	FLECAINIDE
	0204000K0AAABAB		METOPROLOL
	0204000K0AAACAC		METOPROLOL

	0204000K0AAADAD		METOPROLOL
	2040000		METOPROLOL
		Betaloc 100mg tablets (AstraZeneca UK Ltd)	METOPROLOL
		Betaloc 50mg tablets (AstraZeneca UK Ltd)	METOPROLOL
		Betaloc-SA 200mg tablets (AstraZeneca UK Ltd)	METOPROLOL
		Lopresor SR 200mg tablets (Recordati Pharmaceuticals Ltd)	METOPROLOL
		LOPRESOR tablets 100mg [NOVARTIS]	METOPROLOL
		LOPRESOR tablets 50mg [NOVARTIS]	METOPROLOL
		Metoprolol 100mg / Hydrochlorothiazide 12.5mg tablets	METOPROLOL
		Metoprolol 100mg tablets	METOPROLOL
		Metoprolol 100mg tablets (Mylan Ltd)	METOPROLOL
		Metoprolol 100mg tablets (Sandoz Ltd)	METOPROLOL
		Metoprolol 200mg modified-release tablets	METOPROLOL
		Metoprolol 50mg tablets	METOPROLOL
		Metoprolol 50mg tablets (A A H Pharmaceuticals Ltd)	METOPROLOL
		Metoprolol 50mg tablets (Actavis UK Ltd)	METOPROLOL
		Metoprolol 50mg tablets (Almus Pharmaceuticals Ltd)	METOPROLOL
		Metoprolol 50mg tablets (Mylan Ltd)	METOPROLOL
		Metoprolol 50mg tablets (Sandoz Ltd)	METOPROLOL
		Metoprolol 50mg tablets (Teva UK Ltd)	METOPROLOL
		Metoprolol 5mg/5ml solution for injection ampoules	METOPROLOL
	204		NEBIVOLOL
	2040000		NEBIVOLOL

		Nebivolol 2.5mg tablets	NEBIVOLOL
		Nebivolol 5mg tablets	NEBIVOLOL
		Nebilet 5mg tablets (A. Menarini Farmaceutica Internazionale SRL)	NEBIVOLOL
		Nebivolol 10mg tablets	NEBIVOLOL
		Nebivolol 2.5mg tablets	NEBIVOLOL
		Nebivolol 2.5mg tablets (A A H Pharmaceuticals Ltd)	NEBIVOLOL
		Nebivolol 5mg tablets	NEBIVOLOL
		Nebivolol 5mg tablets (Actavis UK Ltd)	NEBIVOLOL
		Nebivolol 5mg tablets (Teva UK Ltd)	NEBIVOLOL
		Nebivolol 5mg tablets (Zentiva)	NEBIVOLOL
	0203020R0AAAAAA		PROPAFENONE
	0203020R0AAABAB		PROPAFENONE
	2030201		PROPAFENONE
		Arythmol 150mg tablets (BGP Products Ltd)	PROPAFENONE
		Propafenone 150mg tablets	PROPAFENONE
		Propafenone 300mg tablets	PROPAFENONE
	02.08.02.00.00		RIVAROXABAN
	20802		RIVAROXABAN
	2080200		RIVAROXABAN
	0204000T0AAABAB		SOTALOL
	0204000T0AAACAC		SOTALOL
	0204000T0AAAEAE		SOTALOL
	2040000		SOTALOL
		Sotalol 40mg tablets	SOTALOL
		Sotalol 80mg tablets	SOTALOL
		Sotacor 160mg tablets (Bristol-Myers Squibb Pharmaceuticals Ltd)	SOTALOL
		Sotacor 80mg tablets (Bristol-Myers Squibb Pharmaceuticals Ltd)	SOTALOL
		Sotalol 40mg tablets	SOTALOL

		Sotalol 40mg tablets (A A H Pharmaceuticals Ltd)	SOTALOL
		Sotalol 40mg tablets (Teva UK Ltd)	SOTALOL
		Sotalol 40mg tablets (Tillomed Laboratories Ltd)	SOTALOL
		Sotalol 80mg tablets (Sandoz Ltd)	SOTALOL
		SOTAZIDE tablets [BRISTOL]	SOTALOL
		Beta-Cardone 200mg tablets (Focus Pharmaceuticals Ltd)	SOTALOL
		Beta-Cardone 40mg tablets (Focus Pharmaceuticals Ltd)	SOTALOL
		Beta-Cardone 80mg tablets (Focus Pharmaceuticals Ltd)	SOTALOL
		Sotalol 160mg tablets	SOTALOL
		Sotalol 200mg tablets	SOTALOL
		Sotalol 80mg tablets	SOTALOL
		Sotalol 80mg tablets (A A H Pharmaceuticals Ltd)	SOTALOL
		Sotalol 80mg tablets (Teva UK Ltd)	SOTALOL
		Sotalol 40mg tablets	SOTALOL
		Sotalol 80mg tablets	SOTALOL
		Sotacor 160mg tablets (Bristol-Myers Squibb Pharmaceuticals Ltd)	SOTALOL
		Sotacor 80mg tablets (Bristol-Myers Squibb Pharmaceuticals Ltd)	SOTALOL
		Sotalol 40mg tablets	SOTALOL
		Sotalol 40mg tablets (A A H Pharmaceuticals Ltd)	SOTALOL
		Sotalol 40mg tablets (Teva UK Ltd)	SOTALOL
		Sotalol 40mg tablets (Tillomed Laboratories Ltd)	SOTALOL
		Sotalol 80mg tablets (Sandoz Ltd)	SOTALOL

		SOTAZIDE tablets [BRISTOL]	SOTALOL
		Beta-Cardone 200mg tablets (Focus Pharmaceuticals Ltd)	SOTALOL
		Beta-Cardone 40mg tablets (Focus Pharmaceuticals Ltd)	SOTALOL
		Beta-Cardone 80mg tablets (Focus Pharmaceuticals Ltd)	SOTALOL
		Sotalol 160mg tablets	SOTALOL
		Sotalol 200mg tablets	SOTALOL
		Sotalol 80mg tablets	SOTALOL
		Sotalol 80mg tablets (A A H Pharmaceuticals Ltd)	SOTALOL
		Sotalol 80mg tablets (Teva UK Ltd)	SOTALOL
	02.06.02.00.00		VERAPAMIL
	0206020T0AAACAC		VERAPAMIL
	0206020T0AAADAD		VERAPAMIL
	0206020T0AAAFAF		VERAPAMIL
	0206020T0AAAGAG		VERAPAMIL
	0206020T0AAAHAH		VERAPAMIL
	0206020T0AAAIAI		VERAPAMIL
	0206020T0AAAJAJ		VERAPAMIL
	0206020T0AAAKAK		VERAPAMIL
	0206020T0AAAUAU		VERAPAMIL
	2030202		VERAPAMIL
	2060200		VERAPAMIL
		Verapamil 40mg tablets	VERAPAMIL
		Verapamil 80mg tablets	VERAPAMIL
		Verapamil 120mg tablets	VERAPAMIL
		Verapamil 240mg modified-release tablets	VERAPAMIL
		Half Securon SR 120mg tablets (Abbott Laboratories Ltd)	VERAPAMIL
		Half Securon SR 120mg tablets (BGP Products Ltd)	VERAPAMIL

		Securon SR 240mg tablets (Abbott Laboratories Ltd)	VERAPAMIL
		Securon SR 240mg tablets (BGP Products Ltd)	VERAPAMIL
		SECURON SR tabs 240mg	VERAPAMIL
		Univer 180mg modified-release capsules (Teva UK Ltd)	VERAPAMIL
		VERAPAMIL HCl tabs 160mg	VERAPAMIL
		Verapamil 240mg modified-release capsules	VERAPAMIL
		Cordilox 120mg tablets (IVAX Pharmaceuticals UK Ltd)	VERAPAMIL
		Securon 120mg tablets (Abbott Laboratories Ltd)	VERAPAMIL
		Securon SR 240mg tablets (BGP Products Ltd)	VERAPAMIL
		SECURON tablets 80mg [ABBOTT]	VERAPAMIL
		Univer 120mg modified-release capsules (Teva UK Ltd)	VERAPAMIL
		Univer 180mg modified-release capsules (Teva UK Ltd)	VERAPAMIL
		Univer 240mg modified-release capsules (Teva UK Ltd)	VERAPAMIL
		Verapamil 120mg modified-release tablets (A A H Pharmaceuticals Ltd)	VERAPAMIL
		Verapamil 120mg tablets	VERAPAMIL
		Verapamil 120mg tablets (A A H Pharmaceuticals Ltd)	VERAPAMIL
		Verapamil 120mg tablets (Actavis UK Ltd)	VERAPAMIL
		Verapamil 160mg tablets (A A H Pharmaceuticals Ltd)	VERAPAMIL

		Verapamil 240mg modified-release capsules	VERAPAMIL
		Verapamil 240mg modified-release tablets	VERAPAMIL
		Verapamil 240mg modified-release tablets (Teva UK Ltd)	VERAPAMIL
		Verapamil 40mg/5ml oral solution sugar free	VERAPAMIL
		Verapamil 40mg tablets	VERAPAMIL
		Verapamil 40mg tablets (Actavis UK Ltd)	VERAPAMIL
		Verapamil 80mg tablets	VERAPAMIL
		Verapamil 80mg tablets (Kent Pharmaceuticals Ltd)	VERAPAMIL
		Verapress MR 240mg tablets (Actavis UK Ltd)	VERAPAMIL
		Vera-Til SR 120mg tablets (Tillomed Laboratories Ltd)	VERAPAMIL

Supplemental table for drug coding.

	Condition	Fields	ICD-10s	OPCS-4	Drugs relation	Comments
**Inclusion**						
	Recent-onset AF (≤ 1 year prior to enrolment for prevalent - or after recruitment for incident)	53, 131350				
**2 One of:**						
	Age > 75 years	21022, 53, 131350				
	Prior stroke or transient ischemic attack	HES	G45, I60-I64			
**3 OR two of the following:**						
	Age > 65 years	21022, 53, 131350				
	Female sex	31				
	Arterial hypertension (chronic treatment for hypertension, estimated need for continuous antihypertensive threapy or resting blood pressure > 145/90 mmHg)	4079, 94, 4080, 93, 6177, 6153, HES	I10-I15			Males (6177) coding 2; Female (6153) coding 2
	Diabetes mellitus (treated by drugs or insulin) or impaired glucose tolerance	30750, HES, GP	E10-E14		yes	
	Severe coronary artery disease (previous	HES, HES OPS	I21-I25	K40-K51		

	myocardial infarction, CABG or PCI)					
	Stable heart failure (NYHA II or LVEF <50%), OR heart failure medication (ACE inhibitors, Angiotensin receptor blockers, Sacubitril/valsartan, Spironolactone, Eplerenone, SGLTi	HES, GP	I50, I110, I130, I132		yes	
	Chronic kidney disease (MDRD stage III or IV)	21000, 30720, 30700, 21022, 31, HES	N183, N184			Fields needed for GFR calculatio n Black ethnicity (21000) coding 4, 4001, 4002, 4003
	Peripheral artery disease		I739, I702, I740-I749			
**Exclusion**						
	Pregnant or of childbearing potential women	31, 53				
	Drug abuse	HES	F19			
	Prior AF ablation or surgical therapy of AF	HES OPS		K621, K622, K522, K23		
	Severe mitral valve stenosis	HES	I342, I050			

		Prosthetic mitral valve	HES OPS		K251-K254	
		Clinically relevant hepatic dysfunction requiring specific therapy	HES	K70-K75		
		Clinically manifest thyroid dysfunction requiring therapy After successful treatment of thyroid dysfunction, patients may be enrolled when their thyroid function is controlled	HES, GP	E05, E06, E30		yes
		Severe renal dysfunction (stage V, requiring or almost requiring dialysis)	HES	Z49, N185		
**Interventions**						
	**Rhythm control therapy**					
		Amiodarone	GP			yes
		Sotalol	GP			yes
		Dronedarone	GP			yes
		Propafenone	GP			yes
		Flecainide	GP			yes
		AF ablation	HES OPS		K621-K622	
	**Rate control therapy: beta-blockers**					
		Metoprolol	GP			yes
		Carvedilol	GP			yes
		Nebivolol	GP			yes
		Bisoprolol	GP			yes

	**Rate control therapy: others**				
		Digitoxin	GP		yes
		Digoxin	GP		yes
		Verapamil	GP		yes
		Diltiazem	GP		yes
**Baseline/others**					
		BMI	21001		
		Other valvular disease	HES	I359; I34	
		Other pulmonary embolism	HES	I26	
**Outcomes**					
	Primary outcome				
		death from cardiovascular causes	40000, 40001	I10 -I15, I20-I25, I26-I28, I30-I52, I70-I79	
		onset stroke	HES	I60-I64	
		hospitalisation heart failure	HES	I50, I110, I130, I132, I249	
		hospitalisation acute coronary syndrome	HES	I21, I22, I24	
	Secondary outcome				
		Number of nights in hospital per year	HES		
		Onset dementia	*HES*	*F00, F01, F03*	

**Adverse events**				
	Onset stroke	HES	I60-I64	
	Death	40000		
Serious adverse event of special interest related to rhythm-control therapy (drugs + ablation)				
	Non-fatal cardiac arrest	HES	I46	
	Drug-induced bradycardia	HES	R001	
	Atrioventricular block	HES	I44	
	Torsades de pointes tachycardia	*HES*	*I4581*	
Serious adverse event related to atrial fibrillation ablation				
	Pericardial tamponade	HES	I314	
	Major bleeding related to atrial fibrillation ablation	HES	T810	
	Blood pressure–related event	HES	T811, T812	
	Syncope	HES	R55	
	Implantation of a pacemaker, defibrillator, cardiac resynchronization device, or any other cardiac device	HES OPS		K721, K591-K592, K596, K601, K605-K607
**Propensity score matching**				
	Age (at the onset of AF)	defined above		

	BMI	defined above		
	CHA_2DS_2-VASc	defined above		
	Chronic kidney disease	defined above		Only ICD-10 terms were used
	Severe coronary artery disease	defined above		Only ICD-10 terms were used
	Diabetes mellitus	defined above		Only ICD-10 terms were used
	Heart failure	defined above		Only ICD-10 terms were used
	Hypertension	defined above		Only ICD-10 terms were used
	Peripheral artery disease	defined above		Only ICD-10 terms were used
	Sex	defined above		
	Stroke/TIA	defined above		Only ICD-10 terms were used
	Valvular heart disease	defined above		Only ICD-10 terms were used
	Alcohol abuse	HES	F10	

	Anticoagulation treatment	GP		yes
	Cancer (Malignancy)	40005, 40006, 40013, HES	C00-C26, C30-C34, C37-C58, C60-C86, C88, C90-C97	
	Chronic obstructive pulmonary disease	HES	J42, J431, J432, J438, J439, J44	
	Dilated cardiomyopathy	HES	I420	
	Dyslipidaemia	HES	E78	
	Gastrointestinal bleeding	HES	K921, K922	
	Gastrointestinal ulcer	HES	K25-K28	
	History of Endocarditis	HES	I33, I38, I39	
	Hyperthyroidism	HES	E05	
	Hypertrophic cardiomyopathy	HES	I421, I422	
	Hypothyroidism	HES	E00-E03	
	Myocardial Infarction	HES	I21, I22, I252	
	Obstructive sleep apnea	HES	G473	
	Osteoporosis	HES	M80, M81, M821, M828	
	Pulmonary embolism	HES	I26	
	Smoking	20116		
**Falsification analysis**				
	Acute appendicitis	HES	K35	
	Alcoholic Liver Disease	HES	K70	

	Asthma	HES	J459
	Benign paroxysmal positional vertigo	HES	H811
	Bladder Cancer	HES	C67
	Bronchiectasis	HES	J47
	Celiac Disease	HES	K900
	Chronic sinusitis	HES	J32
	Colon Cancer	HES	C18
	Epilepsy	HES	G40
	Frozen shoulder	HES	M750
	Glaucoma	HES	H40
	Lung Cancer	HES	C34
	Major fracture	HES	S72, S120, S121, S122, S127, S129, S220, S221, S320, S32
	Meniere's disease	HES	H810
	Multiple Sclerosis	HES	G35
	Otitis media	HES	H65, H66, H67
	Parkinsons Disease	HES	G20
	Prostate Cancer	HES	C61
	Psoriasis	HES	L40
	Renal Cancer	HES	C64
	Sickle Cell Disease	HES	D57
	Type 2 Diabetes Mellitus	HES	E11

Supplemental table for definitions of outcomes and therapies
