# Supplementary material for: Eligibility for early rhythm control in patients with atrial fibrillation in the UK Biobank
Source: Heart. 2022 Jul 14;108(23):1873–80. doi: 10.1136/heartjnl-2022-321196 (PMC9664114; doi:10.1136/heartjnl-2022-321196)
Supplement: Supplementary data [file heartjnl-2022-321196supp001.pdf]

BMJ Publishing Group Limited (BMJ) disclaims all liability and responsibility arising from any reliance placed on this supplemental material which has been supplied by the author(s)

Supplement

Methods

Data Collection

Our main dataset, including the baseline patient characteristics and the first date of AF diagnosis, was obtained based on the UK Biobank first date of AF diagnosis (field 131350) on 15th April 2021. The corresponding HES and GP datasets were collected on 30th March 2021, with the latest diagnosis on HES (any comorbidity) being recorded on 2nd February 2021. First, a search for drug names was performed on the GP scripts, and the results obtained were then enriched with their corresponding BNF codes. Lists of drugs for each BNF code were compiled and checked by SK. A list of drug codes used in this study is provided in the supplementary material. The participants' age, at the time of AF diagnosis, was estimated from the age of the patient at enrollment and calculated using the fields date of admission (field 53), age at recruitment (21022), and the date of AF diagnosis from any source (131350). The diagnosis of diabetes was identified based on HES diagnosis information and/or a glycated hemoglobin (HbA1c – field 30750) level above 48 mmol/mol. The diagnosis of hypertension was provided on the basis of a HES diagnosis information or medications (field 6177 for males, and field 6153 for females, coding 2), or the blood pressure at recruitment (systolic above 140 fields 93 and 4080, diastolic above 80 fields 94 and 4079). Heart failure was denoted based on HES diagnosis information and/or heart failure medications. Chronic kidney disease was identified based on HES diagnosis information, or 15 <= GFR < 60. GFR was calculated using information from fields cystatin-C (30720), creatinine blood (30700 – transformed to mg/dL using factor 0.0113), sex (31), ethnicity (21000 – any value on black group coding 4), and calculated age at diagnosis. Thyroid dysfunction was identified based on HES diagnosis information and/or treatment

with Levothyroxine. All cancer data was collected based on relevant HES ICD-10 codes and the date residing in the UKB Category 100092 field.

Propensity-score matching and missing data

For matching parameters, see the main paper methods section. Matching was performed without replacement. Analysis was performed this way to ensure a simpler analysis, as analysis with replacement would require weighting, and balancing for the repeated participants.[1] The participants were assessed for their standardised mean differences (SMD) to ensure good matching. Although there is no consensus on a good threshold for SMD, some authors use Cohen's definition of 0.2 as small, which has been chosen for this study.[2] Participants were analysed by the stratification of the outcome variable. Diagnoses and patient events that were not in the database were considered that they did not happen, as it is not possible to differentiate between missing and not happened events. Patient matching was performed with only complete data (excluded 6 participants in early rhythm-control, and 81 on usual care).

Follow-up data collection

To estimate the number of hospitalization days, spell data were connected to their episode data. This involved processing the original dataset (over 3.5 million episodes for all participants in the UK Biobank), removing episodes without admission and discharge date (248,368 episodes). For each spell, the earliest and latest date from records were recorded and used as reference for hospitalization. Incident repeat events were obtained from the count of unique spell identifiers where a patient had events 6 months after the date of atrial fibrillation.

Mortality information was obtained from the UK Biobank fields 40000 and 40001. Cardiovascular death was considered based on the following ICD codes: I10-I15; I20-I28; I30-I52; I70-I79. Patient follow-up was obtained from the maximal date of records on GP records, HES, or death. Records that had any of special placeholder dates were ignored (1900-01-01, 1901-01-01, 1902-02-02, 1903-03-03, or 2037-07-07). Two participants with AF marked at the same day of birth were discarded. For the disease onset, a diagnosis in the hospital inpatient data was required to form the primary reason of visit. Survival models were created using Cox proportional hazards regression model, modelling the occurrence of each event depending on the rhythm group.[3] Cox regression is a very well-known tool to study the effect of time on dependent variables. Our models were created with each predicted outcome, time to event (or censoring), against the type of treatment the patients had. In the two experiments run (unmatched and matched data) resulted in the estimated HR without correction (unmatched data) and the HR corrected for similar participants (matched data).

R-packages for statistical analysis

- Zhicheng Du and Yuantao Hao (2019). stddiff: Calculate the Standardized Difference for Numeric, Binary and Category Variables. R package version 3.0.
- https://CRAN.R-project.org/package=stddiff

- G. van Rossum, Python tutorial, Technical Report CS-R9526, Centrum voor Wiskunde en Informatica (CWI), Amsterdam, May 1995."

-] Myles Hollander and Douglas A. Wolfe (1973), Nonparametric Statistical Methods. New York: John Wiley & Sons. Pages 115-120.

- Chambers, J. M., Freeny, A and Heiberger, R. M. (1992) Analysis of variance; designed experiments. Chapter 5 of Statistical Models in S eds J. M. Chambers and T. J. Hastie, Wadsworth & Brooks/Cole.

- Fisher, R. A. (1970). Statistical Methods for Research Workers. Oliver & Boyd.

- Clarkson, D. B., Fan, Y. and Joe, H. (1993) A Remark on Algorithm 643: FEXACT: An Algorithm for Performing Fisher's Exact Test in r x c Contingency Tables. ACM Transactions on Mathematical Software, 19, 484-488. doi: 10.1145/168173.168412.

- R Core Team (2020). R: A language and environment for statistical computing. R Foundation for Statistical Computing, Vienna, Austria. URL https://www.R-project.org/.

- Daniel E. Ho, Kosuke Imai, Gary King, Elizabeth A. Stuart (2011). MatchIt: Nonparametric Preprocessing for Parametric Causal Inference. Journal of Statistical Software, Vol. 42, No. 8, pp. 1-28. URL https://www.jstatsoft.org/v42/i08/

- Garrett Grolemund, Hadley Wickham (2011). Dates and Times Made Easy with lubridate. Journal of Statistical Software, 40(3), 1-25. URL https://www.jstatsoft.org/v40/i03/.

- Matt Dowle and Arun Srinivasan (2020). data.table: Extension of `data.frame`. R package version 1.13.6. https://CRAN.R-project.org/package=data.table

- Therneau T (2021). _A Package for Survival Analysis in R_. R package version 3.2-10, <URL: https://CRAN.R-project.org/package=survival>.

- Terry M. Therneau, Patricia M. Grambsch (2000). _Modeling Survival Data: Extending the Cox Model_. Springer, New York. ISBN 0-387-98784-3.

- Bendix Carstensen, Martyn Plummer, Esa Laara, Michael Hills (2021). Epi: A Package for Statistical Analysis in Epidemiology. R package version 2.44. URL
- https://CRAN.R-project.org/package=Epi

- Bob Gray (2020). cmprsk: Subdistribution Analysis of Competing Risks. R package version 2.2-10. https://CRAN.R-project.org/package=cmprsk

- Alboukadel Kassambara, Marcin Kosinski and Przemyslaw Biecek (2021). survminer: Drawing Survival Curves using 'ggplot2'. R package version 0.4.9.
- https://CRAN.R-project.org/package=survminer

- Claus O. Wilke (2020). cowplot: Streamlined Plot Theme and Plot Annotations for 'ggplot2'. R package version 1.1.1. https://CRAN.R-project.org/package=cowplot

Supplemental Tables

	Not Eligible (5524)	Eligible (22650)
Age (years, 1st-3rd quartile)	63 (58-67)	70 (66-74)
CHA_2DS_2-VASc	1 (1-2)	3 (2-4)
Inclusion criteria		
Age > 75 years	158 (3%)	3373 (15%)
Prior stroke	121 (2%)	2141 (9%)
Or 2 of the folllowing		
Age > 65	1546 (28%)	18075 (80%)
Severe coronary artery disease	519 (9%)	6922 (31%)
Chronic kidney disease	308 (6%)	2236 (10%)
Diabetes mellitus	386 (7%)	4236 (19%)
Heart Failure	579 (10%)	7303 (32%)
Hypertension	3224 (58%)	20775 (92%)
Peripheral artery disease	116 (2%)	998 (4%)
Female sex	1153 (21%)	9552 (42%)
Exclusion criteria		
Severe mitral valve stenosis	45 (1%)	0 (0%)
Prosthetic mitral valve	62 (1%)	0 (0%)
Hepatic dysfunction	526 (10%)	0 (0%)
Thyroid dysfunction untreated	272 (5%)	0 (0%)
Severe renal dysfunction	245 (4%)	0 (0%)
Woman < age 45 (to exclude pregnancy)	25 (0%)	0 (0%)
Drug abuse	10 (0%)	0 (0%)
Prior ablation	228 (4%)	0 (0%)

Supplemental Table 1: Overview of participants included and excluded. Participants with atrial fibrillation for longer than 1 year were not considered**.**

	**Early rhythm-control (N=868)**	**Usual care (N=868)**	**P-value**
Follow-up duration (years)	4.92 (2.98-6.91)	4.13 (1.65-6.69)	
Age (years)	68 (64-70)	68 (65-71)	< 0.001
Body mass index (kg/m²)	28.16 (25.27-31.51)	28.38 (25.73-31.67)	0.063
Days until treatment	44.50 (18-153.75)	-	0.335
Sex (male)	504 (58%)	512 (59%)	-
Chronic kidney disease	67 (8%)	59 (7%)	0.733
Diabetes	112 (13%)	112 (13%)	0.517
Heart failure	522 (60%)	520 (60%)	1.000
Hypertension	797 (92%)	800 (92%)	0.961
Peripheral vascular disease	37 (4%)	36 (4%)	0.860
History of myocardial infarction	118 (14%)	110 (13%)	1.000
Severe coronary artery disease	284 (33%)	270 (31%)	0.619
Stroke/transient ischemic attack	61 (7%)	56 (6%)	0.503
Valvular disease	53 (6%)	50 (6%)	0.702
Dyslipidemia	246 (28%)	243 (28%)	0.915
Obstructive sleep apnea	17 (2%)	15 (2%)	0.859
Chronic obstructive pulmonary disease	44 (5%)	41 (5%)	0.824
Malignancy	135 (16%)	131 (15%)	0.824
History of alcohol abuse	16 (2%)	16 (2%)	0.842
CHA_2DS_2-VASc Score (Interquartile range)	3 (2-4)	3 (2-4)	1.000
Anticoagulation	602 (69%)	524 (60%)	0.554
Beta Blocker	665 (77%)	468 (54%)	< 0.001
Digoxin	126 (15%)	93 (11%)	< 0.001
Sodium channel blockers	177 (20%)	18 (2%)	0.021
Potassium channel blockers	673 (78%)	53 (6%)	< 0.001
Ablation	92 (11%)	0 (0%)	-

Supplemental Table 2: Baseline characteristics of participants receiving early rhythm control or usual care in a propensity matched cohort.

	Total Events		Events/100 patient years		
**Outcome**	**Early rhythm-control (N=874)**	**Usual care (N=8817)**	**Rhythm control**	**Usual care**	**Absolute difference**
Stroke/TIA	98	616	2.43	2.43	0.00
Acute coronary syndrome	62	482	1.53	1.90	-0.36
Worsening of heart failure	414	2888	10.25	11.37	-1.13
**Propensity-score matching**					
	Total Events		Events/100 patient years		
**Outcome**	**Early rhythm-control (N=868)**	**Usual care (N=868)**	**Rhythm control (N=868)**	**Usual care (N=868)**	**Absolute difference**
Stroke/TIA	94	84	2.35	2.47	-0.12
Acute coronary syndrome	61	67	1.53	1.97	-0.44
Worsening of heart failure	414	355	10.35	10.42	-0.07

Supplemental Table 3: Repeated events analysis for participants receiving either early rhythm-control or usual care.

**Overall cohort**		
**Disease**	**HR**	**P-Value**
Alcoholic liver disease	0.22 (0.03-1.62)	0.137
Asthma	0.61 (0.48-0.79)	**<0.001**
Bladder cancer	1.33 (0.78-2.27)	0.301
Bronchiectasis	0.60 (0.35-1.02)	0.058
Celiac disease	0.59 (0.21-1.64)	0.313
Colon cancer	0.42 (0.17-1.03)	0.058
Epilepsy	0.44 (0.24-0.82)	**0.009**
Glaucoma	0.74 (0.48-1.13)	0.158
Lung cancer	1.16 (0.73-1.82)	0.533
Multiple sclerosis	0.25 (0.04-1.87)	0.179
Parkinson's disease	0.74 (0.40-1.34)	0.312
Prostate cancer	0.95 (0.65-1.40)	0.800
Psoriasis	0.66 (0.34-1.26)	0.207
Renal cancer	0.84 (0.30-2.36)	0.733
Sickle cell disease	3.58 (0.32-39.59)	0.298
Diabetes mellitus II	0.63 (0.52-0.76)	**<0.001**
Acute appendicitis	0.74 (0.22-2.44)	0.618
Benign paroxysmal positional vertigo	0.67 (0.20-2.20)	0.507
Chronic sinusitis	0.43 (0.10-1.79)	0.246
Frozen shoulder	0.78 (0.18-3.36)	0.736
Major fracture	0.91 (0.61-1.36)	0.651
Meniere's disease	1.34 (0.39-4.68)	0.631
Otitis media	2.16 (0.70-6.71)	0.182
**Propensity-score matching**		

Disease	HR	P-Value
Alcoholic liver disease	0.85 (0.05-13.66)	0.911
Asthma	0.81 (0.57-1.14)	0.225
Bladder cancer	1.99 (0.82-4.84)	0.129
Bronchiectasis	0.94 (0.46-1.96)	0.877
Celiac disease	1.71 (0.31-9.32)	0.537
Colon cancer	0.88 (0.25-3.03)	0.837
Epilepsy	0.52 (0.25-1.10)	0.089
Glaucoma	1.22 (0.66-2.27)	0.533
Lung cancer	3.24 (1.31-7.99)	**0.011**
Multiple cclerosis	0.29 (0.03-2.77)	0.281
Parkinson’s disease	0.87 (0.39-1.94)	0.737
Prostate cancer	1.21 (0.69-2.12)	0.506
Psoriasis	1.00 (0.39-2.60)	0.999
Renal cancer	3.46(0.39-30.95)	0.267
Sickle cell disease*	-	-
Diabetes mellitus II	0.94 (0.72-1.23)	0.660
Acute appendicitis	0.88 (0.18-4.37)	0.878
Benign paroxysmal positional vertigo	0.67 (0.15-2.97)	0.593
Chronic sinusitis	0.57 (0.01-3.39)	0.533
Frozen shoulder	1.84 (0.17-20.37)	0.617
Major fracture	1.27 (0.70-2.31)	0.433
Meniere’s disease	2.55 (0.27-24.56)	0.417
Otitis media*	-	-

Supplemental Table 4: Falsification analysis on the effect of early rhythm control on random outcomes in the overall cohort and the matched analysis. P-values < 0.05 were considered significant and marked bold. * Insufficient number of samples for analysis.

Supplemental Figure 1: Sensitivity analysis of the safety outcome of a composite of stroke/transient ischemic attack, all-cause death and adverse events related to rhythm control therapy of participants receiving either early rhythm-control or usual care in the overall cohort (A) and a propensity-score matched analysis (B). Shown are Hazard ratios for the composite safety outcome and for its components. Follow-up was limited to March 2020 to exclude the effect of the Covid-19 pandemic.

Supplemental Figure 2: Sensitivity analysis of the efficacy outcome of a composite of stroke/transient ischemic attack, cardiovascular death, acute coronary syndrome and worsening of heart failure of participants receiving either early rhythm-control or usual care in the overall cohort (A) and a propensity-score matched analysis (B). The hazard ratios are presented for the composite efficacy outcome and for its components. Follow-up was limited to March 2020 to exclude the effect of the Covid-19 pandemic.

Supplemental Figure 3: Sensitivity analysis of the safety outcome of a composite of stroke/transient ischemic attack, all-cause death and adverse events related to rhythm control therapy of participants receiving either early rhythm-control or usual care in the overall cohort (A) and a propensity-score matched analysis (B). The hazard ratios are presented for the composite safety outcome and for its components. No participants were excluded for death within 6 months of AF diagnosis and events during those 6 months were included.

Supplemental Figure 4: Sensitivity analysis of the efficacy outcome of a composite of stroke/transient ischemic attack, cardiovascular death, acute coronary syndrome and worsening of heart failure of participants receiving either early rhythm-control or usual care in the overall cohort (A) and a propensity-score matched analysis (B). The hazard ratios are presented for the composite efficacy outcome and for its components. No participants were excluded for death within 6 months of AF diagnosis and events during those 6 months were included.

References:
1. Stuart EA. Matching methods for causal inference: A review and a look forward. Stat Sci 2010;25(1):1-21. (In eng). DOI: 10.1214/09-STS313.
2. Cohen J. Statistical power analysis for the behavioral sciences: Routledge, 2013.
3. Austin PC. Comparing paired vs non-paired statistical methods of analyses when making inferences about absolute risk reductions in propensity-score matched samples. Stat Med 2011;30(11):1292-301. (In eng). DOI: 10.1002/sim.4200.
