# Supplementary figures and images for: Eligibility for early rhythm control in patients with atrial fibrillation in the UK Biobank
Source: Heart. 2022 Jul 14;108(23):1873–80. doi: 10.1136/heartjnl-2022-321196 (PMC9664114; doi:10.1136/heartjnl-2022-321196)

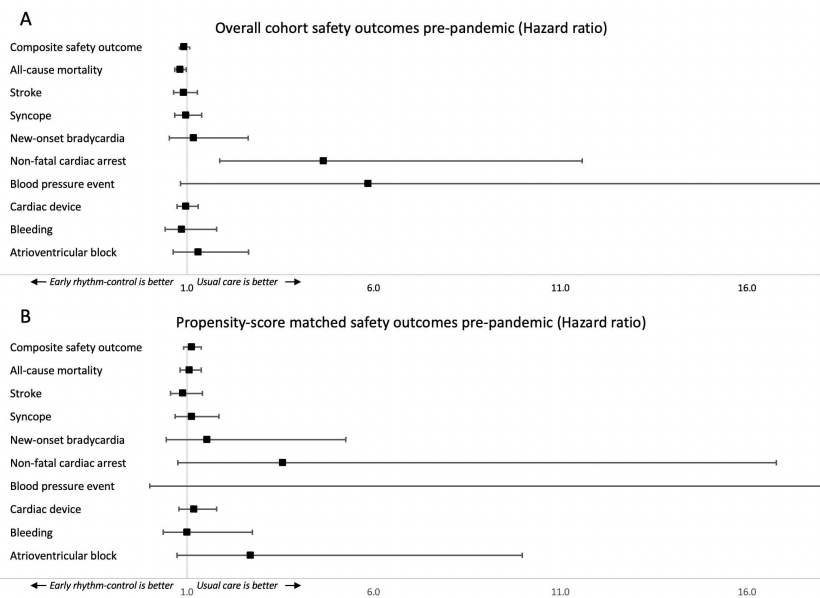

Supplement: Supplementary data [file heartjnl-2022-321196supp003.pdf]

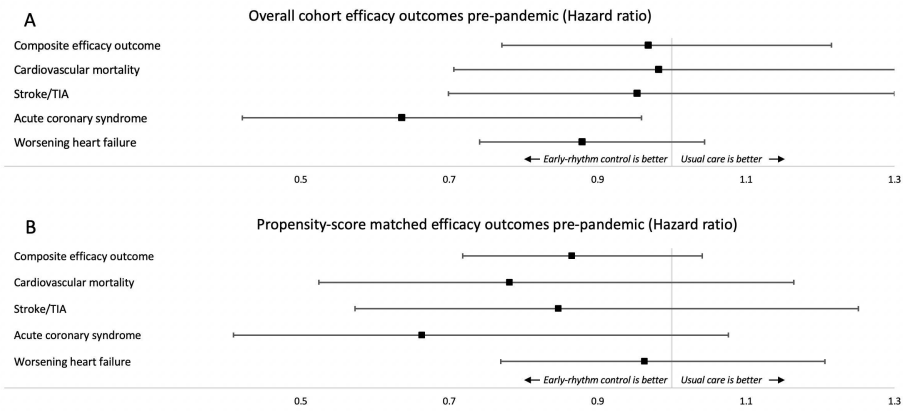

Supplement: Supplementary data [file heartjnl-2022-321196supp004.pdf]

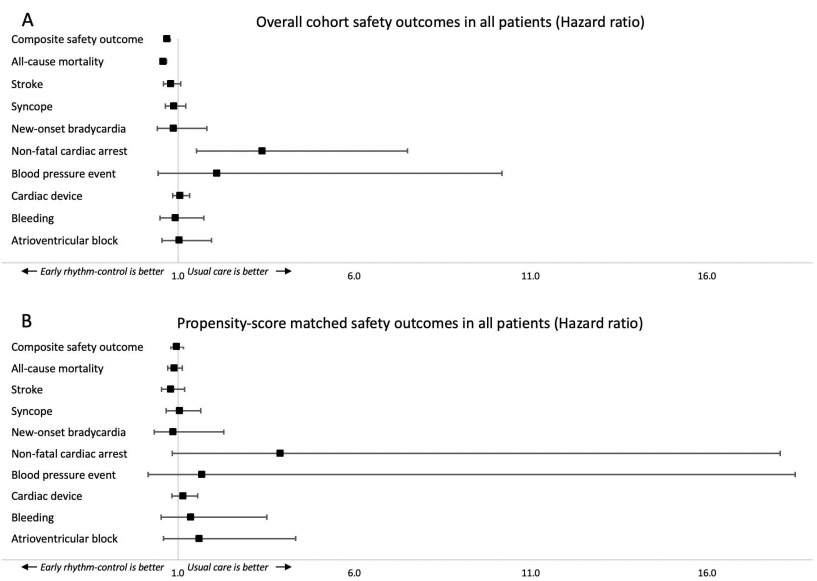

Supplement: Supplementary data [file heartjnl-2022-321196supp005.pdf]

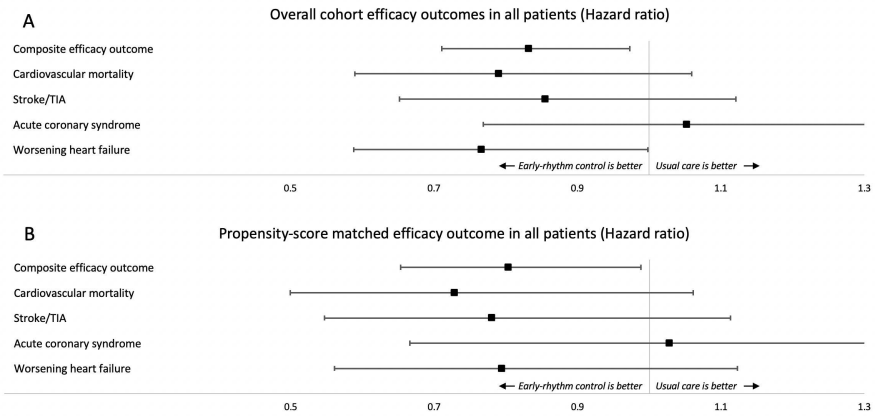

Supplement: Supplementary data [file heartjnl-2022-321196supp006.pdf]
